# End-binding protein 1 regulates the metabolic fate of CD4^+^ T lymphoblasts and Jurkat T cells and the organization of the mitochondrial network

**DOI:** 10.3389/fimmu.2023.1197289

**Published:** 2023-07-13

**Authors:** Álvaro Gómez-Morón, Silvia Requena, Clara Pertusa, Marta Lozano-Prieto, Diego Calzada-Fraile, Camila Scagnetti, Inés Sánchez-García, Ana Adela Calero-García, Manuel Izquierdo, Noa B. Martín-Cófreces

**Affiliations:** ^1^ Immunology Service, Instituto de Investigación Sanitaria del Hospital Universitario La Princesa (IIS-Princesa), Madrid, Spain; ^2^ Immunology, Oftalmology and Otorrinolaryngology Dept., School of Medicine, Universidad Complutense de Madrid (UCM), Madrid, Spain; ^3^ Vascular Pathophysiology, Laboratory of Intercellular Communication, Fundación Centro Nacional de Investigaciones Cardiovasculares-Carlos III (CNIC), Madrid, Spain; ^4^ Videomicroscopy Unit, Instituto de Investigación Sanitaria del Hospital Universitario La Princesa, IIS-Princesa, Madrid, Spain; ^5^ Neumology Service, Hospital Universitario de la Princesa, UAM, IIS-Princesa, Madrid, Spain; ^6^ Instituto de Investigaciones Biomédicas "Alberto Sols", Consejo Superior de Investigaciones Científicas-Universidad Autónoma de Madrid (CSIC-UAM), Madrid, Spain; ^7^ Centro de Investigación Biomédica en Red de Enfermedades Cardiovasculares (CIBERCV), Madrid, Spain

**Keywords:** EB1, mitochondria, T cell receptor, glycolysis, cell asymmetry, cytoskeleton

## Abstract

The organization of the mitochondrial network is relevant for the metabolic fate of T cells and their ability to respond to TCR stimulation. This arrangement depends on cytoskeleton dynamics in response to TCR and CD28 activation, which allows the polarization of the mitochondria through their change in shape, and their movement along the microtubules towards the immune synapse. This work focus on the role of End-binding protein 1 (EB1), a protein that regulates tubulin polymerization and has been previously identified as a regulator of intracellular transport of CD3-enriched vesicles. EB1-interferred cells showed defective intracellular organization and metabolic strength in activated T cells, pointing to a relevant connection of the cytoskeleton and metabolism in response to TCR stimulation, which leads to increased AICD. By unifying the organization of the tubulin cytoskeleton and mitochondria during CD4^+^ T cell activation, this work highlights the importance of this connection for critical cell asymmetry together with metabolic functions such as glycolysis, mitochondria respiration, and cell viability.

## Introduction

End binding protein 1 (EB1) is a microtubule (MT) plus-end tracking protein (+TIP) which associates with MTs and regulates their dynamics, promoting their growth and preventing catastrophes. It was initially found to interact with the COOH of the tumour suppressor adenomatous polyposis coli (APC), although this interaction is not required for MT polymerization (1). As a +TIP, it recruits and nucleates a network with other proteins of the same family, such as CLASP2 (2) and CLIP-170 (3), amplifying their effects in the stabilization or rescue of MT depolymerization. EB1 binds to molecular motors, such as KIF4 (4) or to the p150^Glued^ subunit of the adaptor dynactin complex (3), enabling the movement of cargoes throughout the cytoplasm.

EB1 is instrumental in guiding the spindle-kinetochore-associated 1-3 (Ska1-3) on MTs in vertebrate cells during cell division (5, 6). Its function as a guiding molecule was first observed during nicotinic synapse signalling, being involved in the transport and localization of the α3 Nicotinic Acetylcholine receptors (α3*nAChRs) in postsynaptic sites of neurons (7).

In lymphocytes, the immune synapse (IS) occurs upon cognate interaction of the T cell receptor (TCR) and the peptide-major histocompatibility complex (pMHC) of an antigen presenting cell (APC). This event requires the presence of CD3 molecules in close proximity to the TCR (TCR/CD3 complexes) (8). The phosphorylation of tyrosines in immunotyrosine based activating motifs (ITAMs) by Lck and Fyn kinases will eventually lead to the recruitment of other kinases and intermediates (9). These initial reactions will occur in a plasmatic membrane-underlying signalling platform, referred to as the central supramolecular activation clusters (c-SMAC) (10). The c-SMAC will ensure the correct propagation of the activating signal by regulating F-actin and MTs dynamics and organization. EB1 was shown to direct the encounter between CD3ζ-enriched vesicles with the linker of activated T cells (LAT) through its interaction with CD3ζ, contributing to the sustained activation of the latter and PLCγ1 (11).

During the IS, microtubules (MTs) that grow from the translocated centrosome or MT-organizing center (MTOC) act as tracks for the transport of organelles and vesicles, such as mitochondria, which supply energy for the activated cell (12). Localised diacylglycerol (DAG) at the IS was initially discovered as the driving force for centrosome reorientation (13). There are other described mechanisms, involving phosphorylated stathmin (an ERK substrate) (14) and EB1 interaction with the centrosomal casein kinase I delta (CKIδ) (15). Regarding MT and centrosome polarization, the role of kinesin KIF21B has been shown as a key regulator of MT length for proper centrosome relocation (16).

Upon TCR stimulation, it was demonstrated that an intact MT cytoskeleton is required for mitochondrial network reorganization in CD4^+^ T lymphocytes with silenced chaperonin-containing TRiC (CCT) complex (17). In addition, dynamin-related protein 1 (Drp1), a mitochondrial fission factor was found to participate in the localization and activity of the mitochondria at the peripheral supramolecular activation cluster (p-SMAC) and c-SMAC (18). The proper positioning of mitochondria at the IS fuels ATP production and regulates Ca^2+^ influx, required for T cell activation, through their co-localization with ORAI channels (19–22). Indeed, the asymmetric redistribution at the IS of plasma membrane Ca^2+^ ATPases (PMCAs), which regulate local and cytoskeletal Ca^2+^ levels, has been described (12). With respect to EB1, it was shown to bind STIM1 and play a role in the prevention of Ca^2+^ overload, which is associated with apoptosis (23, 24).

All these findings regarding the role of EB1 role in the IS prompted us to investigate its potential metabolic impact during T cell activation. In this study, an impaired organization of the microtubular network, which impacts in the polarization of mitochondria at the IS, is observed in EB1 silenced Jurkat T cells and CD4^+^ T lymphoblasts. Those defects correlate with altered cellular respiration and glycolytic function of mitochondria, which underscore the link between MT dynamics and metabolic processes in T cell activation.

## Results

### EB1-enriched plus TIPs of microtubules localize with mitochondria in polarized lymphoblasts

**Figure 1:**
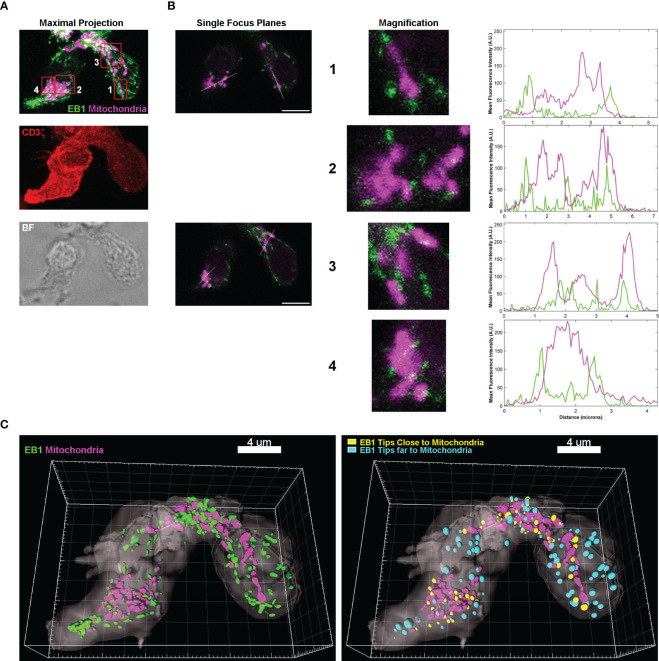
EB1 is in close proximity to the mitochondria at the +TIPs of microtubules. **(A)** Images are confocal maximal projections showing EB1 (green), mitochondria (magenta) and CD3ζ (red) localization in huCD4^+^ T lymphoblasts. A brightfield (BF) image is shown. **(B)** Images are single focus planes from stack in **(A)**, corresponding to mitochondria in insets 1-4. Magnifications of insets are shown. Graphs, profiles of mean fluorescence intensity (MFI) along the white lines. **(C)** 3D reconstructions of the cells with Imaris, including (left image) the cell volume (CD3 ζ), mitochondria (Mitotracker Orange) and EB1 (green) volumes. Right, 3D reconstruction including a map of distances between the centre of mass of EB1 volumes and the mitochondria. Calculation of corresponding volumes are based on MFI. Yellow, close spots; cyan, far spots; threshold is 0.5 μm from the centre of the spot. See also **Supplementary Figure 1**.

The study of EB1 localization in primary human CD4^+^ T lymphoblasts through immunofluorescence showed that CD3^+^ cells were polarized (CD3ζ/CD247 is showed in red in the maximal projection in **Figure 1A**), with a prominent uropod at the rear of the cell that normally contains the centrosome. CD4 and CD3 expression of these cells were analysed by flow cytometry (**Supplementary Figure 1**). This organelle appears to be associated with a great concentration of EB1 (green, **Figure 1A**). EB1 is also detected as tips localized at the end of the MTs (**Figure 1A**), as described (11). In these cells the mitochondria were labelled with the specific probe Mitotracker Orange, and were found mainly polarized around the centrosome, with few organelles located near the leading edge of the cell (magenta, **Figure 1A**). A more detailed examination of the mitochondria revealed that they tend to localize in close proximity to EB1-enriched tips, as observed in the profiles of fluorescence from the insets from single focus planes of the cells (**Figure 1B**). The 3D reconstruction of two CD4^+^ T lymphoblasts from (**Figure 1A**) showed the relative localization of mitochondria and EB1-enriched tips (**Figure 1C**, left). Also, most of mitochondria in cells have nearby EB1-enriched tips (yellow spots in **Figure 1C**, right), with a probabilistic frequency of 0.8824 (88.24%; n=10) for finding a mitochondria showing with an EB1-enriched tip close (i.e.: most mitochondria are near of EB1-enriched tips in cells).

### EB1 is required to sustain mitochondrial membrane potential

**Figure 2:**
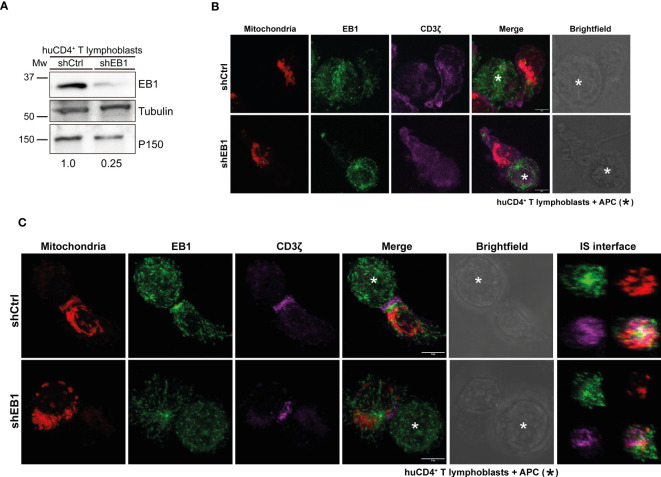
EB1 is required to organize the immune synapse. **(A)** Immunoblot showing KD of EB1 expression in huCD4^+^ T lymphoblasts. Loading controls, α-tubulin and p150*^Glued^*. **(B)** Confocal images of control conjugates of shCtrl and shEB1 huCD4^+^ T lymphoblasts and Raji cells (APCs). Red, mitochondria; green, EB1; magenta, CD3ζ. APC is marked with an asterisk (*). Maximal projections are shown. Bar, 10 μm. **(C)** Control (shCtrl) and shEB1 huCD4^+^ T lymphoblasts forming synapses (IS) with SEE-preloaded APCs. APC is marked with an asterisk (*). Images are as in **(A)**. ZX re-slices of the synaptic area (IS interface) are shown. Bar, 10 μm.

To address the potential role of this proximity of EB1 and mitochondria, EB1 was silenced through transfection of shRNA-encoding plasmids in SEE-specific human primary CD4^+^ T lymphoblasts (**Figure 2A**). Cells were labelled with Mitotracker orange 48 h post-transfection (red in **Figure 2A**). CD4^+^ T cells were then conjugated to unloaded Raji B cells (antigen-presenting cells; APCs) (**Figure 2B**) or SEE-preloaded APCs to induce synapse formation (**Figure 2C**). Imaging through confocal microscopy showed that EB1 expression decreased in shEB1 KD cells (EB1 KD cells thereafter, **Figures 2B, C**, lower panels), as observed with Western blot (**Figure 2A**). The mitochondrial membrane potential was decreased in EB1 KD cells, as shown by lower Mitotracker Orange staining (red; **Figure 2C**). Also, CD3ζ showed decreased polarization at the contact area with the APC (magenta; **Figure 2C**), as previously described (11).

In resting cells, CD3ζ showed an unspecific distribution in the plasma membrane in control and EB1 KD cells. However, EB1 KD T lymphoblasts showed decreased mitochondrial membrane potential compared to shCtrl cells. Upon activation with SEE, EB1 KD T lymphoblasts showed lower polarization of CD3ζ at the IS, with decreased mitochondrial polarization to the IS and decreased mitochondrial membrane potential in contrast with shCtrl T lymphoblasts. The reconstruction of the IS at the T-APC interface (**Figure 2C**, right panels) demonstrates that the organization of EB1 KD cells is affected, with CD3ζ distributed outside the more central area (the so called c-SMAC in mature T-B contacts) (10), compared to shCtrl cells. Also, mitochondria, that appear localized at the p-SMAC in shCtrl cells as described (18), are de-localized in EB1 KD cells. The centrosome is not properly polarized in these cells; there is also a decrease in EB1 accumulation in this organelle. These data corroborate that the silencing of EB1 protein affects the positioning of the TCR/CD3 complex at the contact site between T lymphoblasts and APCs (25), but also that there is a defective organization of the MT cytoskeleton in these cells. This is achieved by means of faulty centrosome polarization, which impairs the reorganization of the mitochondrial network. Indeed, mitochondria decreases their membrane potential, important for T cell activation (17).

### EB1 organizes the immune synapse in T cells

**Figure 3:**
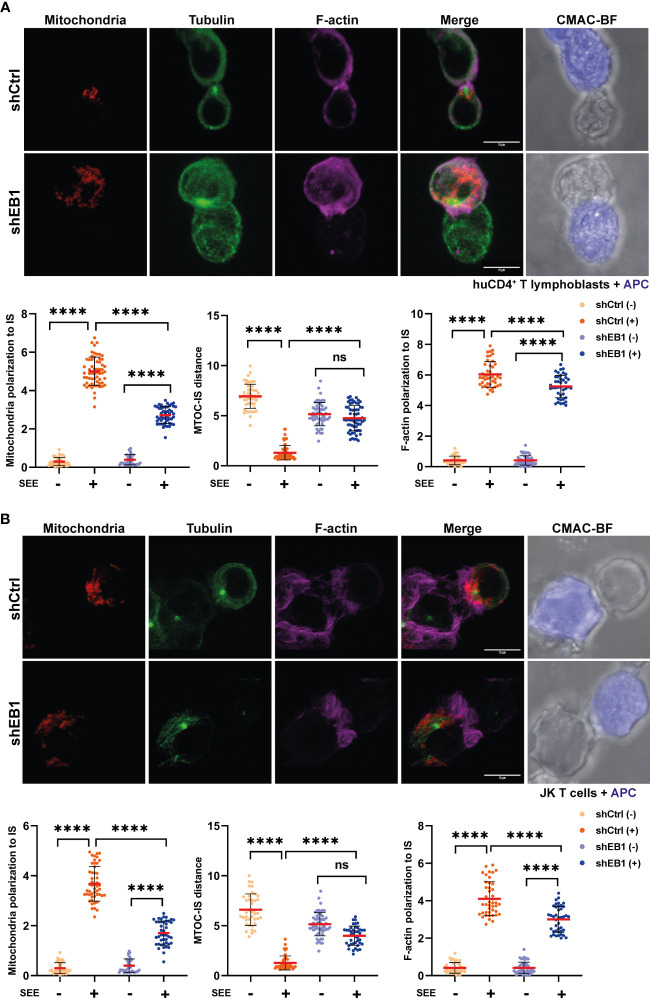
EB1 is necessary for proper synaptic polarization of the cytoskeleton and mitochondria. **(A, B)** Confocal images of synapses formed between shCtrl and shEB1 **(A)** huCD4^+^ T lymphoblasts or **(B)** Jurkat E6-1 T cells with SEE-preloaded Raji cells (APCs). Red, mitochondria; green, α-tubulin; magenta, F-actin; blue, CMAC (Raji cell). Maximal projections are shown. Bar, 10 μm. Graphs, quantification of centrosome distance to the APC, and ratios of IS polarization of mitochondria and F-actin (data are mean ± SD; **(A)** shCtrl, n = 45 and shEB1, n = 55, two independent experiments; **(B)** shCtrl, n = 52 and shEB1, n = 65, three independent experiments. Mann-Whitney test. ns, non-significant, ****, p<0.0001. See also **Supplementary Figure 2**.

Next, α-tubulin (green), F-actin (purple) and mitochondria (red) were assessed in resting and SEE-activated CD4^+^ T lymphoblasts (**Figure 3A**) and Jurkat E6-1 T cells (**Figure 3B** and **Supplementary Figure 2**). Clones of Jurkat E6-1 T cells stably expressing control (shCtrl) or EB1-specific (shEB1) shRNAs were assessed in parallel to primary T lymphoblasts. shCtrl and shEB1 cells showed similar expression of activators and adhesion receptors CD3, CD4 and integrins such as LFA-1 and VLA-4 (**Supplementary Figures 2A, B**). These cells showed consistent reduction in EB1 expression (**Supplementary Figure 2C**). Resting cells did not show apparent differences regarding α-tubulin although the MFI (Mean fluorescence intensity) for mitochondria and F-actin showed a slight decrease in EB1 KD cells (**Supplementary Figures 3A, B**). EB1 KD cells did not relocate the centrosome when labelled with α-tubulin (**Figure 3A**), corroborating the observed effect with EB1 label (**Figure 2**), and showing increased centrosome-IS distance than shCtrl cells upon activation (**Figures 3A, B**). Mitochondria and F-actin polarity ratio also decreased in these cells (**Figures 3A, B**). Altogether, these results point to the importance of EB1 for proper asymmetry organization in human primary T lymphoblasts, showing defective polarization of the mitochondria and microtubule networks to the IS.

### AKT-mTOR-S6 signalling pathway is affected in EB1- KD T cells

**Figure 4:**
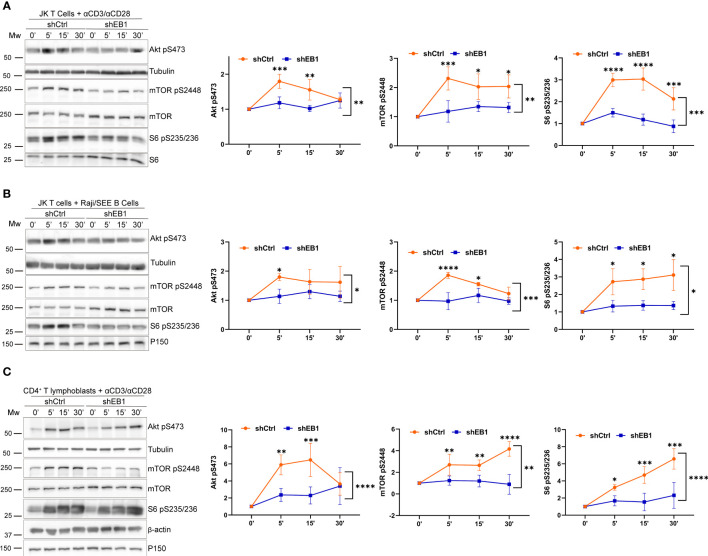
mTOR regulation requires EB1 in T cells. **(A–C)** Phosphorylation of Akt (S473), mTOR (S2448) and S6 (S235/S236) in **(A)** shCtrl and shEB1 Jurkat E6-1 T cells stimulated with αCD3/αCD28 tetramers, **(B)** shCtrl and shEB1 Jurkat E6-1 T cells conjugated with SEE-pulsed Raji B cells, and **(C)** shCtrl and shEB1 huCD4^+^ T lymphoblasts stimulated with αCD3/αCD28 tetramers. β-actin or α-tubulin were used as loading controls. Graphs, quantification of densitometries of bands; data are normalized to non-stimulated control. Data are mean ± SD; **(A, B)**, n = 3; **(C)** n=4; Two-way ANOVA.*, p<0.05; **, p<0.01; *** p<0.001; **** p<0.0001.

Based on the previous findings related to mitochondrial polarization, the metabolic regulatory and signalling pathways involving the activation of AKT, mTOR and S6 in Jurkat E6-1 lymphoblastoid T cells were explored. For this purpose, Jurkat E6-1 stably expressing shEB1 or shCtrl were activated with α-CD3/α-CD28 tetramers or with SEE-preloaded APCs at a 10:1 ratio. The phosphorylation of S473 in AKT, S2448 in mTOR and S235/236 in S6 was analysed at the indicated times (**Figures 4A, B**). These phosphorylations indicate activity for these proteins (26). Their phosphorylation kinetics suggest that EB1 KD cells induce defective activation of these pathways, which indicates a lower metabolic strength for these cells in response to TCR activation.

The activation of mTOR, S6 and AKT was also defective in EB1 KD primary T lymphocytes, which showed decreased phosphorylation (**Figure 4C**). mTOR complex 1 (mTORC1) activates the pathways leading to activation of S6, which is required to initiate translation early upon TCR and CD28 activation in human CD4^+^ T cells (25). Protein translation is probably one of the most demanding metabolic processes, with high ATP requirements. Therefore, the stimulation of the oxidative phosphorylation (OXPHOS) in mitochondria, also regulated by mTORC1, is relevant for T cell metabolic fate. Indeed, Akt is a regulator of mTORC1 activity, therefore favouring T cell activation (27).

**Figure 5:**
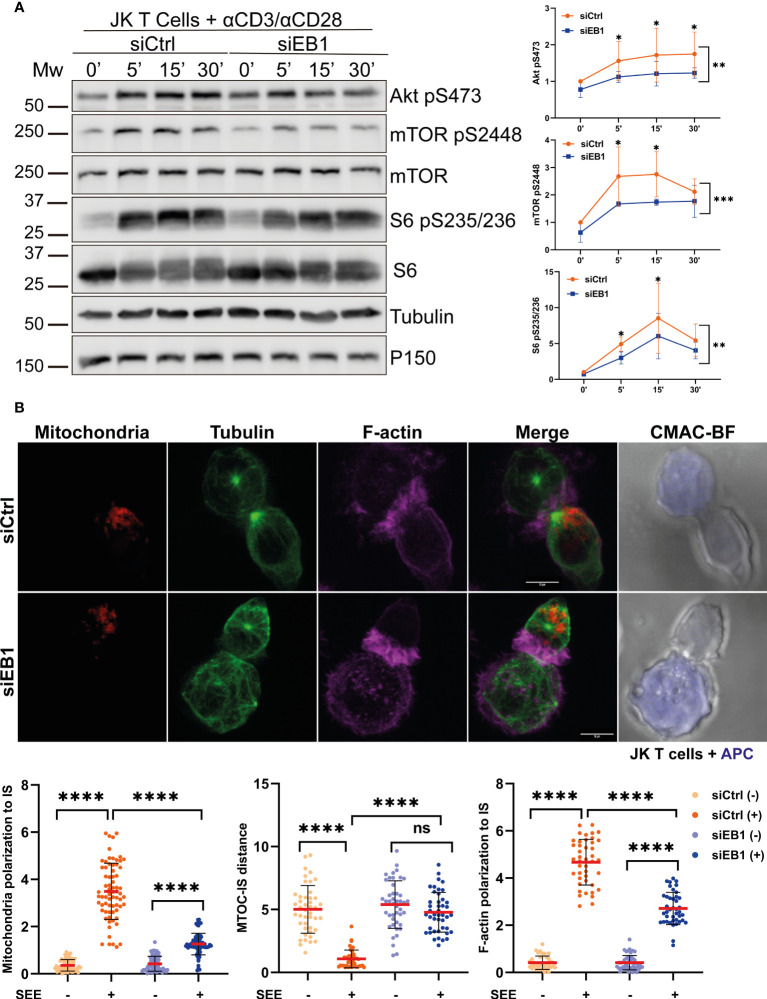
EB1 transient silencing produces similar effects than stable knock-down in Jurkat T cells. **(A)** Phosphorylation of Akt (S473), mTOR (S2448) and S6 (S235/S236) in siCtrl and siEB1 Jurkat E6-1 T cells stimulated with αCD3/αCD28 tetramers. Graphs, quantification of densitometries of bands; data are normalized to non-stimulated control. Data are mean ± SD; n = 3; Two-way ANOVA.*, p<0.05; **, p<0.01; *** p<0.001; **** p<0.0001. **(B)** Confocal images of synapses formed by siCtrl or siEB1 Jurkat E6-1 T cells and SEE-preloaded Raji cells (APCs). Red, mitochondria; green, α-tubulin; magenta, F-actin; blue, CMAC (Raji cell). Maximal projections are shown. Bar, 10 μm. Graphs, quantification of centrosome distance to the APC, and ratios of IS polarization of mitochondria and F-actin. Data are mean ± SD shCtrl, n = 48 and shEB1, n = 56, two independent experiments. Mann-Whitney test. ns, non-significant; ****, p<0.0001. See also **Supplementary Figure 2D**.

The requirement of EB1 for T cell activation was also assessed in the Jurkat cell model by silencing EB1 through siRNA transfection (siEB1, **Figure 5**). The activation of mTOR pathway was defective in these cells that showed decreased mTOR, AKT and S6 phosphorylation, pointing to decreased protein translation upon TCR activation and CD28 co-stimulation (**Figure 5A**). Indeed, EB1 transient knock-down also prevented centrosome repositioning at the IS, together with F-actin and mitochondria polarization (**Figure 5B**), therefore supporting the role of EB1 in maintaining the asymmetry at the IS.

### EB1 regulates oxygen consumption in the mitochondria

**Figure 6:**
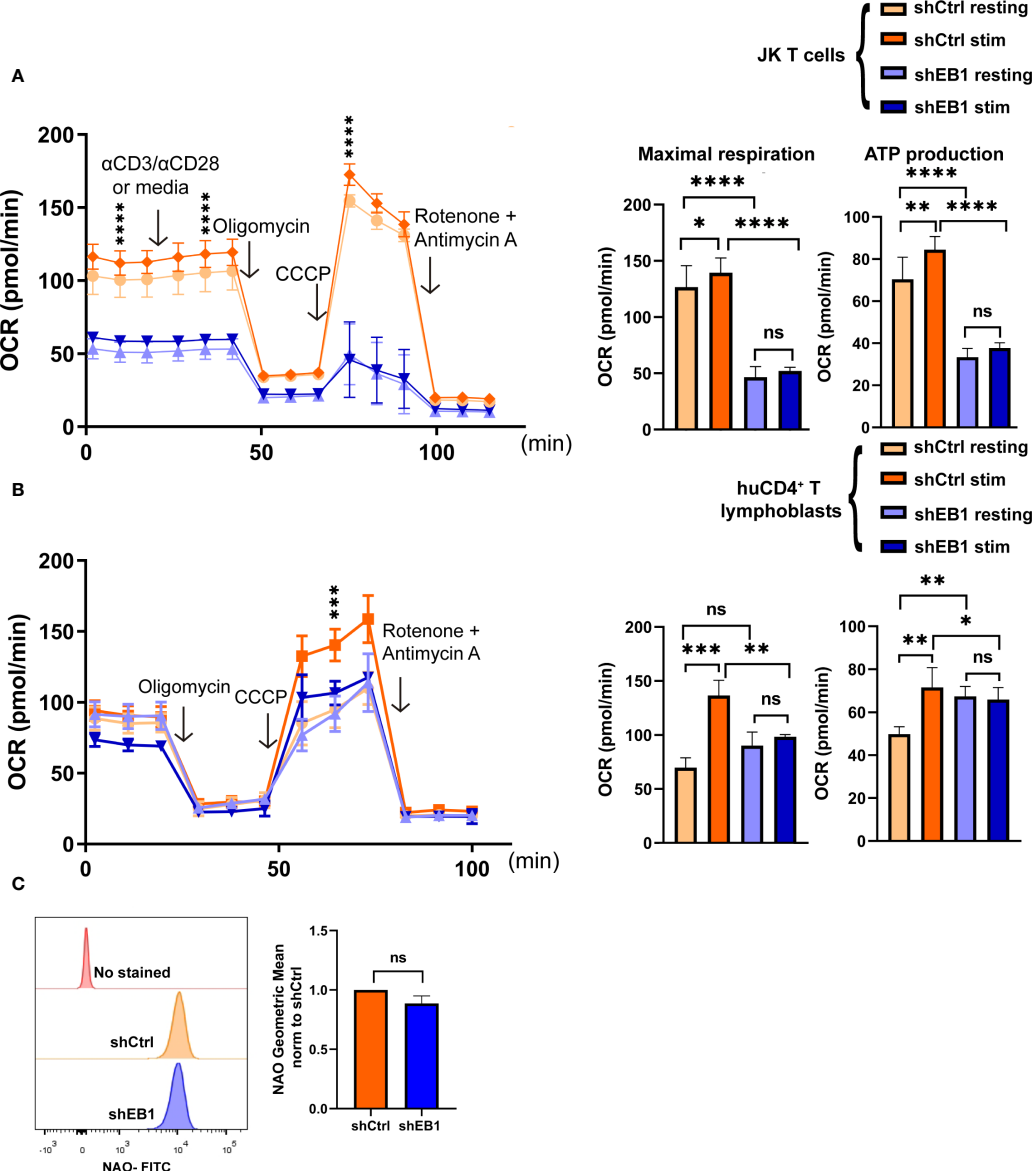
EB1 regulates oxygen consumption rate in mitochondria. **(A, B)** Mitochondria oxygen consumption rate (OCR) in resting and stimulated shCtrl and shEB1 **(A)** Jurkat E6-1 T cells and **(B)** huCD4^+^ T lymphoblasts. Stimulation was with αCD3/αCD28 tetramers. Oligomycin, CCCP and rotenone plus antimycin A were injected as indicated. Right graphs show maximal respiration and ATP production, respectively. Data are mean ± SEM from 5 **(A)** and 4 **(B)** technical replicates from 3 healthy donors. **(A)**, shows a representative experiment out of 4. Linear mixed model was used to analyse differences in OCR and two-way ANOVA test for maximal respiration and ATP production. *, p<0.05; **, p<0.01; *** p<0.001; **** p<0.0001; ns, non-significant. **(C)** Mitochondrial mass in shCtrl and shEB1 Jurkat E6-1 T cells. The graph shows the geometric mean of Nonyl acridine orange (NAO) fluorescence. Data normalized to shCtrl. Graph, mean ± SD; n=3. Unpaired t-test; ns: nonsignificant. See also **Supplementary Figure 4** for gating strategy.

**Figure 7:**
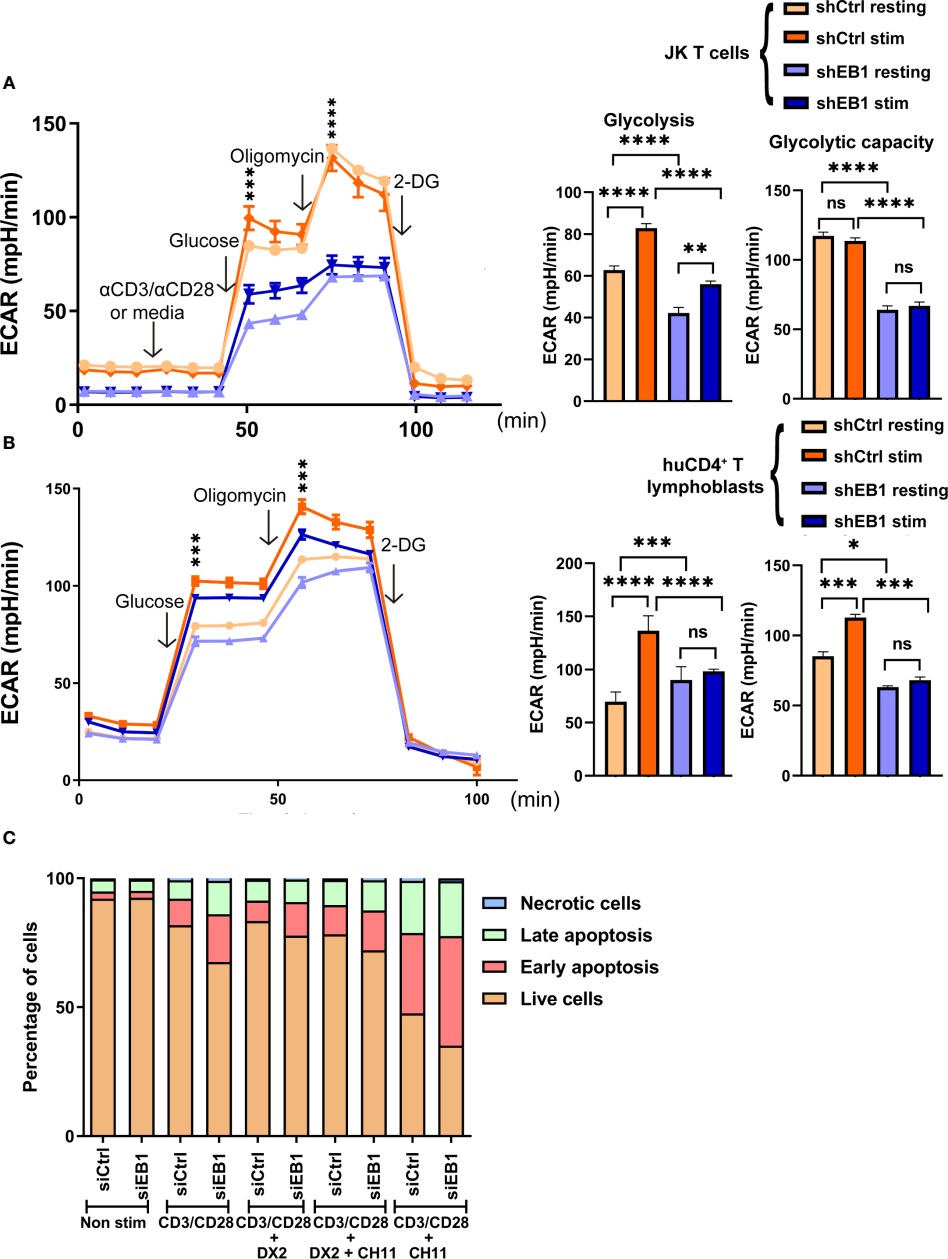
EB1 is required for correct glycolytic response in T cells. **(A, B)** Glycolytic response through extracellular acidification rate (ECAR) in resting or stimulated shCtrl and shEB1 **(A)** Jurkat E6-1 T cells and **(B)** huCD4^+^ T lymphoblasts. Stimulation was with αCD3/αCD28 tetramers. Glucose, oligomycin and 2-deoxyglucose (2-DG) were injected as indicated. Graphs show the rate of glycolysis and the glycolytic capacity. Data are mean ± SEM from 5 **(A)** and 4 **(B)** technical replicates from 3 healthy donors. **(A)**. shows a representative experiment out of 4. Linear mixed model was used to analyse differences in ECAR and two-way ANOVA test for Glycolysis and Glycolytic capacity. *, p<0.05; **, p<0.01; *** p<0.001; **** p<0.0001; ns, non-significant. **(C)** Cell viability after TCR stimulation and CD28 co-stimulation by flow cytometry. Percentage of live (orange), early apoptotic (red), late apoptotic (green) and necrotic (blue) cells are shown for control and EB1-silenced Jurkat E6-1 T cells. Graph, mean from n=3. See **Supplementary Figure 4** for gating strategy in flow cytometry and representative examples and **Supplementary Figure 5** for statistical analysis.

To address the possible consequences of EB1 KD in the regulation of metabolic fate of T cells, cellular respiration was assessed in terms of oxygen consumption rate (OCR) and the extracellular acidification rate (ECAR) during glucose feeding (**Figures 6**, **7**). These experiments inform about the ability of T cells to fuel the cell processes stimulated by TCR activation and co-stimulation through CD28 (17). OCR response was measured in resting conditions or after stimulation with α-CD3/α-CD28 tetramers in shCtrl and shEB1 Jurkat E6-1 T cells (**Figure 6A**) or in huCD4^+^ T lymphoblasts (**Figure 6B**). EB1 KD Jurkat E6-1 T cells showed lower OCR at baseline and did not respond to activation (**Figure 6A**). Also, their maximal respiration did not increase with stimulation, while shCtrl Jurkat E6-1 T cells showed increased basal and maximal respiration in response to stimulation. These results were also observed in CD4^+^ T lymphoblasts from three healthy donors, which were similarly affected by EB1 KD (**Figure 6B**).

Based on these findings, the mitochondrial mass was measured with Nonyl Acridine Orange by flow cytometry to address whether a decreased mitochondrial mass was responsible of this lack of response, but no significant differences were found for EB1 KD cells (**Figure 6C** and **Supplementary Figure 4**).

### EB1 is required for correct glycolytic response in T cells

The glycolytic function of cells KD for EB1 was examined to address whether this lack of energy generation at the mitochondria by using glucose to fuel the process was compensated by lactic acid production from pyruvate. As for OCR experiments, T lymphoblasts and Jurkat E6-1 cells were probed to measure the extracellular acidification rate (ECAR) in glycolysis stress tests (**Figures 7A, B**). Resting and activated cells with α-CD3/α-CD28 tetramers were monitored; acute injection of glucose allowed to observe that EB1 KD cells show lower glycolysis, together with decreased glycolytic reserve and capacity than shCtrl cells, either in the case of Jurkat E6-1 T cells (**Figure 7A**) or primary human CD4^+^ T lymphoblasts (**Figure 7B**). Stimulation increased glycolysis in EB1 KD JK E6-1 cells but not in EB1 KD CD4^+^ T lymphoblasts, which indicates that EB1 KD Jurkat E6-1 cells can respond to TCR stimulation and CD28 co-stimulation but EB1 KD CD4^+^ T lymphoblasts do not. On the other hand, decreased response to stimulation in glycolytic capacity is observed in either EB1 KD Jurkat E6-1 cells or CD4^+^ T lymphoblasts.

### EB1 protein influences cell viability upon activation in T cells

To analyze the effect of mitochondria network deregulation by EB1 KD, cell apoptosis was studied upon TCR activation and co-stimulation in Jurkat E6-1 cells transfected with control siRNA or EB1 siRNA (**Figure 7C** and **Supplementary Figure 5**). This activation-induced cell death (AICD) is mediated by TCR-controlled *de novo* Fas ligand expression and subsequent autocrine FasL binding to Fas (28). At baseline, the percentage of live cells (Annexin^-^Ghost Dye^-^) was equal between control and EB1 silenced cells (92%). After activation with αCD3 and αCD28, there was a statistically significant difference in the percentage of EB1-silenced live cells, when compared to control live cells (67% vs. 81%; p<0.001), and this difference was maintained after the addition of the anti-CD95 (anti-Fas) apoptotic inducer CH-11 monoclonal antibody (35% vs. 47%; p<0.01; **Supplementary Figure 6**). This reduction in the percentage of EB1 silenced live cells was also accompanied by a statistically significant increase in the percentage of cells undergoing early (Annexin^+^Ghost Dye^-^) or late (Annexin^+^Ghost Dye^+^) apoptosis after αCD3/αCD28 activation. Besides, the apoptotic inducer CH-11 reinforced this difference in the augmented percentage of early apoptotic EB1 silenced cells with respect to control cells (42 vs. 31%; p<0.001). Pre-incubation with the anti-Fas antagonistic antibody DX2 (28)inhibited both TCR/CD28 -controlled AICD and agonistic anti-Fas CH11-induced cell death, demonstrating the contribution of FasL/Fas system to the observed AICD. These results suggest that EB1 KD increases activation-induced cell death (AICD).

## Discussion

This work has addressed the role of EB1 in T cell metabolic fate during immune synapse activation through TCR activation and CD28 co-stimulation. It has been proposed that the T cell metabolism and the adoption of asymmetric fate at the IS are connected (25). Part of the T cell asymmetry at the IS consists on the induced intracellular reorganization by TCR activation. Human EB1 was originally cloned using as a bait the COOH terminus of adenomatous polyposis coli (APC), a tumour suppressor (29). The binding site of EB1 in APC was required to allow wound-edge cells to reorient their centrosomes and correctly polarize the cells to divide and migrate (30). Indeed, APC is required to interact with Dlg1, another polarity regulator involved in the reorganization of T cells during synaptic contacts. They are both required for MT network organization at the IS, and for T cell receptor-triggered activation through the NFAT transcription factor (31, 32). EB1 protein regulates the translocation of the centrosome to the IS, the organization of the MT network, and the regulation of the vesicular traffic to allow T cell activation, which depended on the ability of EB1 to interact with the CD3 complex (11). Previous results showed that regulation by casein kinase 1δ (CKIδ) is relevant for this process, through the recruitment of p150*^glued^* and heterodimers of EB1 and EB3. These heterodimers are then phosphorylated by CKIδ allowing efficient polarization of the centrosome to the IS (15). p150*^glued^* is a subunit of the motor adaptor dynactin, that helps dynein to polarize the centrosome and its associated Golgi apparatus and vesicular system to the IS in the T cell (33), and also mitochondria (18). Mitochondria orchestrate diverse fundamental cellular functions, including respiration, calcium homeostasis, reactive oxygen species generation, and programmed cell death (34, 35). Therefore, determining the mitochondrial regulatory mechanisms in order to understand bioenergetics and the role of mitochondria is important for T cell biology. Components of the cytoskeleton play a vital role in the structural and functional organization of the mitochondria, including mitochondrial morphology, dynamics, motility, and intracellular arrangement. Long-range transport has been mainly studied in neurons (36, 37). To this end, the coupling of motor proteins such as kinesins and dyneins to MT forming a complex together with the mitochondria outer membrane GTPase Miro and the adaptor protein Milton/TRAK is required (38, 39). Mitochondrial traffic is closely regulated by intracellular calcium levels, whose increase inhibits kinesin-dependent anterograde and dynein-dependent retrograde movements (40) both involved in mitochondria reorganization. In this regard, Miro reorganize mitochondria during T cell transmigration and this is dependent on dynein function (41). Although extensively studied in other scenarios, the role of EB1 in T lymphocyte metabolism is poorly known. The present study shows that EB1 is necessary for proper metabolic response to glucose upon T cell activation, and that this is related to the correct localization of mitochondria at the IS.

The cytoskeleton reorganization is required for proper positioning of organelles fuelling T cell activation such as mitochondria and lysosomes, and this is directly related to metabolic constraints during adoption of asymmetric fates due to early translation after TCR activation (17, 25). Translation of pre-existing mRNA upon TCR activation in naïve CD4^+^ T cells has been observed for longer times of activation (42, 43). Activation changes the proteomes of CD4^+^ and CD8^+^ T cells in a manner dependent on mTOR (mammalian target of rapamycin) complexes, mainly mTOR complex 1: mTORC1. Metabolic regulation was dependent on this complex in naïve and effector T cells, although cell cycle progression was independent of mTORC1 in naïve cells (43). T cell activation triggers protein synthesis in an asymmetric manner because the centrosome, accompanied by the cell synthesis machinery, relocates toward the IS as early as 3–5 min after activation in human CD4^+^ T cells, although extended times are also possible (17). Moreover, protein synthesis is promoted by TCR pathways and depends on mTOR, which induces ribosome translational activity and also activates the folding activity of relevant chaperones such as CCT (25). CCT regulates the cytoskeleton organization through the folding of actin and tubulin (44) and its knock-down prevents correct mitochondrial localization at the IS, as well as proper mTOR activation (17). CCT is also responsible for the folding of several mTORC1 subunits such as mLS8 (45). Indeed, mTORC1 is also present in the mitochondrial fraction of Jurkat T cells and correlates with mitochondrial activity, i.e. high consumption of oxygen, mitochondrial membrane potential and ATP production. Disruption of the mTOR-RAPTOR complexes with rapamycin or iRNA decreased mitochondrial metabolism (46).

In this regard, the diminished expression of EB1 protein implies changes in this cytoskeletal organization, as well as in activation of T cells (11). Although CKIδ deficiency did not change very early T cell signalling (15) nor EB1 knock-down did for early CD3-dependent signalling, it reduced sustained downstream signalling due to defective interaction of CD3^+^ and LAT^+^ vesicles at the IS (11). Mitochondria, which are recruited to the IS (18, 19), localize near the EB1-enriched plus-TIPs of MTs in resting, polarized T cells such as human T lymphoblasts. Thus, whether EB1 KD affected the polarization of mitochondria to the IS in activated T lymphoblasts and in Jurkat E6-1 T cells was relevant. Mitochondria relocated poorly at the IS in these cells and this finding was accompanied by defects in the relocation of the centrosome, as previously described (11, 15). Also, defective F-actin polarization at the IS in EB1 KD cells was observed. The latter effect on actin cytoskeleton can be due to defective EB1-APC interaction, which also regulates the role of ezrin protein and deregulates actin dynamics, linking them to MT dynamics (31, 32). In addition, this can be due to the defective organization of the mitochondria at the IS, which not only depends on the cytoskeleton, but can in turn regulate the activity of the myosin II motor by helping the phosphorylation and activation of the regulatory light chain (18). Therefore, EB1 function is required to allow proper organelle positioning at the IS, such as vesicles (11), and also mitochondria.

Based on these findings, activation-kinetics experiments showed that the metabolic pathway regulating mTORC1 activity through AKT was affected by EB1 KD. AKT activates mTORC1, which activates ribosome activity through phosphorylation of different factors such as S6 ribosomal protein (26, 47). The activation through phosphorylation of all these proteins was decreased in EB1 KD cells, pointing to defects in the correct activation of T cells. This prompted the study of the ability of T cells to use glucose as an energy fuel, since this is one of the main substrates used by these cells (47). OXPHOS is required for activation of naïve T cells instead of aerobic glycolysis; they are interchangeable to fuel T cell proliferation and survival, but only aerobic glycolysis can facilitate full effector status (48). The use of glucose through glycolysis and subsequent OXPHOS at the mitochondria was defective in EB1 KD cells in resting conditions. These cells were unable to respond to TCR and CD28 activation in terms of OXPHOS increase. This effect can be due to decreased mitochondrial mass, since EB1-deficient clones harboured slight decreased mitochondria, as assessed by flow cytometry, although it was not significant. Therefore, the defect in OCR in EB1 deficient cells seems to be dependent on the mitochondria ability to increase their membrane potential, since they exhibit lower maximal respiration, even upon activation. Also, EB1 depletion has been previously associated with apoptosis of tumour cells via mitochondrial dysfunction and reactive oxygen species (ROS) production (49). Furthermore, EB1-association with MTs was characterized to depend on specific EB1 phosphorylation, which was under the control of ROS and depend on AKT/GSK3β (50). Therefore, the defect in AKT activation and the subsequently defect in mTOR activation and in TCR-induced protein translation can be a more relevant cause for this effect, which merits future investigation. Although the axis PI3K/PTEN could regulate this AKT defect, it is probably not the main cause, since Jurkat E6-1 T cell line is defective in PTEN expression (47). In addition, there were more cells in early and late apoptosis upon activation in EB1 KD Jurkat E6-1 T cells. Hence, the observed changes in apoptosis and mitochondrial functions in EB1-KD T cells might mirror these previous findings and deserve further research. In addition, the observed defect in the activation of the pro-survival kinase AKT can be linked to the observed increment of apoptosis. Also, EB1 deficient cells do not use anaerobic glycolysis to overcome the observed defect in OXPHOS; this effect may be caused by lower mTORC1 activation and subsequent decreased glycolysis activation (47). This is relevant since EB1 knocked-down T lymphoblasts do not increase their anaerobic glycolysis or glycolytic capacity in response to early TCR and CD28 stimulation, which corresponds to mTOR-decreased phosphorylation. These results suggest that these cells could use alternative substrates to glucose to respond to T cell activation. The fact that the blockade of FasL/Fas interaction with an anti-Fas antagonistic antibody clone DX2 inhibited TCR/CD28-controlled AICD but also the pro-apoptotic effect of decreased EB1 expression and the agonistic anti-Fas CH11-induced cell death, demonstrates the contribution of EB1 to FasL/Fas system and the observed AICD. A limitation of this study is that it remains unclear whether the effect of EB1 interference on AICD involves the up-regulation of FasL expression or the enhancement of anti-Fas signalling. Also, both processes can be altered in these cells. Further research is required to establish this point. Altogether, these data support the role of EB1 in the organization of the T cell asymmetry but also T cell apoptosis, connecting the reorganization of the tubulin cytoskeleton and the intracellular organelles with the metabolic regulation of the T cell and warrants future research.

## Materials and methods

### Antibodies and reagents

Antibodies used included pS2448 mTOR, mTOR, pS235-S236 S6, S6 ribosomal subunit, pS473 Akt (Cell Signaling Technology). The rabbit anti-CD3ζ 448 antibody was produced in the laboratory of Dr. B. Alarcón (Centro de Biología Molecular “Severo Ochoa” (CBMSO), Madrid, Spain). Anti-α-Tubulin (clone DM1A, AB_477593), anti-α-Tubulin FITC-conjugated (clone DM1A, AB_476968) and anti-β-Actin (clone AC-15, AB_476692) from Sigma Aldrich; anti-CD3ϵ (clone HIT3a) from BioLegend; αCD3/αCD28 tetramers (Immunocult activator) were from StemCell Technologies; anti-CD28 (clone CD28.2), anti-P150-Glued (1/p150Glued (RUO), AB_397846), anti-CD3ϵ-APC and anti-CD4-APC from BD Biosciences. Isotype control–FITC and isotype control-APC were from Immunostep. Reagents and probes are as follows: MitoTracker™ Orange CMTMRos, CellTracker™ Blue CMAC Dye and Phalloidin Alexa Fluor™ 647 from LifeTechnologies (Invitrogen). Nonyl-acridine-orange was from SigmaAldrich.

### Cells and plasmids

For isolation of CD4^+^ T lymphoblast cultures, human peripheral blood mononuclear cells (PBMCs) were isolated from buffy coats from healthy donors provided by “Centro de Transfusiones de la Comunidad de Madrid” under an agreement with the Hospital Princesa (Madrid) and approved by the CEIm of Hospital Princesa, according to government ethical consent. Upon separation on a Biocoll gradient (Biochrom, L6115), nonadherent cells were collected after plating PBMCs at 37°C and purified using Stem Cell Technologies EasySep kit for CD4^+^ T cells. Cells were then cultured for 48 h in the presence of SEE (0.01 μg/ml; Toxin Technology) and PHA (0.2 μg/ml phytohaemagglutinin, Sigma Aldrich) to induce lymphocyte proliferation, and IL-2 (50 U/ml) was added to the culture medium every 2 days. HuCD4^+^ T lymphoblasts were transfected 7 days after isolation. Experiments were performed 48 h post-transfection.

Jurkat E6-1 cell line (Vαl.2 Vβ8^+^ TCR) was obtained through the NIH AIDS Reagent Program, Division of AIDS, NIAID, NIH from Dr. A. Weiss. Jurkat E6-1 and Raji lymphoblastoid B cell line were grown in RPMI 1640 medium (Gibco-invitrogen) supplemented with 10% fetal bovine serum (FBS, Invitrogen). Cells were cultured at 37 °C, in 5% CO_2_ atmosphere. Jurkat E6-1 T cell clones were cultivated with 0.5 mg/mL^-1^ G418.

### Transfection

Prior to electroporation, Jurkat E6-1 cells or primary CD4^+^ T lymphoblasts were washed first with HBSS and secondly with Opti-MEM I (Gibco, Invitrogen), resuspended in Optimem and 15x10^6^ cells were electroporated with Gene Pulser- II (Bio-Rad) at 240 mV and 975 mA in 4 mm cuvettes containing 400 µL (Bio-Rad). Cells were transfected with a shRNA plasmid encoding a specific 21 bp sequence against EB1 (GACATGACATGCTGGCCTGCG) or with the corresponding control plasmid encoding a negative sequence (TGGCATTGTCTTACCGCCTAT) (Genescript, Piscataway, NJ, USA), or instead with a double-stranded control siRNA or a specific sequence against EB1 (CAGACAAGGUCAAGAAACU and CGUACGCGGAAUACUUCGA, respectively; Eurogentec, San Diego, CA, USA) at a final concentration of 2.5 μM per sample. Dead cells were discarded using Biocoll Separating Solution 24 h post-transfection. Silencing was effective 48 h post-transfection.

### Cell conjugate formation

JK E6-1 cells or CD4^+^ T lymphoblast were activated with SEE-loaded Raji B cells (0.5 μg/mL for 30 min at 37°C; Toxin Technologies, PE404) for the indicated times. For microscopy and Western blot, a 1:1 or 10:1 T:B cell ratio were used, respectively. Cells were centrifuged at low speed to favour earlier proximity between cells and incubated at 37°C for corresponding times. Co-cultures without SEE were prepared in parallel as controls.

### Western blot

For T cell activation, αCD3αCD28 (20 μL/mL ImmunoCult™ Human CD3/CD28 T Cell Activator; StemCell Technologies) or SEE-preloaded Raji cells were used for the times indicated. For SDS-PAGE, 10^6^ cells were lysed in 50 μL 20 mM Tris-HCl (pH 7.5) containing 1% NP40, 0.2% Triton X-100, 150 mM NaCl, 2 mM EDTA, 1.5 mM MgCl_2_ with phosphatase and protease inhibitors. Samples were processed for electrophoresis, transferred to nitrocellulose membranes and subjected to western blot. Primary antibodies were incubated overnight and with peroxidase-labelled secondary antibodies for 1 h. Signal detection was performed using a chemiluminescence imaging system Amersham 880 (51, 52).

### Microscopy

Samples were processed as described (17, 51–53). In brief, Raji cells were stained with the CMAC cell tracker (10 μM; Molecular Probes, C2110) and pulsed with SEE (0.5μg/mL; Toxin Technologies, PE404) for 30 min at 37°C. T cells were stained when indicated with Mitotracker Orange probe (100 nM Molecular Probes) for 30 min at 37°C. T cells. Cell conjugates were adhered to poly-L-Lys-coated coverslips, fixed with 2% paraformaldehyde in PHEM (PIPES 30 mM, Hepes 20 mM, EDTA 2 mM, MgCl_2_ 1 mM, pH: 6.9) containing 0.12 M sucrose for 10 min (R/T), permeabilized with TX-100 (0.2%) for 5 min at R/T and blocked with PHEM containing 100 μg/mL γ-globulin, 3% BSA, 0.2% azide in PHEM for 30 min at R/T. Cells were sequentially stained with the indicated primary antibodies (0.1-5 μg/ml^-1^) followed by Alexa Fluor 488-, 568- or 647-labelled secondary antibodies or Alexa-conjugated phalloidin (5 μg/ml^-1^) or fluorescein isothiocyanate (FITC)-conjugated anti-α-tubulin (0.1 μg/ml^-1^). Samples were mounted on Prolong gold (Invitrogen). A series of fluorescence and brightfield frames were captured using a TCS SP5 confocal laser scanning unit (Leica Microsystems) attached to an inverted epifluorescence microscope (DMI6000) fitted with an HCX PL APO 63x/1.40-0.6 oil objective. Images were acquired and processed with the accompanying confocal software (LCS; Leica) and assembled using Image J software (http://rsbweb.nih.gov/ij/). Polarization of F-actin and mitochondria to the IS was calculated using the the Fiji plugin “Synapse measure” (http://rsbweb.nih.gov/ij/). The distance of the centrosome to the IS was calculated using IMARIS 8.4 software (https://imaris.oxinst.com) as described (51). Imaris software was used to perform 3D reconstruction and volume rendering in **Figure 1C** thanks to its volume tool. The calculations of distances from EB1 plus tips to mitochondria was performed by establishing the centre of mass of each EB1 volume and establishing a threshold of 0.5 μm from the centre of the spot representing it to the mitochondria surface. Probabilistical frequency was calculated from 10 cells.

### Densitometry analysis and quantification of Western blot

Bands from Western blots were analyzed with accompanying software Image Gauge (Fujifilm Inc). Background was subtracted, and arbitrary unit per pixel was normalized to control samples, not stimulated. Values were analyzed with GraphPad for significance.

### Flow cytometry

Approximately, 10^5^ cells of each cellular type were employed in each flow cytometry staining. The primary and secondary antibody staining was maintained for 30 minutes on ice and then washed with FACS buffer (HBSS, human γ-globulin (50 µg/mL), 2% BSA, 1 mM EDTA). Finally, cells were resuspended in 200 µl of FACS buffer for flow cytometry acquisition. Gating strategies are showed in **Supplementary Figures 1**, **2**, **4** and **5**. Data were acquired in a FACSCanto II Analyser Cytometer (405 nm violet laser, 488 nm solid state blue laser and 633 nm He-Ne) (BD

### Activation-induced cell death assay

To assess cell viability 10^5^ cells were incubated with Fas/FasL interaction blocker anti-human Fas mAb (DX2) at 1µg/ml for 1 h, followed by activation in a plate coated with αCD3 (clone HIT3a) (BioLegend) at 10 µg/ml and αCD28 (clone CD28.2) (BD Biosciences) at 3.33 µg/ml (28). One hour after activation, the apoptosis inducer anti-Fas (clone CH-11) monoclonal Ab (mAb) (Sigma Aldrich) was added at 20 ng/ml. After overnight incubation cells were stained with 0,1 µl Ghost dye Red 780 Viability Dye (Tonbo biosciences) in 100 µl PBS during 30 min at 4°C and with 2 µl APC-conjugated annexin V (No. 640920 Biolegend) in 100 µl Annexin V Binding Buffer (No. 422201, Biolegend) for 15 min at RT, following the manufacturer’s instructions. Data were acquired in a FACSCanto II Analyser Cytometer (405 nm violet laser, 488 nm solid state blue laser and 633 nm He-Ne) (BD Biosciences) and analysed with FlowJo software v10.8.1 (BD Biosciences). The gating strategy is included in **Supplementary Figure 5**.

### Seahorse

Experiments were performed as in (17). In brief, XF96 extracellular flux analyser (Seahorse Bioscience; XF96 FluxPak Agilent Technologies) was used for OCR and ECAR measurements. Glucose-based mitostress test was measured in human primary T lymphoblasts or Jurkat E6-1 cells cultured with DMEM medium (D5030, Sigma Aldrich) supplemented with 2 mM sodium pyruvate, 2 mM L-glutamine and 25 mM glucose and drugs injected as follows: oligomycin (2 µM), CCCP (2.5 µM), rotenone plus antimycin A (1.5 µM each). For glycolysis stress test, cells were cultured with DMEM medium supplemented with 3.5 mM L-glutamine. Glucose (10 mM) was injected, followed by oligomycin (1.5 µM) and 2-deoxyglucose (2-DG, 50 mM). A linear mixed model performed in R v4.2.1 (https://cran.r-project.org) was used for statistical analysis (17).

### Statistical analysis

Statistical analyses were performed with PRISM8 (GraphPad software, USA). Normality tests were performed and when comparing two samples, Student T test or Mann-Whitney test were performed, according to normality or not. When comparing three or more samples, two-way ANOVA was used. Specific details of each analysis are detailed in the figure legends. Significant differences were considered when p<0.05 (*). ** indicates p<0.01, *** p<0.001 and **** p<0.0001.

## Data availability statement

The original contributions presented in the study are included in the article/**Supplementary Material**. Further inquiries can be directed to the corresponding author.

## Author contributions

ÁG-M, experimental design, data curation (molecular and cell biology and transfection/nucleofection, Seahorse assays, Western blot and microscopy), image composition, writing (original draft, review and editing), **Figures 1**-**7**, **Supplementary Figures 1**-**5**; SR, experimental design and data curation (molecular and cell biology and transfection/nucleofection, microscopy, Western blot, apoptosis assays), image composition, writing (original draft, review and editing), **Figures 1**-**7**, **Supplementary Figures 1**-**6**; CP, flow cytometry; DC-F, performed experiments **Figure 6**; CS, and IS-G, microscopy, flow cytometry, **Supplementary Figures 3**-**5**; ML-P, Seahorse statistical analysis **Figures 4** and **5**; MI contributed valuable reagents, designed experiments and wrote revised draft; NM-C planned and coordinated research, conceptualization, resources, funding acquisition, data curation, analysed and interpreted data, and wrote the manuscript with input from all authors. All authors contributed to the article and approved the submitted version.

## Supplementary material

Supplementary file Linear mixed model analysis for Seahorse experiments.

Supplementary Figure 1 Gating strategy used to assess purity of CD4+ T lymphoblasts in terms of CD3 and CD4 expression. Corresponds to **Figures 1**–**7**.

Supplementary Figure 2 Expression of receptors in shCtrl and shEB1 Jurkat E6-1 T cell clones. **(A)** Gating strategy. **(B)** Graph, geometric mean of the fluorescence intensity (GeoMFI) of indicated surface markers of shCtrl and shEB1 Jurkat T cells. **(C)** Western blot showing EB1 silencing in shEB1 Jurkat E6-1 T cells. α-tubulin and p150^Glued^ were used as loading controls. **(D)** Western blot showing EB1 silencing in siEB1 Jurkat E6-1 T cells. α-tubulin and p150^Glued^ were used as loading controls. Corresponds to **Figure 3**.

Supplementary Figure 3 Confocal images of cell conjugates formed between shCtrl and shEB1 **(A)** huCD4^+^ T lymphoblasts and **(B)** Jurkat E6-1 T cells with unloaded Raji cells (APCs). Red, mitochondria; green, α-tubulin; magenta, F-actin; blue, CMAC (Raji cell). Maximal projections are shown. Bar, 10 μm. Corresponds to **Figure 3**.

Supplementary Figure 4 Gating strategy used to measure mitochondrial mass with Nonyl acridine orange (NAO). Corresponds to **Figure 6**.

Supplementary Figure 5 Gating strategy used for cell viability analysis. **(A)** Example of the gating strategy for non-activated control (siCtrl; upper panel) and EB1-silenced cells (siEB1; bottom panel) by siRNAs. The left panels show the whole population selected for the cell viability analysis, which included dead and live cells. In the central panel doublets were excluded. In the right panels, the dot plots show the staining of APC-labelled Annexin V and Ghost dye red 780. Annexin^-^ Ghost dye^+^ (Q1) represent necrotic cells, Annexin^+^ Ghost dye^+^ (Q2) are late apoptotic cells, Annexin^+^ Ghost dye^-^ (Q3) indicates early apoptosis and Annexin^-^ Ghost dye^-^ (Q1) are live cells. **(B)** Dot plots of Annexin and Ghost Dye red 780 in siCtrl and siEB1 cells after overnight incubation with αCD3 and αCD28. **(C)** Dot plots of Annexin and Ghost Dye red 780 in siCtrl and siEB1 cells after overnight incubation with anti-Fas antagonistic antibody DX2 and activation with αCD3 and αCD28. **(D)** Dot plots of Annexin and Ghost Dye red 780 in siCtrl and siEB1 cells after overnight incubation with DX2, activation with αCD3 and αCD28 and the apoptotic inducer CH-11. **(E)** Dot plots of Annexin and Ghost Dye red 780 in siCtrl and siEB1 cells after overnight incubation with αCD3, αCD28 and the apoptotic inducer CH-11. Corresponds to **Figure 7**.

Supplementary Figure 6 Cell viability statistical analysis in JK E6-1 T cells. siCtrl; is shown in orange and EB-1 silenced (siEB1) is shown in blue. Non-stimulated cells (-); TCR activation with αCD3 and co-stimulation with αCD28 (CD3/CD28); CD3/CD28 activation plus anti-Fas antagonistic antibody, DX2 (CD3/CD28+DX2); CD3/CD28 activation plus DX2 and apoptotic inducer, CH11 (CD3/CD28+DX2+CH11), or CD3/CD28 activation plus CH-11 (CD3/CD28+CH-11). Graph shows **(A)** Percentage of live cells. **(B)** Percentage of cells in early apoptosis. **(C)** Percentage of cells in late apoptosis. **(D)** Percentage of necrotic cells. Data are mean ± SD, n=3. two-way ANOVA test. *, p<0.05; **, p<0.01; *** p<0.001; **** p<0.0001; ns, non-significant. Corresponds to **Figure 7**.

## References

[1] Nakamura M Zhou XZ Lu KP. Critical role for the EB1 and APC interaction in the regulation of microtubule polymerization. Curr Biol (2001) 11:1062–7. doi: 10.1016/S0960-9822(01)00297-4 11470413

[2] Lawrence EJ Arpag˘ G Norris SR Zanic M. Human CLASP2 specifically regulates microtubule catastrophe and rescue. Mol Biol Cell (2018) 29:1168–77. doi: 10.1091/mbc.E18-01-0016 PMC5935067 29540526

[3] Lansbergen G Akhmanova A. Microtubule plus end: a hub of cellular activities: microtubule plus-End-Tracking proteins. Traffic (2006) 7:499–507. doi: 10.1111/j.1600-0854.2006.00400.x 16643273

[4] Morris EJ Nader GPF Ramalingam N Bartolini F Gundersen GG. Kif4 interacts with EB1 and stabilizes microtubules downstream of rho-mDia in migrating fibroblasts. PloS One (2014) 9:e91568. doi: 10.1371/journal.pone.0091568 24658398 PMC3962350

[5] Thomas GE Bandopadhyay K Sutradhar S Renjith MR Singh P Gireesh KK. EB1 regulates attachment of Ska1 with microtubules by forming extended structures on the microtubule lattice. Nat Commun (2016) 7:11665. doi: 10.1038/ncomms11665 27225956 PMC4894954

[6] Radhakrishnan RM Kizhakkeduth ST Nair VM Ayyappan S Lakshmi RB Babu N. Kinetochore-microtubule attachment in human cells is regulated by the interaction of a conserved motif of Ska1 with EB1. J Biol Chem (2023) 299:102853. doi: 10.1016/j.jbc.2022.102853 36592928 PMC9926122

[7] Temburni MK Rosenberg MM Pathak N McConnell R Jacob MH. Neuronal nicotinic synapse assembly requires the adenomatous polyposis coli tumor suppressor protein. J Neurosci (2004) 24:6776–84. doi: 10.1523/JNEUROSCI.1826-04.2004 PMC6729726 15282282

[8] Weiss A Littman DR. Signal transduction by lymphocyte antigen receptors. Cell (1994) 76:263–74. doi: 10.1016/0092-8674(94)90334-4 8293463

[9] Palacios EH Weiss A. Function of the Src-family kinases, Lck and Fyn, in T-cell development and activation. Oncogene (2004) 23:7990–8000. doi: 10.1038/sj.onc.1208074 15489916

[10] Monks CRF Freiberg BA Kupfer H Sciaky N Kupfer A. Three-dimensional segregation of supramolecular activation clusters in T cells. Nature (1998) 395:82–6. doi: 10.1038/25764 9738502

[11] Martín-Cófreces NB Baixauli F López MJ Gil D Monjas A Alarcón B. End-binding protein 1 controls signal propagation from the T cell receptor: EB1 regulates TCR signalling. EMBO J (2012) 31:4140–52. doi: 10.1038/emboj.2012.242 PMC3492726 22922463

[12] Maccari I Zhao R Peglow M Schwarz K Hornak I Pasche M. Cytoskeleton rotation relocates mitochondria to the immunological synapse and increases calcium signals. Cell Calcium (2016) 60:309–21. doi: 10.1016/j.ceca.2016.06.007 27451384

[13] Quann EJ Merino E Furuta T Huse M. Localized diacylglycerol drives the polarization of the microtubule-organizing center in T cells. Nat Immunol (2009) 10:627–35. doi: 10.1038/ni.1734 19430478

[14] Filbert EL Le Borgne M Lin J Heuser JE Shaw AS. Stathmin regulates microtubule dynamics and microtubule organizing center polarization in activated T cells. J Immunol (2012) 188:5421–7. doi: 10.4049/jimmunol.1200242 PMC3358357 22529300

[15] Zyss D Ebrahimi H Gergely F. Casein kinase I delta controls centrosome positioning during T cell activation. J Cell Biol (2011) 195:781–97. doi: 10.1083/jcb.201106025 PMC3257584 22123863

[16] Hooikaas PJ Damstra HG Gros OJ van Riel WE Martin M Smits YT. Kinesin-4 KIF21B limits microtubule growth to allow rapid centrosome polarization in T cells. eLife (2020) 9:e62876. doi: 10.7554/eLife.62876 33346730 PMC7817182

[17] Martin-Cofreces NB Chichon FJ Calvo E Torralba D Bustos-Moran E Dosil SG. The chaperonin CCT controls T cell receptor–driven 3D configuration of centrioles. Sci Adv (2020) 6:eabb7242. doi: 10.1126/sciadv.abb7242 33268369 PMC7821906

[18] Baixauli F Martín-Cófreces NB Morlino G Carrasco YR Calabia-Linares C Veiga E. The mitochondrial fission factor dynamin-related protein 1 modulates T-cell receptor signalling at the immune synapse: Drp1 and T-cell activation. EMBO J (2011) 30:1238–50. doi: 10.1038/emboj.2011.25 PMC3094108 21326213

[19] Quintana A Schwindling C Wenning AS Becherer U Rettig J Schwarz EC. T Cell activation requires mitochondrial translocation to the immunological synapse. Proc Natl Acad Sci USA (2007) 104:14418–23. doi: 10.1073/pnas.0703126104 PMC1964825 17726106

[20] Quintana A Pasche M Junker C Al-Ansary D Rieger H Kummerow C. Calcium microdomains at the immunological synapse: how ORAI channels, mitochondria and calcium pumps generate local calcium signals for efficient T-cell activation: calcium microdomains at the immunological synapse. EMBO J (2011) 30:3895–912. doi: 10.1038/emboj.2011.289 PMC3209779 21847095

[21] Ledderose C Bao Y Lidicky M Zipperle J Li L Strasser K. Mitochondria are gate-keepers of T cell function by producing the ATP that drives purinergic signaling. J Biol Chem (2014) 289:25936–45. doi: 10.1074/jbc.M114.575308 PMC4162192 25070895

[22] Ledderose C Bromberger S Slubowski CJ Sueyoshi K Junger WG. Frontline science: P2Y11 receptors support T cell activation by directing mitochondrial trafficking to the immune synapse. J Leukoc Biol (2021) 109:497–508. doi: 10.1002/JLB.2HI0520-191R 32531829 PMC8772287

[23] Chang C-L Chen Y-J Quintanilla CG Hsieh T-S Liou J. EB1 binding restricts STIM1 translocation to ER–PM junctions and regulates store-operated Ca2+ entry. J Cell Biol (2018) 217:2047–58. doi: 10.1083/jcb.201711151 PMC5987725 29563214

[24] Jambrina E Alonso R Alcalde M del Carmen Rodríguez M Serrano A Martínez-A C. Calcium influx through receptor-operated channel induces mitochondria-triggered paraptotic cell death. J Biol Chem (2003) 278:14134–45. doi: 10.1074/jbc.M211388200 12571238

[25] Martin-Cofreces NB Valpuesta JM Sánchez-Madrid F. T Cell asymmetry and metabolic crosstalk can fine-tune immunological synapses. Trends Immunol (2021) 42:649–53. doi: 10.1016/j.it.2021.06.007 34226146

[26] Chi H. Regulation and function of mTOR signalling in T cell fate decisions. Nat Rev Immunol (2012) 12:325–38. doi: 10.1038/nri3198 PMC3417069 22517423

[27] Hwang J-R Byeon Y Kim D Park S-G. Recent insights of T cell receptor-mediated signaling pathways for T cell activation and development. Exp Mol Med (2020) 52:750–61. doi: 10.1038/s12276-020-0435-8 PMC7272404 32439954

[28] Alonso R Rodríguez MC Pindado J Merino E Mérida I Izquierdo M. Diacylglycerol kinase α regulates the secretion of lethal exosomes bearing fas ligand during activation-induced cell death of T lymphocytes. J Biol Chem (2005) 280:28439–50. doi: 10.1074/jbc.M501112200 15870081

[29] Su LK Burrell M Hill DE Gyuris J Brent R Wiltshire R. APC binds to the novel protein EB1. Cancer Res (1995) 55:2972–7. 7606712

[30] Etienne-Manneville S Hall A. Cdc42 regulates GSK-3beta and adenomatous polyposis coli to control cell polarity. Nature (2003) 421:753–6. doi: 10.1038/nature01423 12610628

[31] Agüera-González S Burton OT Vázquez-Chávez E Cuche C Herit F Bouchet J. Adenomatous polyposis coli defines treg differentiation and anti-inflammatory function through microtubule-mediated NFAT localization. Cell Rep (2017) 21:181–94. doi: 10.1016/j.celrep.2017.09.020 28978472

[32] Lasserre R Charrin S Cuche C Danckaert A Thoulouze M-I de Chaumont F. Ezrin tunes T-cell activation by controlling Dlg1 and microtubule positioning at the immunological synapse. EMBO J (2010) 29:2301–14. doi: 10.1038/emboj.2010.127 PMC2910277 20551903

[33] Martín-Cófreces NB Robles-Valero J Cabrero JR Mittelbrunn M Gordón-Alonso M Sung C-H. MTOC translocation modulates IS formation and controls sustained T cell signaling. J Cell Biol (2008) 182:951–62. doi: 10.1083/jcb.200801014 PMC2528574 18779373

[34] Chan DC. Mitochondria: dynamic organelles in disease, aging, and development. Cell (2006) 125:1241–52. doi: 10.1016/j.cell.2006.06.010 16814712

[35] Suen D-F Norris KL Youle RJ. Mitochondrial dynamics and apoptosis. Genes Dev (2008) 22:1577–90. doi: 10.1101/gad.1658508 PMC2732420 18559474

[36] Hirokawa N Takemura R. Molecular motors and mechanisms of directional transport in neurons. Nat Rev Neurosci (2005) 6:201–14. doi: 10.1038/nrn1624 15711600

[37] Schwarz TL. Mitochondrial trafficking in neurons. Cold Spring Harb Perspect Biol (2013) 5:a011304–a011304. doi: 10.1101/cshperspect.a011304 23732472 PMC3660831

[38] van Spronsen M Mikhaylova M Lipka J Schlager MA van den Heuvel DJ Kuijpers M. TRAK/Milton motor-adaptor proteins steer mitochondrial trafficking to axons and dendrites. Neuron (2013) 77:485–502. doi: 10.1016/j.neuron.2012.11.027 23395375

[39] Kruppa AJ Buss F. Motor proteins at the mitochondria–cytoskeleton interface. J Cell Sci (2021) 134:jcs226084. doi: 10.1242/jcs.226084 33912943 PMC8077471

[40] Rintoul GL Filiano AJ Brocard JB Kress GJ Reynolds IJ. Glutamate decreases mitochondrial size and movement in primary forebrain neurons. J Neurosci (2003) 23:7881–8. doi: 10.1523/JNEUROSCI.23-21-07881.2003 PMC6740596 12944518

[41] Morlino G Barreiro O Baixauli F Robles-Valero J González-Granado JM Villa-Bellosta R. Miro-1 links mitochondria and microtubule Dynein motors to control lymphocyte migration and polarity. Mol Cell Biol (2014) 34:1412–26. doi: 10.1128/MCB.01177-13 PMC3993592 24492963

[42] Wolf T Jin W Zoppi G Vogel IA Akhmedov M Bleck CKE. Dynamics in protein translation sustaining T cell preparedness. Nat Immunol (2020) 21:927–37. doi: 10.1038/s41590-020-0714-5 PMC7610365 32632289

[43] Howden AJM Hukelmann JL Brenes A Spinelli L Sinclair LV Lamond AI. Quantitative analysis of T cell proteomes and environmental sensors during T cell differentiation. Nat Immunol (2019) 20:1542–54. doi: 10.1038/s41590-019-0495-x PMC6859072 31591570

[44] Willison KR. The substrate specificity of eukaryotic cytosolic chaperonin CCT. Phil Trans R Soc B (2018) 373:20170192. doi: 10.1098/rstb.2017.0192 29735743 PMC5941184

[45] Cuéllar J Ludlam WG Tensmeyer NC Aoba T Dhavale M Santiago C. Structural and functional analysis of the role of the chaperonin CCT in mTOR complex assembly. Nat Commun (2019) 10:2865. doi: 10.1038/s41467-019-10781-1 31253771 PMC6599039

[46] Schieke SM Phillips D McCoy JP Aponte AM Shen R-F Balaban RS. The mammalian target of rapamycin (mTOR) pathway regulates mitochondrial oxygen consumption and oxidative capacity. J Biol Chem (2006) 281:27643–52. doi: 10.1074/jbc.M603536200 16847060

[47] Shyer JA Flavell RA Bailis W. Metabolic signaling in T cells. Cell Res (2020) 30:649–59. doi: 10.1038/s41422-020-0379-5 PMC7395146 32709897

[48] Chang C-H Curtis JD Maggi LB Faubert B Villarino AV O’Sullivan D. Posttranscriptional control of T cell effector function by aerobic glycolysis. Cell (2013) 153:1239–51. doi: 10.1016/j.cell.2013.05.016 PMC3804311 23746840

[49] Kim M-J Yun HS Hong E-H Lee S-J Baek J-H Lee C-W. Depletion of end-binding protein 1 (EB1) promotes apoptosis of human non-small-cell lung cancer cells via reactive oxygen species and bax-mediated mitochondrial dysfunction. Cancer Lett (2013) 339:15–24. doi: 10.1016/j.canlet.2013.07.027 23900080

[50] Grand ML Rovini A Bourgarel-Rey V Honore S Bastonero S Braguer D. ROS-mediated EB1 phosphorylation through Akt/GSK3β pathway: implication in cancer cell response to microtubule-targeting agents. Oncotarget (2014) 5:3408–23. doi: 10.18632/oncotarget.1982 PMC4102819 24930764

[51] Blas-Rus N Bustos-Morán E Sánchez-Madrid F Martín-Cófreces NB. Analysis of microtubules and microtubule-organizing center at the immune synapse. Methods Mol Biol (2017) 1584:31–49. doi: 10.1007/978-1-4939-6881-7_3 28255694 PMC5503130

[52] Gómez-Morón A Requena S Roda-Navarro P Martín-Cófreces NB. Activation kinetics of regulatory molecules during immunological synapse in T cells. Methods Cell Biol Elsevier (2023). doi: 10.1016/bs.mcb.2022.10.014 37516524

[53] Carrasco-Padilla C Aguilar-Sopeña O Gómez-Morón A Alegre-Gómez S Sánchez-Madrid F Martín-Cófreces NB. T Cell activation and effector function in the human Jurkat T cell model. Methods Cell Biol (2022), S0091679X22001534. doi: 10.1016/bs.mcb.2022.09.012 37516527

