# Supplementary material for: End-binding protein 1 regulates the metabolic fate of CD4+ T lymphoblasts and Jurkat T cells and the organization of the mitochondrial network
Source: Front Immunol. 2023 Jul 13;14:1197289. doi: 10.3389/fimmu.2023.1197289 (PMC10374013; doi:10.3389/fimmu.2023.1197289)
Supplement: Supplementary file — Linear mixed model analysis for Seahorse experiments. [file DataSheet_1.pdf]

Seahorse statistical analysis

Glycolysis Stress OCR - Jurkat EB1 clones

```
library(lattice)
library(AICcmodavg)
library(emmeans)
library(lme4)
```

```
## Loading required package: Matrix

##
## Attaching package: 'lme4'

## The following object is masked from 'package:AICcmodavg':
##
##     checkConv
```

```
library(lmerTest)
```

```
##
## Attaching package: 'lmerTest'

## The following object is masked from 'package:lme4':
##
##     lmer

## The following object is masked from 'package:stats':
##
##     step
```

```
library(lmtest)
```

```
## Loading required package: zoo

##
## Attaching package: 'zoo'

## The following objects are masked from 'package:base':
##
##     as.Date, as.Date.numeric
```

```r
library(ggplot2)
```

```r
ggplot(seahorse1, aes(time, value, colour=condition))+
  geom_point()+
  theme_bw()+
  xlab("Time (min)") +
  ylab('Value')
```

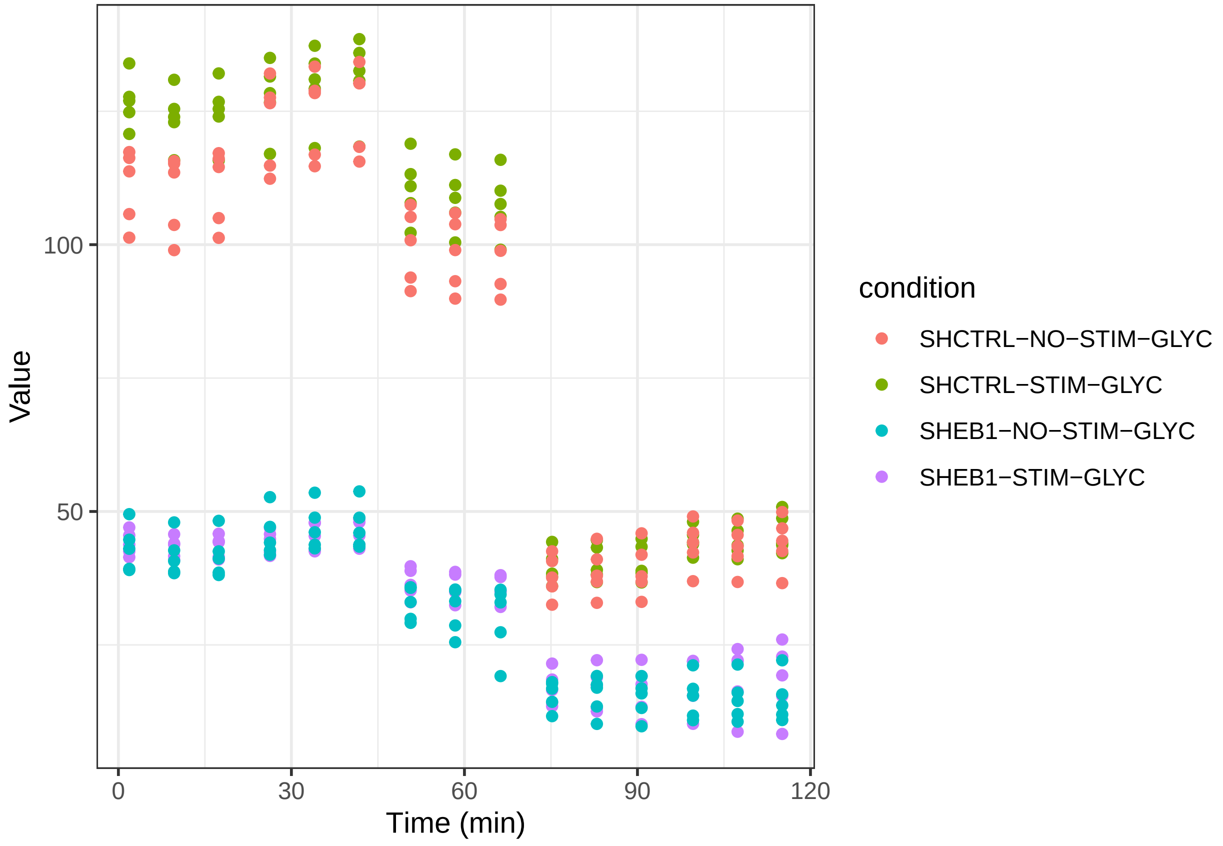


```r
m1<-lm(value ~ treatment*condition, data=seahorse1)
summary(m1)
```

```
##
## Call:
## lm(formula = value ~ treatment * condition, data = seahorse1)
##
## Residuals:
##      Min       1Q   Median       3Q      Max
## -12.6045  -2.9476   0.2799   3.3369   9.9649
##
## Coefficients:
##                                          Estimate Std. Error t value Pr(>|t|)
## (Intercept)                              110.3693     1.2281  89.873  < 2e-16 ***
## treatmentb                                13.9046     1.7367   8.006 3.18e-14 ***
## treatmentc                               -11.6995     1.7367  -6.737 9.20e-11 ***
```

```
## treatmentd                            -71.7978     1.7367 -41.341  < 2e-16 ***
## treatmente                            -66.7226     1.7367 -38.418  < 2e-16 ***
## conditionSHCTRL-STIM-GLYC              14.7876     1.7367   8.515 1.04e-15 ***
## conditionSHEB1-NO-STIM-GLYC           -68.1907     1.7367 -39.264  < 2e-16 ***
## conditionSHEB1-STIM-GLYC              -66.8059     1.7367 -38.466  < 2e-16 ***
## treatmentb:conditionSHCTRL-STIM-GLYC    -9.4439     2.4561  -3.845 0.000149 ***
## treatmentc:conditionSHCTRL-STIM-GLYC    -4.5030     2.4561  -1.833 0.067809 .
## treatmentd:conditionSHCTRL-STIM-GLYC   -13.2418     2.4561  -5.391 1.49e-07 ***
## treatmente:conditionSHCTRL-STIM-GLYC   -13.4276     2.4561  -5.467 1.01e-07 ***
## treatmentb:conditionSHEB1-NO-STIM-GLYC  -9.4319     2.4561  -3.840 0.000152 ***
## treatmentc:conditionSHEB1-NO-STIM-GLYC   0.9006     2.4561   0.367 0.714143
## treatmentd:conditionSHEB1-NO-STIM-GLYC  45.0052     2.4561  18.324  < 2e-16 ***
## treatmente:conditionSHEB1-NO-STIM-GLYC  39.5433     2.4561  16.100  < 2e-16 ***
## treatmentb:conditionSHEB1-STIM-GLYC    -12.3366     2.4561  -5.023 9.08e-07 ***
## treatmentc:conditionSHEB1-STIM-GLYC      4.1682     2.4561   1.697 0.090793 .
## treatmentd:conditionSHEB1-STIM-GLYC     44.9594     2.4561  18.305  < 2e-16 ***
## treatmente:conditionSHEB1-STIM-GLYC     41.5973     2.4561  16.936  < 2e-16 ***
## ---
## Signif. codes:  0 '***' 0.001 '**' 0.01 '*' 0.05 '.' 0.1 ' ' 1
##
## Residual standard error: 4.756 on 280 degrees of freedom
## Multiple R-squared:  0.9865, Adjusted R-squared:  0.9856
## F-statistic:  1081 on 19 and 280 DF,  p-value: < 2.2e-16
```

```
summary(emmeans(m1, pairwise ~ condition|treatment, infer=T))
```

```
## $emmeans
## treatment = a:
##  condition           emmean   SE  df lower.CL upper.CL t.ratio p.value
##  SHCTRL-NO-STIM-GLYC  110.4 1.23 280    108.0    112.8  89.873  <.0001
##  SHCTRL-STIM-GLYC     125.2 1.23 280    122.7    127.6 101.915  <.0001
##  SHEB1-NO-STIM-GLYC    42.2 1.23 280     39.8     44.6  34.346  <.0001
##  SHEB1-STIM-GLYC       43.6 1.23 280     41.1     46.0  35.474  <.0001
##
## treatment = b:
##  condition           emmean   SE  df lower.CL upper.CL t.ratio p.value
##  SHCTRL-NO-STIM-GLYC  124.3 1.23 280    121.9    126.7 101.196  <.0001
##  SHCTRL-STIM-GLYC     129.6 1.23 280    127.2    132.0 105.547  <.0001
##  SHEB1-NO-STIM-GLYC    46.7 1.23 280     44.2     49.1  37.988  <.0001
##  SHEB1-STIM-GLYC       45.1 1.23 280     42.7     47.5  36.750  <.0001
##
## treatment = c:
##  condition           emmean   SE  df lower.CL upper.CL t.ratio p.value
##  SHCTRL-NO-STIM-GLYC   98.7 1.23 280     96.3    101.1  80.346  <.0001
##  SHCTRL-STIM-GLYC     109.0 1.23 280    106.5    111.4  88.721  <.0001
##  SHEB1-NO-STIM-GLYC    31.4 1.23 280     29.0     33.8  25.552  <.0001
##  SHEB1-STIM-GLYC       36.0 1.23 280     33.6     38.4  29.341  <.0001
##
## treatment = d:
##  condition           emmean   SE  df lower.CL upper.CL t.ratio p.value
##  SHCTRL-NO-STIM-GLYC   38.6 1.23 280     36.2     41.0  31.409  <.0001
##  SHCTRL-STIM-GLYC      40.1 1.23 280     37.7     42.5  32.667  <.0001
##  SHEB1-NO-STIM-GLYC    15.4 1.23 280     13.0     17.8  12.529  <.0001
##  SHEB1-STIM-GLYC       16.7 1.23 280     14.3     19.1  13.619  <.0001
```

```
##
## treatment = e:
##  condition           emmean   SE  df lower.CL upper.CL t.ratio p.value
##  SHCTRL-NO-STIM-GLYC   43.6 1.23 280     41.2     46.1  35.541  <.0001
##  SHCTRL-STIM-GLYC      45.0 1.23 280     42.6     47.4  36.649  <.0001
##  SHEB1-NO-STIM-GLYC    15.0 1.23 280     12.6     17.4  12.214  <.0001
##  SHEB1-STIM-GLYC       18.4 1.23 280     16.0     20.9  15.014  <.0001
##
## Confidence level used: 0.95
##
## $contrasts
## treatment = a:
##  contrast                                    estimate   SE  df lower.CL
##  (SHCTRL-NO-STIM-GLYC) - (SHCTRL-STIM-GLYC)    -14.79 1.74 280   -19.28
##  (SHCTRL-NO-STIM-GLYC) - (SHEB1-NO-STIM-GLYC)   68.19 1.74 280    63.70
##  (SHCTRL-NO-STIM-GLYC) - (SHEB1-STIM-GLYC)      66.81 1.74 280    62.32
##  (SHCTRL-STIM-GLYC) - (SHEB1-NO-STIM-GLYC)      82.98 1.74 280    78.49
##  (SHCTRL-STIM-GLYC) - (SHEB1-STIM-GLYC)         81.59 1.74 280    77.10
##  (SHEB1-NO-STIM-GLYC) - (SHEB1-STIM-GLYC)       -1.38 1.74 280    -5.87
##  upper.CL t.ratio p.value
##   -10.299  -8.515  <.0001
##    72.679  39.264  <.0001
##    71.295  38.466  <.0001
##    87.467  47.778  <.0001
##    86.082  46.981  <.0001
##     3.104  -0.797  0.8556
##
## treatment = b:
##  contrast                                    estimate   SE  df lower.CL
##  (SHCTRL-NO-STIM-GLYC) - (SHCTRL-STIM-GLYC)     -5.34 1.74 280    -9.83
##  (SHCTRL-NO-STIM-GLYC) - (SHEB1-NO-STIM-GLYC)   77.62 1.74 280    73.13
##  (SHCTRL-NO-STIM-GLYC) - (SHEB1-STIM-GLYC)      79.14 1.74 280    74.65
##  (SHCTRL-STIM-GLYC) - (SHEB1-NO-STIM-GLYC)      82.97 1.74 280    78.48
##  (SHCTRL-STIM-GLYC) - (SHEB1-STIM-GLYC)         84.49 1.74 280    80.00
##  (SHEB1-NO-STIM-GLYC) - (SHEB1-STIM-GLYC)        1.52 1.74 280    -2.97
##  upper.CL t.ratio p.value
##    -0.855  -3.077  0.0122
##    82.111  44.695  <.0001
##    83.631  45.570  <.0001
##    87.455  47.772  <.0001
##    88.975  48.647  <.0001
##     6.008   0.875  0.8177
##
## treatment = c:
##  contrast                                    estimate   SE  df lower.CL
##  (SHCTRL-NO-STIM-GLYC) - (SHCTRL-STIM-GLYC)    -10.28 1.74 280   -14.77
##  (SHCTRL-NO-STIM-GLYC) - (SHEB1-NO-STIM-GLYC)   67.29 1.74 280    62.80
##  (SHCTRL-NO-STIM-GLYC) - (SHEB1-STIM-GLYC)      62.64 1.74 280    58.15
##  (SHCTRL-STIM-GLYC) - (SHEB1-NO-STIM-GLYC)      77.57 1.74 280    73.09
##  (SHCTRL-STIM-GLYC) - (SHEB1-STIM-GLYC)         72.92 1.74 280    68.43
##  (SHEB1-NO-STIM-GLYC) - (SHEB1-STIM-GLYC)       -4.65 1.74 280    -9.14
##  upper.CL t.ratio p.value
##    -5.796  -5.922  <.0001
##    71.779  38.745  <.0001
```

```
##    67.126  36.066  <.0001
##    82.063  44.667  <.0001
##    77.411  41.988  <.0001
##    -0.164  -2.679  0.0389
##
## treatment = d:
##  contrast                                     estimate   SE  df lower.CL
##  (SHCTRL-NO-STIM-GLYC) - (SHCTRL-STIM-GLYC)      -1.55 1.74 280    -6.03
##  (SHCTRL-NO-STIM-GLYC) - (SHEB1-NO-STIM-GLYC)    23.19 1.74 280    18.70
##  (SHCTRL-NO-STIM-GLYC) - (SHEB1-STIM-GLYC)       21.85 1.74 280    17.36
##  (SHCTRL-STIM-GLYC) - (SHEB1-NO-STIM-GLYC)       24.73 1.74 280    20.24
##  (SHCTRL-STIM-GLYC) - (SHEB1-STIM-GLYC)          23.39 1.74 280    18.90
##  (SHEB1-NO-STIM-GLYC) - (SHEB1-STIM-GLYC)        -1.34 1.74 280    -5.83
##  upper.CL t.ratio p.value
##     2.943  -0.890  0.8100
##    27.674  13.350  <.0001
##    26.335  12.579  <.0001
##    29.220  14.240  <.0001
##    27.881  13.469  <.0001
##     3.150  -0.771  0.8675
##
## treatment = e:
##  contrast                                     estimate   SE  df lower.CL
##  (SHCTRL-NO-STIM-GLYC) - (SHCTRL-STIM-GLYC)      -1.36 1.74 280    -5.85
##  (SHCTRL-NO-STIM-GLYC) - (SHEB1-NO-STIM-GLYC)    28.65 1.74 280    24.16
##  (SHCTRL-NO-STIM-GLYC) - (SHEB1-STIM-GLYC)       25.21 1.74 280    20.72
##  (SHCTRL-STIM-GLYC) - (SHEB1-NO-STIM-GLYC)       30.01 1.74 280    25.52
##  (SHCTRL-STIM-GLYC) - (SHEB1-STIM-GLYC)          26.57 1.74 280    22.08
##  (SHEB1-NO-STIM-GLYC) - (SHEB1-STIM-GLYC)        -3.44 1.74 280    -7.93
##  upper.CL t.ratio p.value
##     3.129  -0.783  0.8621
##    33.136  16.495  <.0001
##    29.697  14.515  <.0001
##    34.496  17.278  <.0001
##    31.057  15.298  <.0001
##     1.050  -1.980  0.1980
##
## Confidence level used: 0.95
## Conf-level adjustment: tukey method for comparing a family of 4 estimates
## P value adjustment: tukey method for comparing a family of 4 estimates
```

```
emmip(m1, condition ~ treatment) +
  theme_bw()+
  xlab('Treatment') +
  ylab('Value')
```

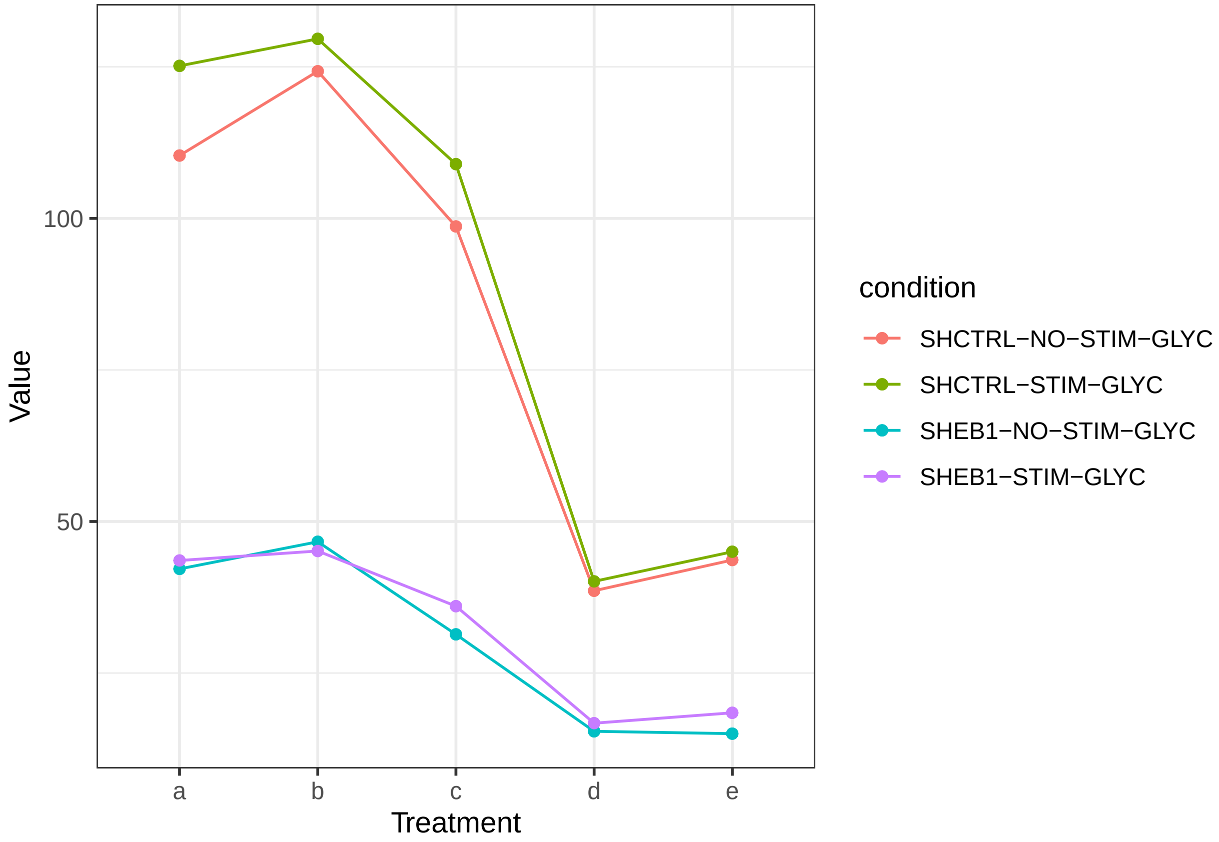


```
summary(pairs(emmeans(m1, "condition",by = "treatment",level = 0.95, infer = TRUE)))
```

```
## treatment = a:
##  contrast                                    estimate   SE  df t.ratio p.value
##  (SHCTRL-NO-STIM-GLYC) - (SHCTRL-STIM-GLYC)    -14.79 1.74 280  -8.515  <.0001
##  (SHCTRL-NO-STIM-GLYC) - (SHEB1-NO-STIM-GLYC)   68.19 1.74 280  39.264  <.0001
##  (SHCTRL-NO-STIM-GLYC) - (SHEB1-STIM-GLYC)      66.81 1.74 280  38.466  <.0001
##  (SHCTRL-STIM-GLYC) - (SHEB1-NO-STIM-GLYC)      82.98 1.74 280  47.778  <.0001
##  (SHCTRL-STIM-GLYC) - (SHEB1-STIM-GLYC)         81.59 1.74 280  46.981  <.0001
##  (SHEB1-NO-STIM-GLYC) - (SHEB1-STIM-GLYC)       -1.38 1.74 280  -0.797  0.8556
##
## treatment = b:
##  contrast                                    estimate   SE  df t.ratio p.value
##  (SHCTRL-NO-STIM-GLYC) - (SHCTRL-STIM-GLYC)     -5.34 1.74 280  -3.077  0.0122
##  (SHCTRL-NO-STIM-GLYC) - (SHEB1-NO-STIM-GLYC)   77.62 1.74 280  44.695  <.0001
##  (SHCTRL-NO-STIM-GLYC) - (SHEB1-STIM-GLYC)      79.14 1.74 280  45.570  <.0001
##  (SHCTRL-STIM-GLYC) - (SHEB1-NO-STIM-GLYC)      82.97 1.74 280  47.772  <.0001
##  (SHCTRL-STIM-GLYC) - (SHEB1-STIM-GLYC)         84.49 1.74 280  48.647  <.0001
##  (SHEB1-NO-STIM-GLYC) - (SHEB1-STIM-GLYC)        1.52 1.74 280   0.875  0.8177
##
## treatment = c:
##  contrast                                    estimate   SE  df t.ratio p.value
##  (SHCTRL-NO-STIM-GLYC) - (SHCTRL-STIM-GLYC)    -10.28 1.74 280  -5.922  <.0001
##  (SHCTRL-NO-STIM-GLYC) - (SHEB1-NO-STIM-GLYC)   67.29 1.74 280  38.745  <.0001
##  (SHCTRL-NO-STIM-GLYC) - (SHEB1-STIM-GLYC)      62.64 1.74 280  36.066  <.0001
```

```
##  (SHCTRL-STIM-GLYC) - (SHEB1-NO-STIM-GLYC)       77.57 1.74 280  44.667  <.0001
##  (SHCTRL-STIM-GLYC) - (SHEB1-STIM-GLYC)          72.92 1.74 280  41.988  <.0001
##  (SHEB1-NO-STIM-GLYC) - (SHEB1-STIM-GLYC)        -4.65 1.74 280  -2.679  0.0389
##
## treatment = d:
##  contrast                                     estimate   SE  df t.ratio p.value
##  (SHCTRL-NO-STIM-GLYC) - (SHCTRL-STIM-GLYC)      -1.55 1.74 280  -0.890  0.8100
##  (SHCTRL-NO-STIM-GLYC) - (SHEB1-NO-STIM-GLYC)    23.19 1.74 280  13.350  <.0001
##  (SHCTRL-NO-STIM-GLYC) - (SHEB1-STIM-GLYC)       21.85 1.74 280  12.579  <.0001
##  (SHCTRL-STIM-GLYC) - (SHEB1-NO-STIM-GLYC)       24.73 1.74 280  14.240  <.0001
##  (SHCTRL-STIM-GLYC) - (SHEB1-STIM-GLYC)          23.39 1.74 280  13.469  <.0001
##  (SHEB1-NO-STIM-GLYC) - (SHEB1-STIM-GLYC)        -1.34 1.74 280  -0.771  0.8675
##
## treatment = e:
##  contrast                                     estimate   SE  df t.ratio p.value
##  (SHCTRL-NO-STIM-GLYC) - (SHCTRL-STIM-GLYC)      -1.36 1.74 280  -0.783  0.8621
##  (SHCTRL-NO-STIM-GLYC) - (SHEB1-NO-STIM-GLYC)    28.65 1.74 280  16.495  <.0001
##  (SHCTRL-NO-STIM-GLYC) - (SHEB1-STIM-GLYC)       25.21 1.74 280  14.515  <.0001
##  (SHCTRL-STIM-GLYC) - (SHEB1-NO-STIM-GLYC)       30.01 1.74 280  17.278  <.0001
##  (SHCTRL-STIM-GLYC) - (SHEB1-STIM-GLYC)          26.57 1.74 280  15.298  <.0001
##  (SHEB1-NO-STIM-GLYC) - (SHEB1-STIM-GLYC)        -3.44 1.74 280  -1.980  0.1980
##
## P value adjustment: tukey method for comparing a family of 4 estimates
```

Glycolysis Stress ECAR - Jurkat EB1 clones

```
ggplot(seahorse2, aes(time, value, colour=condition))+
  geom_point()+
  theme_bw()+
  xlab("Time (min)") +
  ylab('Value')
```

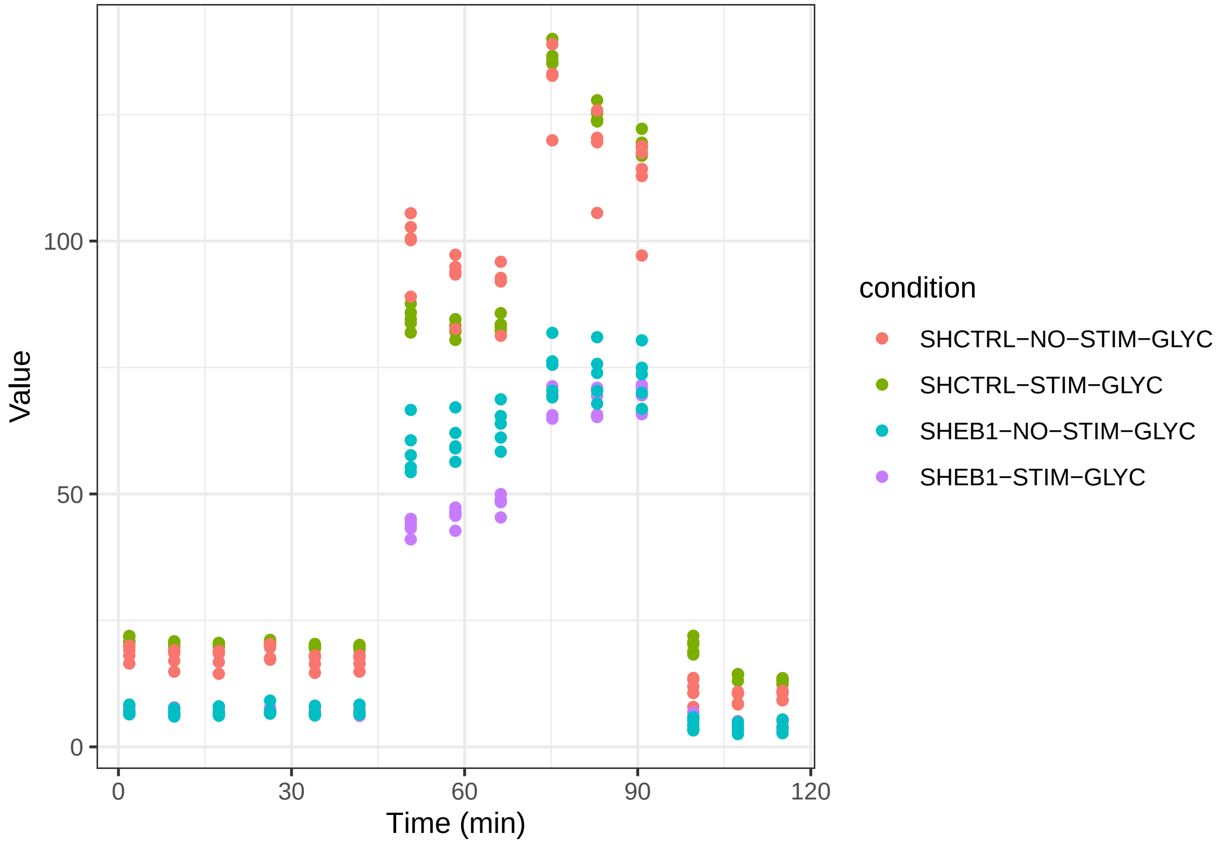


```
m2<-lm(value ~ treatment*condition, data=seahorse2)
summary(m2)
```

```
##
## Call:
## lm(formula = value ~ treatment * condition, data = seahorse2)
##
## Residuals:
##      Min       1Q   Median       3Q      Max
## -23.4930  -1.1957  -0.0878   0.9518  18.2661
##
## Coefficients:
##                                      Estimate Std. Error t value Pr(>|t|)
## (Intercept)                           17.9608     1.0141  17.712  < 2e-16 ***
## treatmentb                            -0.3064     1.4341  -0.214   0.8310
## treatmentc                            76.3316     1.4341  53.226  < 2e-16 ***
## treatmentd                           102.6775     1.4341  71.597  < 2e-16 ***
## treatmente                            -7.4720     1.4341  -5.210 3.66e-07 ***
## conditionSHCTRL-STIM-GLYC              2.6646     1.4341   1.858   0.0642 .
## conditionSHEB1-NO-STIM-GLYC          -11.1531     1.4341  -7.777 1.43e-13 ***
## conditionSHEB1-STIM-GLYC             -10.7562     1.4341  -7.500 8.46e-13 ***
## treatmentb:conditionSHCTRL-STIM-GLYC  -0.2191     2.0281  -0.108   0.9140
## treatmentc:conditionSHCTRL-STIM-GLYC -13.3361     2.0281  -6.576 2.37e-10 ***
## treatmentd:conditionSHCTRL-STIM-GLYC   3.5458     2.0281   1.748   0.0815 .
## treatmente:conditionSHCTRL-STIM-GLYC   2.5908     2.0281   1.277   0.2025
```

```
## treatmentb:conditionSHEB1-NO-STIM-GLYC   0.5263     2.0281   0.259   0.7955
## treatmentc:conditionSHEB1-NO-STIM-GLYC -22.0758     2.0281 -10.885  < 2e-16 ***
## treatmentd:conditionSHEB1-NO-STIM-GLYC -35.6315     2.0281 -17.569  < 2e-16 ***
## treatmente:conditionSHEB1-NO-STIM-GLYC   4.6693     2.0281   2.302   0.0221 *
## treatmentb:conditionSHEB1-STIM-GLYC      0.2260     2.0281   0.111   0.9113
## treatmentc:conditionSHEB1-STIM-GLYC    -37.7384     2.0281 -18.608  < 2e-16 ***
## treatmentd:conditionSHEB1-STIM-GLYC    -41.4123     2.0281 -20.419  < 2e-16 ***
## treatmente:conditionSHEB1-STIM-GLYC      5.2335     2.0281   2.580   0.0104 *
## ---
## Signif. codes:  0 '***' 0.001 '**' 0.01 '*' 0.05 '.' 0.1 ' ' 1
##
## Residual standard error: 3.927 on 280 degrees of freedom
## Multiple R-squared:  0.9909, Adjusted R-squared:  0.9903
## F-statistic:  1607 on 19 and 280 DF,  p-value: < 2.2e-16
```

```
summary(emmeans(m2, pairwise ~ condition|treatment, infer=T))
```

```
## $emmeans
## treatment = a:
##  condition           emmean   SE  df lower.CL upper.CL t.ratio p.value
##  SHCTRL-NO-STIM-GLYC  17.96 1.01 280    15.96    19.96  17.712  <.0001
##  SHCTRL-STIM-GLYC     20.63 1.01 280    18.63    22.62  20.339  <.0001
##  SHEB1-NO-STIM-GLYC    6.81 1.01 280     4.81     8.80   6.713  <.0001
##  SHEB1-STIM-GLYC       7.20 1.01 280     5.21     9.20   7.105  <.0001
##
## treatment = b:
##  condition           emmean   SE  df lower.CL upper.CL t.ratio p.value
##  SHCTRL-NO-STIM-GLYC  17.65 1.01 280    15.66    19.65  17.410  <.0001
##  SHCTRL-STIM-GLYC     20.10 1.01 280    18.10    22.10  19.821  <.0001
##  SHEB1-NO-STIM-GLYC    7.03 1.01 280     5.03     9.02   6.930  <.0001
##  SHEB1-STIM-GLYC       7.12 1.01 280     5.13     9.12   7.025  <.0001
##
## treatment = c:
##  condition           emmean   SE  df lower.CL upper.CL t.ratio p.value
##  SHCTRL-NO-STIM-GLYC  94.29 1.01 280    92.30    96.29  92.985  <.0001
##  SHCTRL-STIM-GLYC     83.62 1.01 280    81.62    85.62  82.461  <.0001
##  SHEB1-NO-STIM-GLYC   61.06 1.01 280    59.07    63.06  60.217  <.0001
##  SHEB1-STIM-GLYC      45.80 1.01 280    43.80    47.79  45.163  <.0001
##
## treatment = d:
##  condition           emmean   SE  df lower.CL upper.CL t.ratio p.value
##  SHCTRL-NO-STIM-GLYC 120.64 1.01 280   118.64   122.63 118.965  <.0001
##  SHCTRL-STIM-GLYC    126.85 1.01 280   124.85   128.84 125.090  <.0001
##  SHEB1-NO-STIM-GLYC   73.85 1.01 280    71.86    75.85  72.830  <.0001
##  SHEB1-STIM-GLYC      68.47 1.01 280    66.47    70.47  67.520  <.0001
##
## treatment = e:
##  condition           emmean   SE  df lower.CL upper.CL t.ratio p.value
##  SHCTRL-NO-STIM-GLYC  10.49 1.01 280     8.49    12.48  10.343  <.0001
##  SHCTRL-STIM-GLYC     15.74 1.01 280    13.75    17.74  15.526  <.0001
##  SHEB1-NO-STIM-GLYC    4.01 1.01 280     2.01     6.00   3.950  0.0001
##  SHEB1-STIM-GLYC       4.97 1.01 280     2.97     6.96   4.897  <.0001
##
## Confidence level used: 0.95
```

```
##
## $contrasts
## treatment = a:
##  contrast                                   estimate   SE  df lower.CL
##  (SHCTRL-NO-STIM-GLYC) - (SHCTRL-STIM-GLYC)    -2.6646 1.43 280    -6.37
##  (SHCTRL-NO-STIM-GLYC) - (SHEB1-NO-STIM-GLYC)  11.1531 1.43 280     7.45
##  (SHCTRL-NO-STIM-GLYC) - (SHEB1-STIM-GLYC)     10.7562 1.43 280     7.05
##  (SHCTRL-STIM-GLYC) - (SHEB1-NO-STIM-GLYC)     13.8177 1.43 280    10.11
##  (SHCTRL-STIM-GLYC) - (SHEB1-STIM-GLYC)        13.4208 1.43 280     9.71
##  (SHEB1-NO-STIM-GLYC) - (SHEB1-STIM-GLYC)      -0.3968 1.43 280    -4.10
##  upper.CL t.ratio p.value
##      1.04  -1.858  0.2487
##     14.86   7.777  <.0001
##     14.46   7.500  <.0001
##     17.52   9.635  <.0001
##     17.13   9.358  <.0001
##      3.31  -0.277  0.9926
##
## treatment = b:
##  contrast                                   estimate   SE  df lower.CL
##  (SHCTRL-NO-STIM-GLYC) - (SHCTRL-STIM-GLYC)    -2.4455 1.43 280    -6.15
##  (SHCTRL-NO-STIM-GLYC) - (SHEB1-NO-STIM-GLYC)  10.6268 1.43 280     6.92
##  (SHCTRL-NO-STIM-GLYC) - (SHEB1-STIM-GLYC)     10.5302 1.43 280     6.82
##  (SHCTRL-STIM-GLYC) - (SHEB1-NO-STIM-GLYC)     13.0723 1.43 280     9.37
##  (SHCTRL-STIM-GLYC) - (SHEB1-STIM-GLYC)        12.9757 1.43 280     9.27
##  (SHEB1-NO-STIM-GLYC) - (SHEB1-STIM-GLYC)      -0.0966 1.43 280    -3.80
##  upper.CL t.ratio p.value
##      1.26  -1.705  0.3229
##     14.33   7.410  <.0001
##     14.24   7.343  <.0001
##     16.78   9.115  <.0001
##     16.68   9.048  <.0001
##      3.61  -0.067  0.9999
##
## treatment = c:
##  contrast                                   estimate   SE  df lower.CL
##  (SHCTRL-NO-STIM-GLYC) - (SHCTRL-STIM-GLYC)    10.6716 1.43 280     6.97
##  (SHCTRL-NO-STIM-GLYC) - (SHEB1-NO-STIM-GLYC)  33.2289 1.43 280    29.52
##  (SHCTRL-NO-STIM-GLYC) - (SHEB1-STIM-GLYC)     48.4947 1.43 280    44.79
##  (SHCTRL-STIM-GLYC) - (SHEB1-NO-STIM-GLYC)     22.5573 1.43 280    18.85
##  (SHCTRL-STIM-GLYC) - (SHEB1-STIM-GLYC)        37.8231 1.43 280    34.12
##  (SHEB1-NO-STIM-GLYC) - (SHEB1-STIM-GLYC)      15.2658 1.43 280    11.56
##  upper.CL t.ratio p.value
##     14.38   7.441  <.0001
##     36.94  23.171  <.0001
##     52.20  33.815  <.0001
##     26.26  15.729  <.0001
##     41.53  26.374  <.0001
##     18.97  10.645  <.0001
##
## treatment = d:
##  contrast                                   estimate   SE  df lower.CL
##  (SHCTRL-NO-STIM-GLYC) - (SHCTRL-STIM-GLYC)    -6.2104 1.43 280    -9.92
##  (SHCTRL-NO-STIM-GLYC) - (SHEB1-NO-STIM-GLYC)  46.7846 1.43 280    43.08
```

```
##  (SHCTRL-NO-STIM-GLYC) - (SHEB1-STIM-GLYC)     52.1686 1.43 280    48.46
##  (SHCTRL-STIM-GLYC) - (SHEB1-NO-STIM-GLYC)     52.9950 1.43 280    49.29
##  (SHCTRL-STIM-GLYC) - (SHEB1-STIM-GLYC)        58.3789 1.43 280    54.67
##  (SHEB1-NO-STIM-GLYC) - (SHEB1-STIM-GLYC)       5.3840 1.43 280     1.68
##  upper.CL t.ratio p.value
##     -2.50  -4.330  0.0001
##     50.49  32.623  <.0001
##     55.88  36.377  <.0001
##     56.70  36.953  <.0001
##     62.09  40.708  <.0001
##      9.09   3.754  0.0012
##
## treatment = e:
##  contrast                                     estimate   SE  df lower.CL
##  (SHCTRL-NO-STIM-GLYC) - (SHCTRL-STIM-GLYC)    -5.2554 1.43 280    -8.96
##  (SHCTRL-NO-STIM-GLYC) - (SHEB1-NO-STIM-GLYC)   6.4838 1.43 280     2.78
##  (SHCTRL-NO-STIM-GLYC) - (SHEB1-STIM-GLYC)      5.5227 1.43 280     1.82
##  (SHCTRL-STIM-GLYC) - (SHEB1-NO-STIM-GLYC)     11.7391 1.43 280     8.03
##  (SHCTRL-STIM-GLYC) - (SHEB1-STIM-GLYC)        10.7781 1.43 280     7.07
##  (SHEB1-NO-STIM-GLYC) - (SHEB1-STIM-GLYC)      -0.9610 1.43 280    -4.67
##  upper.CL t.ratio p.value
##     -1.55  -3.665  0.0017
##     10.19   4.521  0.0001
##      9.23   3.851  0.0008
##     15.45   8.186  <.0001
##     14.48   7.516  <.0001
##      2.75  -0.670  0.9083
##
## Confidence level used: 0.95
## Conf-level adjustment: tukey method for comparing a family of 4 estimates
## P value adjustment: tukey method for comparing a family of 4 estimates
```

```r
emmip(m2, condition ~ treatment) +
  theme_bw()+
  xlab('Treatment') +
  ylab('Value')
```

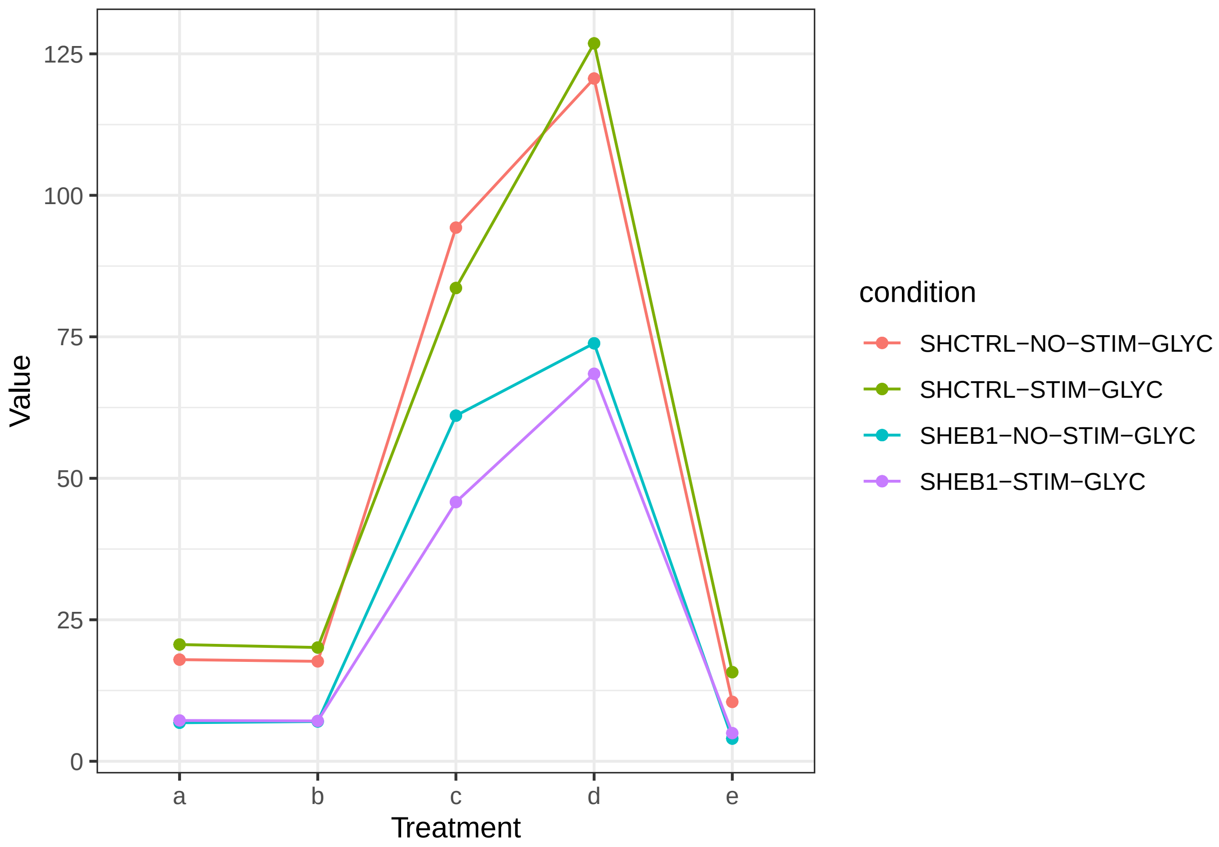


```
summary(pairs(emmeans(m2, "condition",by = "treatment",level = 0.95, infer = TRUE)))
```

```
## treatment = a:
##  contrast                                     estimate   SE  df t.ratio p.value
##  (SHCTRL-NO-STIM-GLYC) - (SHCTRL-STIM-GLYC)    -2.6646 1.43 280  -1.858  0.2487
##  (SHCTRL-NO-STIM-GLYC) - (SHEB1-NO-STIM-GLYC)  11.1531 1.43 280   7.777  <.0001
##  (SHCTRL-NO-STIM-GLYC) - (SHEB1-STIM-GLYC)     10.7562 1.43 280   7.500  <.0001
##  (SHCTRL-STIM-GLYC) - (SHEB1-NO-STIM-GLYC)     13.8177 1.43 280   9.635  <.0001
##  (SHCTRL-STIM-GLYC) - (SHEB1-STIM-GLYC)        13.4208 1.43 280   9.358  <.0001
##  (SHEB1-NO-STIM-GLYC) - (SHEB1-STIM-GLYC)      -0.3968 1.43 280  -0.277  0.9926
##
## treatment = b:
##  contrast                                     estimate   SE  df t.ratio p.value
##  (SHCTRL-NO-STIM-GLYC) - (SHCTRL-STIM-GLYC)    -2.4455 1.43 280  -1.705  0.3229
##  (SHCTRL-NO-STIM-GLYC) - (SHEB1-NO-STIM-GLYC)  10.6268 1.43 280   7.410  <.0001
##  (SHCTRL-NO-STIM-GLYC) - (SHEB1-STIM-GLYC)     10.5302 1.43 280   7.343  <.0001
##  (SHCTRL-STIM-GLYC) - (SHEB1-NO-STIM-GLYC)     13.0723 1.43 280   9.115  <.0001
##  (SHCTRL-STIM-GLYC) - (SHEB1-STIM-GLYC)        12.9757 1.43 280   9.048  <.0001
##  (SHEB1-NO-STIM-GLYC) - (SHEB1-STIM-GLYC)      -0.0966 1.43 280  -0.067  0.9999
##
## treatment = c:
##  contrast                                     estimate   SE  df t.ratio p.value
##  (SHCTRL-NO-STIM-GLYC) - (SHCTRL-STIM-GLYC)    10.6716 1.43 280   7.441  <.0001
##  (SHCTRL-NO-STIM-GLYC) - (SHEB1-NO-STIM-GLYC)  33.2289 1.43 280  23.171  <.0001
##  (SHCTRL-NO-STIM-GLYC) - (SHEB1-STIM-GLYC)     48.4947 1.43 280  33.815  <.0001
```

```
##  (SHCTRL-STIM-GLYC) - (SHEB1-NO-STIM-GLYC)     22.5573 1.43 280  15.729  <.0001
##  (SHCTRL-STIM-GLYC) - (SHEB1-STIM-GLYC)        37.8231 1.43 280  26.374  <.0001
##  (SHEB1-NO-STIM-GLYC) - (SHEB1-STIM-GLYC)      15.2658 1.43 280  10.645  <.0001
##
## treatment = d:
##  contrast                                     estimate   SE  df t.ratio p.value
##  (SHCTRL-NO-STIM-GLYC) - (SHCTRL-STIM-GLYC)    -6.2104 1.43 280  -4.330  0.0001
##  (SHCTRL-NO-STIM-GLYC) - (SHEB1-NO-STIM-GLYC)  46.7846 1.43 280  32.623  <.0001
##  (SHCTRL-NO-STIM-GLYC) - (SHEB1-STIM-GLYC)     52.1686 1.43 280  36.377  <.0001
##  (SHCTRL-STIM-GLYC) - (SHEB1-NO-STIM-GLYC)     52.9950 1.43 280  36.953  <.0001
##  (SHCTRL-STIM-GLYC) - (SHEB1-STIM-GLYC)        58.3789 1.43 280  40.708  <.0001
##  (SHEB1-NO-STIM-GLYC) - (SHEB1-STIM-GLYC)       5.3840 1.43 280   3.754  0.0012
##
## treatment = e:
##  contrast                                     estimate   SE  df t.ratio p.value
##  (SHCTRL-NO-STIM-GLYC) - (SHCTRL-STIM-GLYC)    -5.2554 1.43 280  -3.665  0.0017
##  (SHCTRL-NO-STIM-GLYC) - (SHEB1-NO-STIM-GLYC)   6.4838 1.43 280   4.521  0.0001
##  (SHCTRL-NO-STIM-GLYC) - (SHEB1-STIM-GLYC)      5.5227 1.43 280   3.851  0.0008
##  (SHCTRL-STIM-GLYC) - (SHEB1-NO-STIM-GLYC)     11.7391 1.43 280   8.186  <.0001
##  (SHCTRL-STIM-GLYC) - (SHEB1-STIM-GLYC)        10.7781 1.43 280   7.516  <.0001
##  (SHEB1-NO-STIM-GLYC) - (SHEB1-STIM-GLYC)      -0.9610 1.43 280  -0.670  0.9083
##
## P value adjustment: tukey method for comparing a family of 4 estimates
```

MitoStress OCR - Jurkat EB1 clones

```
ggplot(seahorse3, aes(time, value, colour=condition))+
  geom_point()+
  theme_bw()+
  xlab("Time (min)") +
  ylab('Value')
```

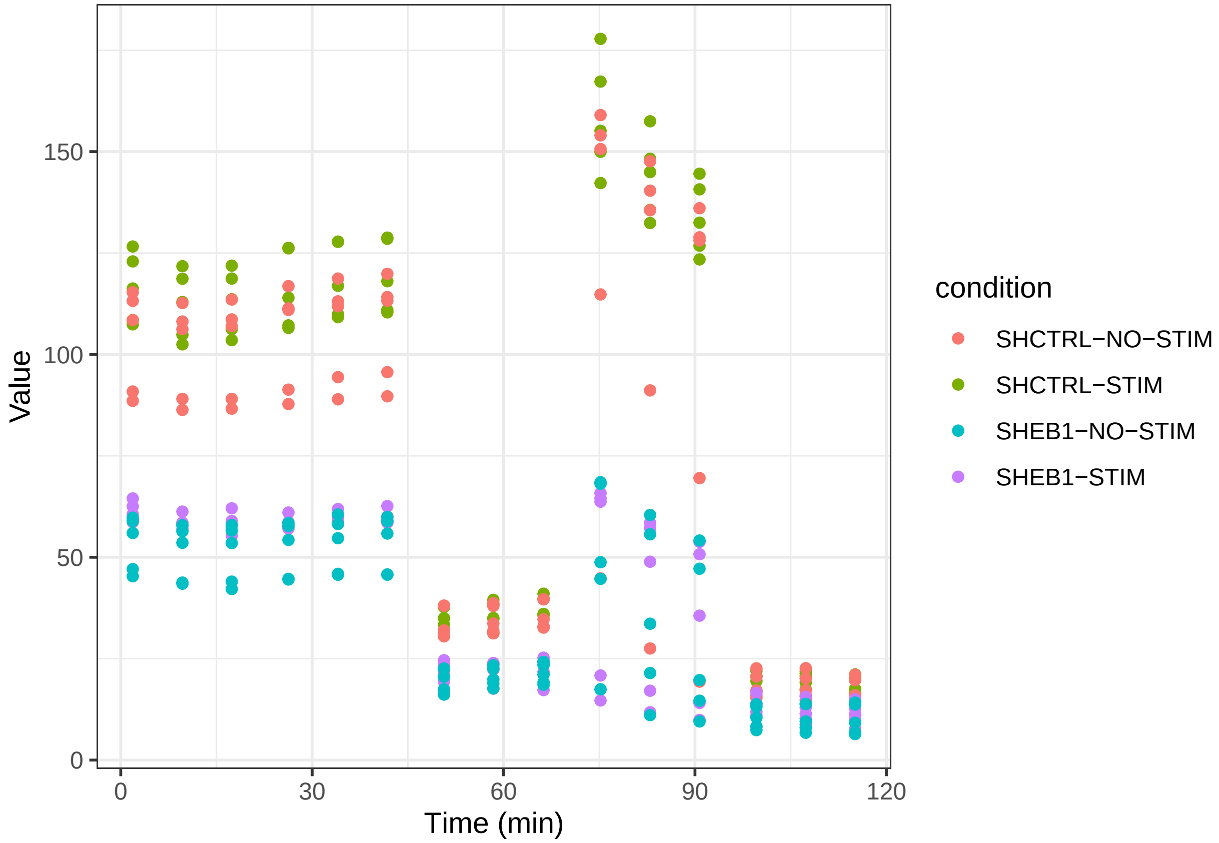


```
m3<-lm(value ~ treatment*condition, data=seahorse3)
summary(m3)
```

```
##
## Call:
## lm(formula = value ~ treatment * condition, data = seahorse3)
##
## Residuals:
##     Min      1Q  Median      3Q     Max
## -90.488  -3.547   0.045   4.774  49.203
##
## Coefficients:
##                              Estimate Std. Error t value Pr(>|t|)
## (Intercept)                  101.5747     3.6451  27.866  < 2e-16 ***
## treatmentb                     3.6173     5.1549   0.702 0.483441
## treatmentc                   -66.7329     5.1549 -12.946  < 2e-16 ***
## treatmentd                     8.2474     5.1549   1.600 0.110743
## treatmente                   -83.7212     5.1549 -16.241  < 2e-16 ***
## conditionSHCTRL-STIM          12.1857     5.1549   2.364 0.018766 *
## conditionSHEB1-NO-STIM       -49.8175     5.1549  -9.664  < 2e-16 ***
## conditionSHEB1-STIM          -42.2007     5.1549  -8.187 9.58e-15 ***
## treatmentb:conditionSHCTRL-STIM   0.5207     7.2901   0.071 0.943114
## treatmentc:conditionSHCTRL-STIM -11.1125     7.2901  -1.524 0.128558
## treatmentd:conditionSHCTRL-STIM  23.2685     7.2901   3.192 0.001575 **
## treatmente:conditionSHCTRL-STIM -10.3432     7.2901  -1.419 0.157071
```

```
## treatmentb:conditionSHEB1-NO-STIM  -2.6578     7.2901  -0.365 0.715708
## treatmentc:conditionSHEB1-NO-STIM  35.5139     7.2901   4.872 1.86e-06 ***
## treatmentd:conditionSHEB1-NO-STIM -21.6730     7.2901  -2.973 0.003206 **
## treatmente:conditionSHEB1-NO-STIM  42.3114     7.2901   5.804 1.75e-08 ***
## treatmentb:conditionSHEB1-STIM     -3.7531     7.2901  -0.515 0.607085
## treatmentc:conditionSHEB1-STIM     29.6049     7.2901   4.061 6.35e-05 ***
## treatmentd:conditionSHEB1-STIM    -28.4745     7.2901  -3.906 0.000118 ***
## treatmente:conditionSHEB1-STIM     36.1790     7.2901   4.963 1.21e-06 ***
## ---
## Signif. codes:  0 '***' 0.001 '**' 0.01 '*' 0.05 '.' 0.1 ' ' 1
##
## Residual standard error: 14.12 on 280 degrees of freedom
## Multiple R-squared:  0.899,  Adjusted R-squared:  0.8922
## F-statistic: 131.2 on 19 and 280 DF,  p-value: < 2.2e-16
```

```
summary(emmeans(m3, pairwise ~ condition|treatment, infer=T))
```

```
## $emmeans
## treatment = a:
##  condition      emmean   SE  df lower.CL upper.CL t.ratio p.value
##  SHCTRL-NO-STIM  101.6 3.65 280    94.40    108.7  27.866  <.0001
##  SHCTRL-STIM     113.8 3.65 280   106.59    120.9  31.209  <.0001
##  SHEB1-NO-STIM    51.8 3.65 280    44.58     58.9  14.199  <.0001
##  SHEB1-STIM       59.4 3.65 280    52.20     66.5  16.289  <.0001
##
## treatment = b:
##  condition      emmean   SE  df lower.CL upper.CL t.ratio p.value
##  SHCTRL-NO-STIM  105.2 3.65 280    98.02    112.4  28.859  <.0001
##  SHCTRL-STIM     117.9 3.65 280   110.72    125.1  32.345  <.0001
##  SHEB1-NO-STIM    52.7 3.65 280    45.54     59.9  14.463  <.0001
##  SHEB1-STIM       59.2 3.65 280    52.06     66.4  16.252  <.0001
##
## treatment = c:
##  condition      emmean   SE  df lower.CL upper.CL t.ratio p.value
##  SHCTRL-NO-STIM   34.8 3.65 280    27.67     42.0   9.559  <.0001
##  SHCTRL-STIM      35.9 3.65 280    28.74     43.1   9.853  <.0001
##  SHEB1-NO-STIM    20.5 3.65 280    13.36     27.7   5.635  <.0001
##  SHEB1-STIM       22.2 3.65 280    15.07     29.4   6.103  <.0001
##
## treatment = d:
##  condition      emmean   SE  df lower.CL upper.CL t.ratio p.value
##  SHCTRL-NO-STIM  109.8 3.65 280   102.65    117.0  30.129  <.0001
##  SHCTRL-STIM     145.3 3.65 280   138.10    152.5  39.856  <.0001
##  SHEB1-NO-STIM    38.3 3.65 280    31.16     45.5  10.516  <.0001
##  SHEB1-STIM       39.1 3.65 280    31.97     46.3  10.740  <.0001
##
## treatment = e:
##  condition      emmean   SE  df lower.CL upper.CL t.ratio p.value
##  SHCTRL-NO-STIM   17.9 3.65 280    10.68     25.0   4.898  <.0001
##  SHCTRL-STIM      19.7 3.65 280    12.52     26.9   5.403  <.0001
##  SHEB1-NO-STIM    10.3 3.65 280     3.17     17.5   2.839  0.0049
##  SHEB1-STIM       11.8 3.65 280     4.66     19.0   3.246  0.0013
##
## Confidence level used: 0.95
```

```
##
## $contrasts
## treatment = a:
##  contrast                           estimate   SE  df lower.CL upper.CL t.ratio
##  (SHCTRL-NO-STIM) - (SHCTRL-STIM)    -12.186 5.15 280  -25.509    1.137  -2.364
##  (SHCTRL-NO-STIM) - (SHEB1-NO-STIM)   49.817 5.15 280   36.495   63.140   9.664
##  (SHCTRL-NO-STIM) - (SHEB1-STIM)      42.201 5.15 280   28.878   55.524   8.187
##  (SHCTRL-STIM) - (SHEB1-NO-STIM)      62.003 5.15 280   48.680   75.326  12.028
##  (SHCTRL-STIM) - (SHEB1-STIM)         54.386 5.15 280   41.063   67.709  10.550
##  (SHEB1-NO-STIM) - (SHEB1-STIM)       -7.617 5.15 280  -20.940    5.706  -1.478
##  p.value
##   0.0866
##   <.0001
##   <.0001
##   <.0001
##   <.0001
##   0.4524
##
## treatment = b:
##  contrast                           estimate   SE  df lower.CL upper.CL t.ratio
##  (SHCTRL-NO-STIM) - (SHCTRL-STIM)    -12.706 5.15 280  -26.029    0.617  -2.465
##  (SHCTRL-NO-STIM) - (SHEB1-NO-STIM)   52.475 5.15 280   39.152   65.798  10.180
##  (SHCTRL-NO-STIM) - (SHEB1-STIM)      45.954 5.15 280   32.631   59.277   8.915
##  (SHCTRL-STIM) - (SHEB1-NO-STIM)      65.182 5.15 280   51.859   78.505  12.645
##  (SHCTRL-STIM) - (SHEB1-STIM)         58.660 5.15 280   45.337   71.983  11.379
##  (SHEB1-NO-STIM) - (SHEB1-STIM)       -6.521 5.15 280  -19.844    6.802  -1.265
##  p.value
##   0.0678
##   <.0001
##   <.0001
##   <.0001
##   <.0001
##   0.5859
##
## treatment = c:
##  contrast                           estimate   SE  df lower.CL upper.CL t.ratio
##  (SHCTRL-NO-STIM) - (SHCTRL-STIM)     -1.073 5.15 280  -14.396   12.250  -0.208
##  (SHCTRL-NO-STIM) - (SHEB1-NO-STIM)   14.304 5.15 280    0.981   27.627   2.775
##  (SHCTRL-NO-STIM) - (SHEB1-STIM)      12.596 5.15 280   -0.727   25.919   2.443
##  (SHCTRL-STIM) - (SHEB1-NO-STIM)      15.377 5.15 280    2.054   28.700   2.983
##  (SHCTRL-STIM) - (SHEB1-STIM)         13.669 5.15 280    0.346   26.992   2.652
##  (SHEB1-NO-STIM) - (SHEB1-STIM)       -1.708 5.15 280  -15.031   11.615  -0.331
##  p.value
##   0.9968
##   0.0299
##   0.0714
##   0.0163
##   0.0419
##   0.9874
##
## treatment = d:
##  contrast                           estimate   SE  df lower.CL upper.CL t.ratio
##  (SHCTRL-NO-STIM) - (SHCTRL-STIM)    -35.454 5.15 280  -48.777  -22.131  -6.878
##  (SHCTRL-NO-STIM) - (SHEB1-NO-STIM)   71.490 5.15 280   58.167   84.813  13.868
```

```
##  (SHCTRL-NO-STIM) - (SHEB1-STIM)      70.675 5.15 280   57.352   83.998  13.710
##  (SHCTRL-STIM) - (SHEB1-NO-STIM)     106.945 5.15 280   93.622  120.268  20.746
##  (SHCTRL-STIM) - (SHEB1-STIM)        106.129 5.15 280   92.806  119.452  20.588
##  (SHEB1-NO-STIM) - (SHEB1-STIM)       -0.815 5.15 280  -14.138   12.508  -0.158
##  p.value
##   <.0001
##   <.0001
##   <.0001
##   <.0001
##   <.0001
##   0.9986
##
## treatment = e:
##  contrast                           estimate   SE  df lower.CL upper.CL t.ratio
##  (SHCTRL-NO-STIM) - (SHCTRL-STIM)     -1.843 5.15 280  -15.165   11.480  -0.357
##  (SHCTRL-NO-STIM) - (SHEB1-NO-STIM)    7.506 5.15 280   -5.817   20.829   1.456
##  (SHCTRL-NO-STIM) - (SHEB1-STIM)       6.022 5.15 280   -7.301   19.345   1.168
##  (SHCTRL-STIM) - (SHEB1-NO-STIM)       9.349 5.15 280   -3.974   22.672   1.814
##  (SHCTRL-STIM) - (SHEB1-STIM)          7.864 5.15 280   -5.459   21.187   1.526
##  (SHEB1-NO-STIM) - (SHEB1-STIM)       -1.484 5.15 280  -14.807   11.839  -0.288
##  p.value
##   0.9843
##   0.4656
##   0.6475
##   0.2691
##   0.4236
##   0.9917
##
## Confidence level used: 0.95
## Conf-level adjustment: tukey method for comparing a family of 4 estimates
## P value adjustment: tukey method for comparing a family of 4 estimates
```

```r
emmip(m3, condition ~ treatment) +
  theme_bw()+
  xlab('Treatment') +
  ylab('Value')
```

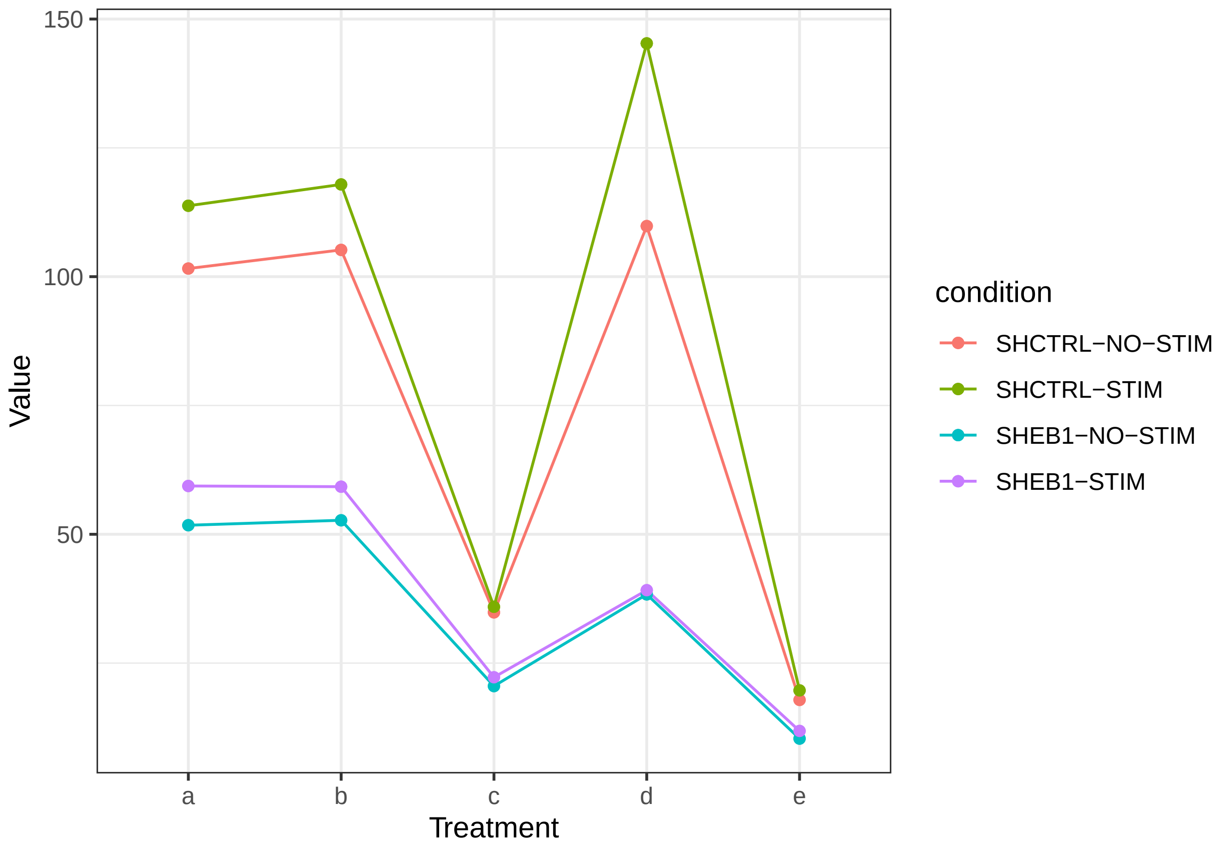


```
summary(pairs(emmeans(m3, "condition",by = "treatment",level = 0.95, infer = TRUE)))
```

```
## treatment = a:
##  contrast                             estimate   SE  df t.ratio p.value
##  (SHCTRL-NO-STIM) - (SHCTRL-STIM)      -12.186 5.15 280  -2.364  0.0866
##  (SHCTRL-NO-STIM) - (SHEB1-NO-STIM)     49.817 5.15 280   9.664  <.0001
##  (SHCTRL-NO-STIM) - (SHEB1-STIM)        42.201 5.15 280   8.187  <.0001
##  (SHCTRL-STIM) - (SHEB1-NO-STIM)        62.003 5.15 280  12.028  <.0001
##  (SHCTRL-STIM) - (SHEB1-STIM)           54.386 5.15 280  10.550  <.0001
##  (SHEB1-NO-STIM) - (SHEB1-STIM)         -7.617 5.15 280  -1.478  0.4524
##
## treatment = b:
##  contrast                             estimate   SE  df t.ratio p.value
##  (SHCTRL-NO-STIM) - (SHCTRL-STIM)      -12.706 5.15 280  -2.465  0.0678
##  (SHCTRL-NO-STIM) - (SHEB1-NO-STIM)     52.475 5.15 280  10.180  <.0001
##  (SHCTRL-NO-STIM) - (SHEB1-STIM)        45.954 5.15 280   8.915  <.0001
##  (SHCTRL-STIM) - (SHEB1-NO-STIM)        65.182 5.15 280  12.645  <.0001
##  (SHCTRL-STIM) - (SHEB1-STIM)           58.660 5.15 280  11.379  <.0001
##  (SHEB1-NO-STIM) - (SHEB1-STIM)         -6.521 5.15 280  -1.265  0.5859
##
## treatment = c:
##  contrast                             estimate   SE  df t.ratio p.value
##  (SHCTRL-NO-STIM) - (SHCTRL-STIM)       -1.073 5.15 280  -0.208  0.9968
##  (SHCTRL-NO-STIM) - (SHEB1-NO-STIM)     14.304 5.15 280   2.775  0.0299
##  (SHCTRL-NO-STIM) - (SHEB1-STIM)        12.596 5.15 280   2.443  0.0714
```

```
##  (SHCTRL-STIM) - (SHEB1-NO-STIM)      15.377 5.15 280   2.983  0.0163
##  (SHCTRL-STIM) - (SHEB1-STIM)         13.669 5.15 280   2.652  0.0419
##  (SHEB1-NO-STIM) - (SHEB1-STIM)       -1.708 5.15 280  -0.331  0.9874
##
## treatment = d:
##  contrast                            estimate   SE  df t.ratio p.value
##  (SHCTRL-NO-STIM) - (SHCTRL-STIM)    -35.454 5.15 280  -6.878  <.0001
##  (SHCTRL-NO-STIM) - (SHEB1-NO-STIM)   71.490 5.15 280  13.868  <.0001
##  (SHCTRL-NO-STIM) - (SHEB1-STIM)      70.675 5.15 280  13.710  <.0001
##  (SHCTRL-STIM) - (SHEB1-NO-STIM)     106.945 5.15 280  20.746  <.0001
##  (SHCTRL-STIM) - (SHEB1-STIM)        106.129 5.15 280  20.588  <.0001
##  (SHEB1-NO-STIM) - (SHEB1-STIM)       -0.815 5.15 280  -0.158  0.9986
##
## treatment = e:
##  contrast                            estimate   SE  df t.ratio p.value
##  (SHCTRL-NO-STIM) - (SHCTRL-STIM)     -1.843 5.15 280  -0.357  0.9843
##  (SHCTRL-NO-STIM) - (SHEB1-NO-STIM)    7.506 5.15 280   1.456  0.4656
##  (SHCTRL-NO-STIM) - (SHEB1-STIM)       6.022 5.15 280   1.168  0.6475
##  (SHCTRL-STIM) - (SHEB1-NO-STIM)       9.349 5.15 280   1.814  0.2691
##  (SHCTRL-STIM) - (SHEB1-STIM)          7.864 5.15 280   1.526  0.4236
##  (SHEB1-NO-STIM) - (SHEB1-STIM)       -1.484 5.15 280  -0.288  0.9917
##
## P value adjustment: tukey method for comparing a family of 4 estimates
```

MitoStress ECAR - Jurkat EB1 clones

```
ggplot(seahorse4, aes(time, value, colour=condition))+
  geom_point()+
  theme_bw()+
  xlab("Time (min)") +
  ylab('Value')
```

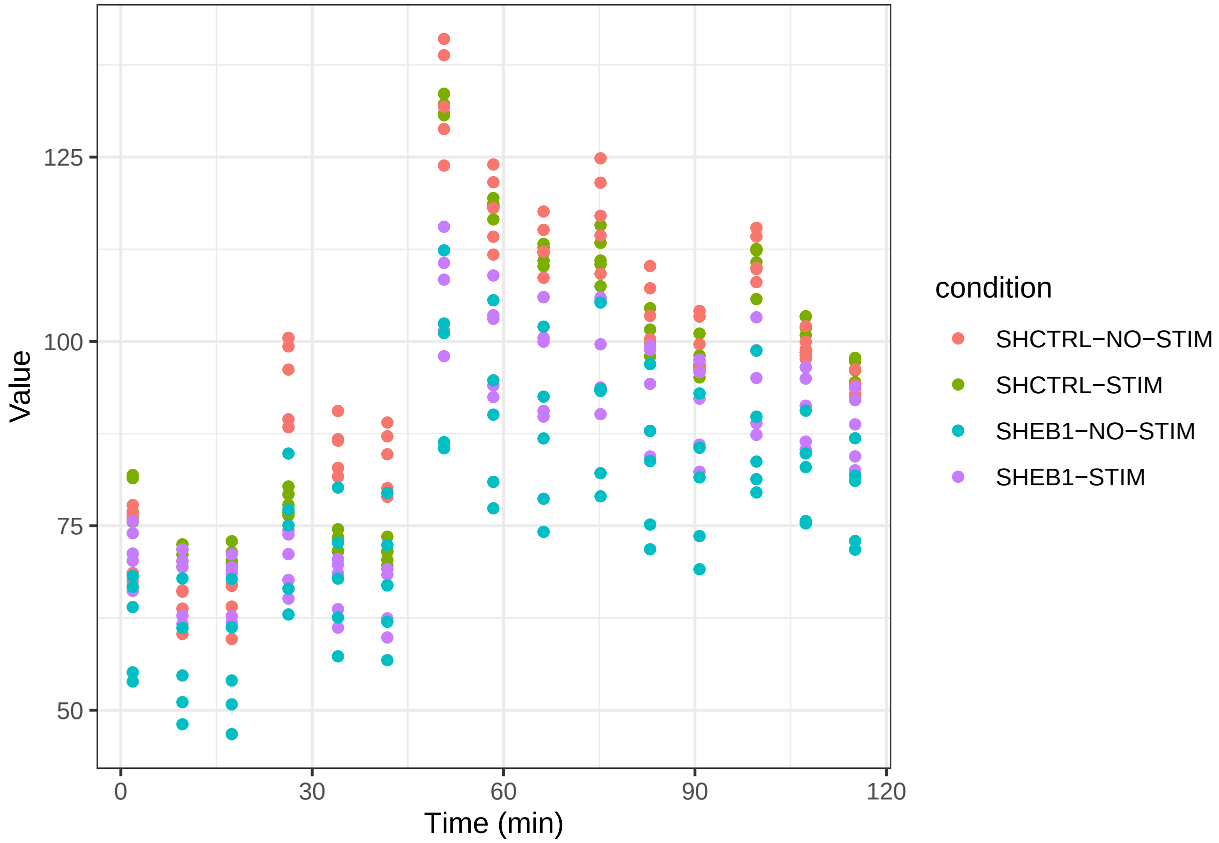


```
m4<-lm(value ~ treatment*condition, data=seahorse4)
summary(m4)
```

```
##
## Call:
## lm(formula = value ~ treatment * condition, data = seahorse4)
##
## Residuals:
##      Min       1Q   Median       3Q      Max
## -17.1919  -5.2366  -0.9988   4.5113  20.9710
##
## Coefficients:
##                                  Estimate Std. Error t value Pr(>|t|)
## (Intercept)                        68.024      1.927  35.296  < 2e-16 ***
## treatmentb                         20.115      2.725   7.380 1.81e-12 ***
## treatmentc                         52.875      2.725  19.400  < 2e-16 ***
## treatmentd                         39.171      2.725  14.372  < 2e-16 ***
## treatmente                         33.541      2.725  12.306  < 2e-16 ***
## conditionSHCTRL-STIM                5.335      2.725   1.957 0.051286 .
## conditionSHEB1-NO-STIM             -9.923      2.725  -3.641 0.000324 ***
## conditionSHEB1-STIM                 0.446      2.725   0.164 0.870139
## treatmentb:conditionSHCTRL-STIM   -19.362      3.854  -5.023 9.07e-07 ***
## treatmentc:conditionSHCTRL-STIM    -5.671      3.854  -1.471 0.142345
## treatmentd:conditionSHCTRL-STIM    -9.297      3.854  -2.412 0.016508 *
## treatmente:conditionSHCTRL-STIM    -3.900      3.854  -1.012 0.312460
```

```
## treatmentb:conditionSHEB1-NO-STIM   -8.567      3.854  -2.223 0.027044 *
## treatmentc:conditionSHEB1-NO-STIM  -19.596      3.854  -5.084 6.77e-07 ***
## treatmentd:conditionSHEB1-NO-STIM  -12.497      3.854  -3.242 0.001329 **
## treatmente:conditionSHEB1-NO-STIM   -9.180      3.854  -2.382 0.017908 *
## treatmentb:conditionSHEB1-STIM     -21.067      3.854  -5.466 1.02e-07 ***
## treatmentc:conditionSHEB1-STIM     -19.815      3.854  -5.141 5.14e-07 ***
## treatmentd:conditionSHEB1-STIM     -13.397      3.854  -3.476 0.000590 ***
## treatmente:conditionSHEB1-STIM     -10.716      3.854  -2.780 0.005801 **
## ---
## Signif. codes:  0 '***' 0.001 '**' 0.01 '*' 0.05 '.' 0.1 ' ' 1
##
## Residual standard error: 7.464 on 280 degrees of freedom
## Multiple R-squared:  0.8578, Adjusted R-squared:  0.8481
## F-statistic: 88.86 on 19 and 280 DF,  p-value: < 2.2e-16
```

```
summary(emmeans(m4, pairwise ~ condition|treatment, infer=T))
```

```
## $emmeans
## treatment = a:
##  condition      emmean   SE  df lower.CL upper.CL t.ratio p.value
##  SHCTRL-NO-STIM   68.0 1.93 280     64.2     71.8  35.296  <.0001
##  SHCTRL-STIM      73.4 1.93 280     69.6     77.2  38.065  <.0001
##  SHEB1-NO-STIM    58.1 1.93 280     54.3     61.9  30.147  <.0001
##  SHEB1-STIM       68.5 1.93 280     64.7     72.3  35.528  <.0001
##
## treatment = b:
##  condition      emmean   SE  df lower.CL upper.CL t.ratio p.value
##  SHCTRL-NO-STIM   88.1 1.93 280     84.3     91.9  45.733  <.0001
##  SHCTRL-STIM      74.1 1.93 280     70.3     77.9  38.455  <.0001
##  SHEB1-NO-STIM    69.6 1.93 280     65.9     73.4  36.139  <.0001
##  SHEB1-STIM       67.5 1.93 280     63.7     71.3  35.034  <.0001
##
## treatment = c:
##  condition      emmean   SE  df lower.CL upper.CL t.ratio p.value
##  SHCTRL-NO-STIM  120.9 1.93 280    117.1    124.7  62.732  <.0001
##  SHCTRL-STIM     120.6 1.93 280    116.8    124.4  62.558  <.0001
##  SHEB1-NO-STIM    91.4 1.93 280     87.6     95.2  47.415  <.0001
##  SHEB1-STIM      101.5 1.93 280     97.7    105.3  52.682  <.0001
##
## treatment = d:
##  condition      emmean   SE  df lower.CL upper.CL t.ratio p.value
##  SHCTRL-NO-STIM  107.2 1.93 280    103.4    111.0  55.621  <.0001
##  SHCTRL-STIM     103.2 1.93 280     99.4    107.0  53.565  <.0001
##  SHEB1-NO-STIM    84.8 1.93 280     81.0     88.6  43.988  <.0001
##  SHEB1-STIM       94.2 1.93 280     90.5     98.0  48.901  <.0001
##
## treatment = e:
##  condition      emmean   SE  df lower.CL upper.CL t.ratio p.value
##  SHCTRL-NO-STIM  101.6 1.93 280     97.8    105.4  52.700  <.0001
##  SHCTRL-STIM     103.0 1.93 280     99.2    106.8  53.445  <.0001
##  SHEB1-NO-STIM    82.5 1.93 280     78.7     86.3  42.788  <.0001
##  SHEB1-STIM       91.3 1.93 280     87.5     95.1  47.371  <.0001
##
## Confidence level used: 0.95
```

```
##
## $contrasts
## treatment = a:
##  contrast                          estimate   SE  df lower.CL upper.CL t.ratio
##  (SHCTRL-NO-STIM) - (SHCTRL-STIM)    -5.335 2.73 280   -12.38     1.71  -1.957
##  (SHCTRL-NO-STIM) - (SHEB1-NO-STIM)   9.923 2.73 280     2.88    16.97   3.641
##  (SHCTRL-NO-STIM) - (SHEB1-STIM)     -0.446 2.73 280    -7.49     6.60  -0.164
##  (SHCTRL-STIM) - (SHEB1-NO-STIM)     15.258 2.73 280     8.21    22.30   5.598
##  (SHCTRL-STIM) - (SHEB1-STIM)         4.889 2.73 280    -2.16    11.93   1.794
##  (SHEB1-NO-STIM) - (SHEB1-STIM)     -10.369 2.73 280   -17.41    -3.33  -3.805
##  p.value
##   0.2068
##   0.0018
##   0.9984
##   <.0001
##   0.2784
##   0.0010
##
## treatment = b:
##  contrast                          estimate   SE  df lower.CL upper.CL t.ratio
##  (SHCTRL-NO-STIM) - (SHCTRL-STIM)    14.027 2.73 280     6.98    21.07   5.146
##  (SHCTRL-NO-STIM) - (SHEB1-NO-STIM)  18.490 2.73 280    11.45    25.53   6.784
##  (SHCTRL-NO-STIM) - (SHEB1-STIM)     20.621 2.73 280    13.58    27.67   7.566
##  (SHCTRL-STIM) - (SHEB1-NO-STIM)      4.463 2.73 280    -2.58    11.51   1.638
##  (SHCTRL-STIM) - (SHEB1-STIM)         6.594 2.73 280    -0.45    13.64   2.419
##  (SHEB1-NO-STIM) - (SHEB1-STIM)       2.131 2.73 280    -4.91     9.17   0.782
##  p.value
##   <.0001
##   <.0001
##   <.0001
##   0.3592
##   0.0758
##   0.8627
##
## treatment = c:
##  contrast                          estimate   SE  df lower.CL upper.CL t.ratio
##  (SHCTRL-NO-STIM) - (SHCTRL-STIM)     0.336 2.73 280    -6.71     7.38   0.123
##  (SHCTRL-NO-STIM) - (SHEB1-NO-STIM)  29.520 2.73 280    22.48    36.56  10.831
##  (SHCTRL-NO-STIM) - (SHEB1-STIM)     19.369 2.73 280    12.33    26.41   7.107
##  (SHCTRL-STIM) - (SHEB1-NO-STIM)     29.184 2.73 280    22.14    36.23  10.708
##  (SHCTRL-STIM) - (SHEB1-STIM)        19.033 2.73 280    11.99    26.08   6.983
##  (SHEB1-NO-STIM) - (SHEB1-STIM)     -10.150 2.73 280   -17.19    -3.11  -3.724
##  p.value
##   0.9993
##   <.0001
##   <.0001
##   <.0001
##   <.0001
##   0.0013
##
## treatment = d:
##  contrast                          estimate   SE  df lower.CL upper.CL t.ratio
##  (SHCTRL-NO-STIM) - (SHCTRL-STIM)     3.962 2.73 280    -3.08    11.01   1.454
##  (SHCTRL-NO-STIM) - (SHEB1-NO-STIM)  22.420 2.73 280    15.38    29.46   8.226
```

```
##  (SHCTRL-NO-STIM) - (SHEB1-STIM)      12.951 2.73 280     5.91    20.00   4.752
##  (SHCTRL-STIM) - (SHEB1-NO-STIM)      18.459 2.73 280    11.41    25.50   6.772
##  (SHCTRL-STIM) - (SHEB1-STIM)          8.989 2.73 280     1.94    16.03   3.298
##  (SHEB1-NO-STIM) - (SHEB1-STIM)       -9.470 2.73 280   -16.51    -2.43  -3.474
##  p.value
##   0.4671
##   <.0001
##   <.0001
##   <.0001
##   0.0060
##   0.0033
##
## treatment = e:
##  contrast                           estimate   SE  df lower.CL upper.CL t.ratio
##  (SHCTRL-NO-STIM) - (SHCTRL-STIM)     -1.435 2.73 280    -8.48     5.61  -0.526
##  (SHCTRL-NO-STIM) - (SHEB1-NO-STIM)   19.103 2.73 280    12.06    26.15   7.009
##  (SHCTRL-NO-STIM) - (SHEB1-STIM)      10.270 2.73 280     3.23    17.31   3.768
##  (SHCTRL-STIM) - (SHEB1-NO-STIM)      20.538 2.73 280    13.49    27.58   7.535
##  (SHCTRL-STIM) - (SHEB1-STIM)         11.705 2.73 280     4.66    18.75   4.295
##  (SHEB1-NO-STIM) - (SHEB1-STIM)       -8.833 2.73 280   -15.88    -1.79  -3.241
##  p.value
##   0.9526
##   <.0001
##   0.0011
##   <.0001
##   0.0001
##   0.0073
##
## Confidence level used: 0.95
## Conf-level adjustment: tukey method for comparing a family of 4 estimates
## P value adjustment: tukey method for comparing a family of 4 estimates
```

```
emmip(m4, condition ~ treatment) +
  theme_bw()+
  xlab('Treatment') +
  ylab('Value')
```

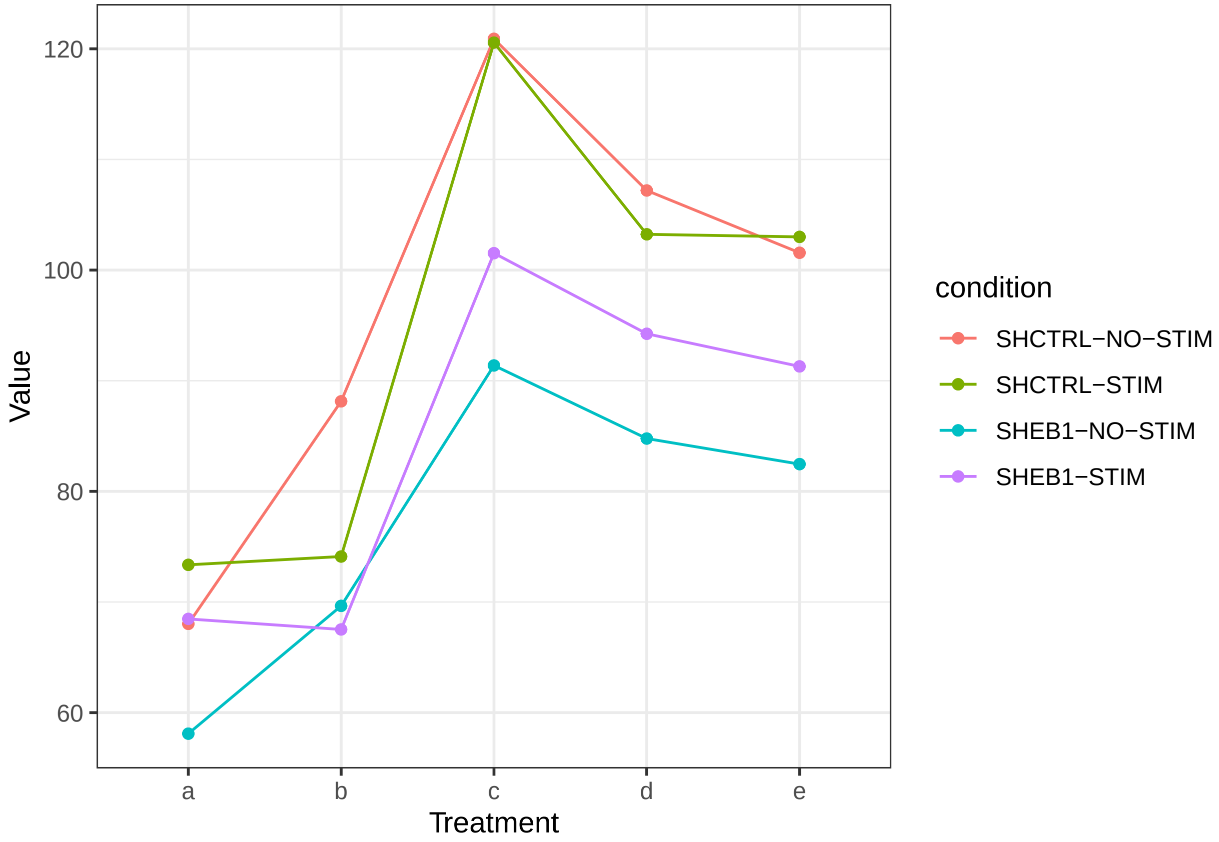


```
summary(pairs(emmeans(m4, "condition",by = "treatment",level = 0.95, infer = TRUE)))
```

```
## treatment = a:
##  contrast                          estimate   SE  df t.ratio p.value
##  (SHCTRL-NO-STIM) - (SHCTRL-STIM)    -5.335 2.73 280  -1.957  0.2068
##  (SHCTRL-NO-STIM) - (SHEB1-NO-STIM)   9.923 2.73 280   3.641  0.0018
##  (SHCTRL-NO-STIM) - (SHEB1-STIM)     -0.446 2.73 280  -0.164  0.9984
##  (SHCTRL-STIM) - (SHEB1-NO-STIM)     15.258 2.73 280   5.598  <.0001
##  (SHCTRL-STIM) - (SHEB1-STIM)         4.889 2.73 280   1.794  0.2784
##  (SHEB1-NO-STIM) - (SHEB1-STIM)     -10.369 2.73 280  -3.805  0.0010
##
## treatment = b:
##  contrast                          estimate   SE  df t.ratio p.value
##  (SHCTRL-NO-STIM) - (SHCTRL-STIM)    14.027 2.73 280   5.146  <.0001
##  (SHCTRL-NO-STIM) - (SHEB1-NO-STIM)  18.490 2.73 280   6.784  <.0001
##  (SHCTRL-NO-STIM) - (SHEB1-STIM)     20.621 2.73 280   7.566  <.0001
##  (SHCTRL-STIM) - (SHEB1-NO-STIM)      4.463 2.73 280   1.638  0.3592
##  (SHCTRL-STIM) - (SHEB1-STIM)         6.594 2.73 280   2.419  0.0758
##  (SHEB1-NO-STIM) - (SHEB1-STIM)       2.131 2.73 280   0.782  0.8627
##
## treatment = c:
##  contrast                          estimate   SE  df t.ratio p.value
##  (SHCTRL-NO-STIM) - (SHCTRL-STIM)     0.336 2.73 280   0.123  0.9993
##  (SHCTRL-NO-STIM) - (SHEB1-NO-STIM)  29.520 2.73 280  10.831  <.0001
##  (SHCTRL-NO-STIM) - (SHEB1-STIM)     19.369 2.73 280   7.107  <.0001
```

```
##  (SHCTRL-STIM) - (SHEB1-NO-STIM)      29.184 2.73 280  10.708  <.0001
##  (SHCTRL-STIM) - (SHEB1-STIM)         19.033 2.73 280   6.983  <.0001
##  (SHEB1-NO-STIM) - (SHEB1-STIM)      -10.150 2.73 280  -3.724  0.0013
##
## treatment = d:
##  contrast                           estimate   SE  df t.ratio p.value
##  (SHCTRL-NO-STIM) - (SHCTRL-STIM)      3.962 2.73 280   1.454  0.4671
##  (SHCTRL-NO-STIM) - (SHEB1-NO-STIM)   22.420 2.73 280   8.226  <.0001
##  (SHCTRL-NO-STIM) - (SHEB1-STIM)      12.951 2.73 280   4.752  <.0001
##  (SHCTRL-STIM) - (SHEB1-NO-STIM)      18.459 2.73 280   6.772  <.0001
##  (SHCTRL-STIM) - (SHEB1-STIM)          8.989 2.73 280   3.298  0.0060
##  (SHEB1-NO-STIM) - (SHEB1-STIM)       -9.470 2.73 280  -3.474  0.0033
##
## treatment = e:
##  contrast                           estimate   SE  df t.ratio p.value
##  (SHCTRL-NO-STIM) - (SHCTRL-STIM)     -1.435 2.73 280  -0.526  0.9526
##  (SHCTRL-NO-STIM) - (SHEB1-NO-STIM)   19.103 2.73 280   7.009  <.0001
##  (SHCTRL-NO-STIM) - (SHEB1-STIM)      10.270 2.73 280   3.768  0.0011
##  (SHCTRL-STIM) - (SHEB1-NO-STIM)      20.538 2.73 280   7.535  <.0001
##  (SHCTRL-STIM) - (SHEB1-STIM)         11.705 2.73 280   4.295  0.0001
##  (SHEB1-NO-STIM) - (SHEB1-STIM)       -8.833 2.73 280  -3.241  0.0073
##
## P value adjustment: tukey method for comparing a family of 4 estimates
```

Glycolysis Stress OCR - Lymphoblasts

```
ggplot(seahorse5, aes(time, value, colour=condition))+
  geom_point()+
  theme_bw()+
  xlab("Time (min)") +
  ylab('Value')+
  facet_wrap(~ind)
```

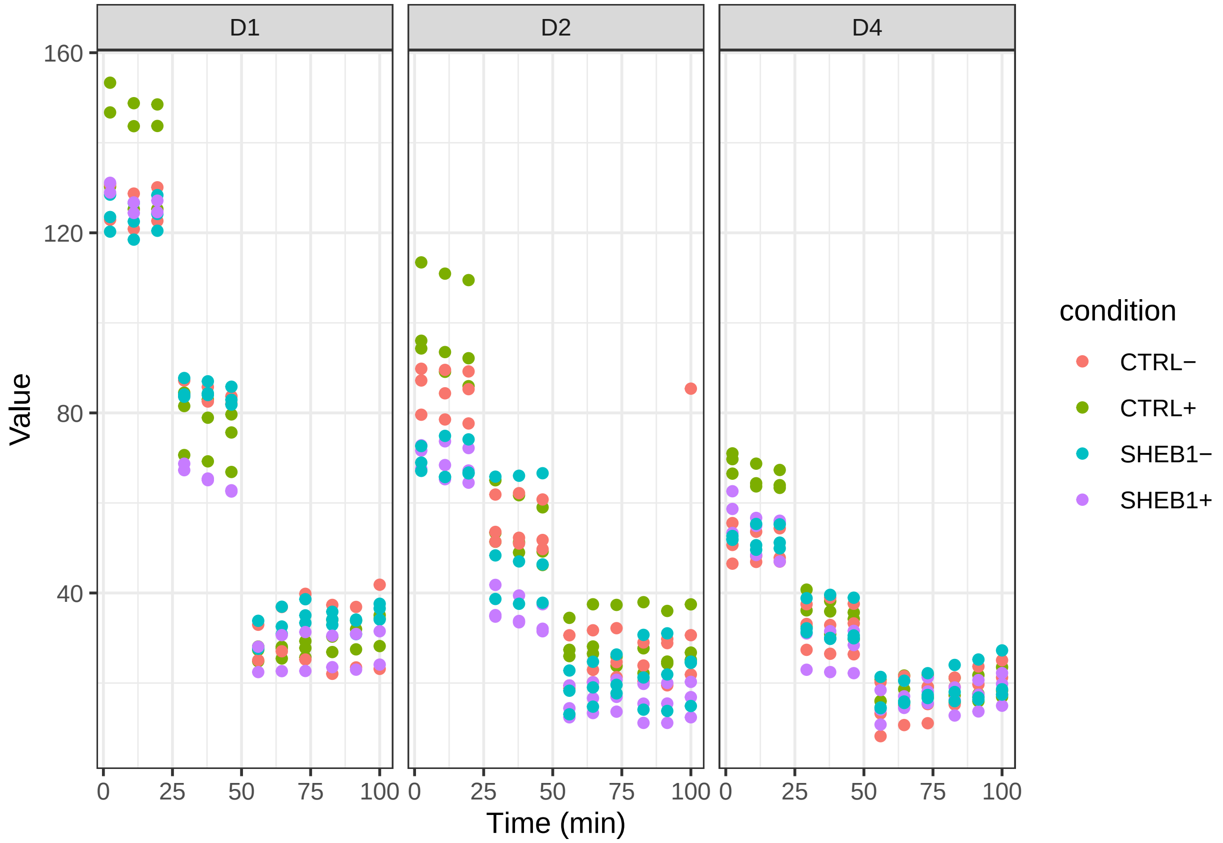


```
m5<-lmer(value ~ treatment*condition + (1|ind), data=seahorse5)
summary(m5)
```

```
## Linear mixed model fit by REML. t-tests use Satterthwaite's method [
## lmerModLmerTest]
## Formula: value ~ treatment * condition + (1 | ind)
##    Data: seahorse5
##
## REML criterion at convergence: 3151
##
## Scaled residuals:
##     Min      1Q  Median      3Q     Max
## -2.1796 -0.7549 -0.0583  0.6661  4.7421
##
## Random effects:
##  Groups   Name        Variance Std.Dev.
##  ind      (Intercept) 327.7    18.10
##  Residual             154.4    12.43
## Number of obs: 408, groups:  ind, 3
##
## Fixed effects:
##                              Estimate Std. Error        df t value Pr(>|t|)
## (Intercept)                  84.42625   10.75470   2.22658   7.850 0.011537 *
## treatmentb                  -28.10904    3.58690 389.99931  -7.837 4.45e-14 ***
## treatmentc                  -59.35218    3.58690 389.99931 -16.547  < 2e-16 ***
```

```
## treatmentd                 -54.75655    3.58690 389.99931 -15.266  < 2e-16 ***
## conditionCTRL+              17.39578    3.48767 390.00236   4.988 9.21e-07 ***
## conditionSHEB1-             -2.80067    3.48767 390.00236  -0.803 0.422451
## conditionSHEB1+             -3.68045    3.58690 389.99931  -1.026 0.305489
## treatmentb:conditionCTRL+  -18.51707    4.92973 389.99931  -3.756 0.000199 ***
## treatmentc:conditionCTRL+  -17.36122    4.92973 389.99931  -3.522 0.000479 ***
## treatmentd:conditionCTRL+  -20.66765    4.92973 389.99931  -4.192 3.42e-05 ***
## treatmentb:conditionSHEB1-   2.66090    4.92973 389.99931   0.540 0.589666
## treatmentc:conditionSHEB1-   1.12210    4.92973 389.99931   0.228 0.820062
## treatmentd:conditionSHEB1-  -1.44113    4.92973 389.99931  -0.292 0.770186
## treatmentb:conditionSHEB1+ -10.06586    5.07265 389.99931  -1.984 0.047918 *
## treatmentc:conditionSHEB1+   0.03046    5.07265 389.99931   0.006 0.995212
## treatmentd:conditionSHEB1+  -4.27251    5.07265 389.99931  -0.842 0.400157
## ---
## Signif. codes:  0 '***' 0.001 '**' 0.01 '*' 0.05 '.' 0.1 ' ' 1

##
## Correlation matrix not shown by default, as p = 16 > 12.
## Use print(x, correlation=TRUE)  or
##     vcov(x)        if you need it
```

```
summary(emmeans(m5, pairwise ~ condition|treatment, infer=T))
```

```
## Cannot use mode = "kenward-roger" because *pbkrtest* package is not installed
```

```
## $emmeans
## treatment = a:
##  condition emmean   SE   df lower.CL upper.CL t.ratio p.value
##  CTRL-       84.4 10.8 2.23   42.385    126.5   7.850  0.0115
##  CTRL+      101.8 10.7 2.20   59.467    144.2   9.498  0.0080
##  SHEB1-      81.6 10.7 2.20   39.270    124.0   7.614  0.0128
##  SHEB1+      80.7 10.8 2.23   38.704    122.8   7.508  0.0127
##
## treatment = b:
##  condition emmean   SE   df lower.CL upper.CL t.ratio p.value
##  CTRL-       56.3 10.8 2.23   14.276     98.4   5.237  0.0274
##  CTRL+       55.2 10.7 2.20   12.841     97.6   5.148  0.0292
##  SHEB1-      56.2 10.7 2.20   13.822     98.5   5.240  0.0281
##  SHEB1+      42.6 10.8 2.23    0.529     84.6   3.958  0.0488
##
## treatment = c:
##  condition emmean   SE   df lower.CL upper.CL t.ratio p.value
##  CTRL-       25.1 10.8 2.23  -16.968     67.1   2.331  0.1321
##  CTRL+       25.1 10.7 2.20  -17.247     67.5   2.342  0.1325
##  SHEB1-      23.4 10.7 2.20  -18.960     65.8   2.182  0.1492
##  SHEB1+      21.4 10.8 2.23  -20.618     63.5   1.992  0.1715
##
## treatment = d:
##  condition emmean   SE   df lower.CL upper.CL t.ratio p.value
##  CTRL-       29.7 10.8 2.23  -12.372     71.7   2.759  0.0979
##  CTRL+       26.4 10.7 2.20  -15.957     68.8   2.462  0.1215
##  SHEB1-      25.4 10.7 2.20  -16.927     67.8   2.372  0.1297
##  SHEB1+      21.7 10.8 2.23  -20.325     63.8   2.019  0.1678
```

```
##
## Degrees-of-freedom method: satterthwaite
## Confidence level used: 0.95
##
## $contrasts
## treatment = a:
##  contrast            estimate   SE  df lower.CL upper.CL t.ratio p.value
##  (CTRL-) - (CTRL+)   -17.3958 3.49 390   -26.39    -8.40  -4.988  <.0001
##  (CTRL-) - (SHEB1-)    2.8007 3.49 390    -6.20    11.80   0.803  0.8530
##  (CTRL-) - (SHEB1+)    3.6805 3.59 390    -5.57    12.94   1.026  0.7343
##  (CTRL+) - (SHEB1-)   20.1965 3.38 390    11.47    28.92   5.972  <.0001
##  (CTRL+) - (SHEB1+)   21.0762 3.49 390    12.08    30.07   6.043  <.0001
##  (SHEB1-) - (SHEB1+)   0.8798 3.49 390    -8.12     9.88   0.252  0.9944
##
## treatment = b:
##  contrast            estimate   SE  df lower.CL upper.CL t.ratio p.value
##  (CTRL-) - (CTRL+)     1.1213 3.49 390    -7.88    10.12   0.322  0.9885
##  (CTRL-) - (SHEB1-)    0.1398 3.49 390    -8.86     9.14   0.040  1.0000
##  (CTRL-) - (SHEB1+)   13.7463 3.59 390     4.49    23.00   3.832  0.0008
##  (CTRL+) - (SHEB1-)   -0.9815 3.38 390    -9.71     7.74  -0.290  0.9915
##  (CTRL+) - (SHEB1+)   12.6250 3.49 390     3.63    21.62   3.620  0.0019
##  (SHEB1-) - (SHEB1+)  13.6065 3.49 390     4.61    22.61   3.901  0.0006
##
## treatment = c:
##  contrast            estimate   SE  df lower.CL upper.CL t.ratio p.value
##  (CTRL-) - (CTRL+)    -0.0346 3.49 390    -9.03     8.96  -0.010  1.0000
##  (CTRL-) - (SHEB1-)    1.6786 3.49 390    -7.32    10.68   0.481  0.9632
##  (CTRL-) - (SHEB1+)    3.6500 3.59 390    -5.60    12.90   1.018  0.7392
##  (CTRL+) - (SHEB1-)    1.7131 3.38 390    -7.01    10.44   0.507  0.9575
##  (CTRL+) - (SHEB1+)    3.6846 3.49 390    -5.31    12.68   1.056  0.7162
##  (SHEB1-) - (SHEB1+)   1.9714 3.49 390    -7.03    10.97   0.565  0.9423
##
## treatment = d:
##  contrast            estimate   SE  df lower.CL upper.CL t.ratio p.value
##  (CTRL-) - (CTRL+)     3.2719 3.49 390    -5.73    12.27   0.938  0.7843
##  (CTRL-) - (SHEB1-)    4.2418 3.49 390    -4.76    13.24   1.216  0.6169
##  (CTRL-) - (SHEB1+)    7.9530 3.59 390    -1.30    17.21   2.217  0.1204
##  (CTRL+) - (SHEB1-)    0.9699 3.38 390    -7.76     9.70   0.287  0.9918
##  (CTRL+) - (SHEB1+)    4.6811 3.49 390    -4.32    13.68   1.342  0.5366
##  (SHEB1-) - (SHEB1+)   3.7112 3.49 390    -5.29    12.71   1.064  0.7116
##
## Degrees-of-freedom method: satterthwaite
## Confidence level used: 0.95
## Conf-level adjustment: tukey method for comparing a family of 4 estimates
## P value adjustment: tukey method for comparing a family of 4 estimates
```

```r
emmip(m5, condition ~ treatment) +
  theme_bw()+
  xlab('Treatment') +
  ylab('Value')
```

```
## Cannot use mode = "kenward-roger" because *pbkrtest* package is not installed
```

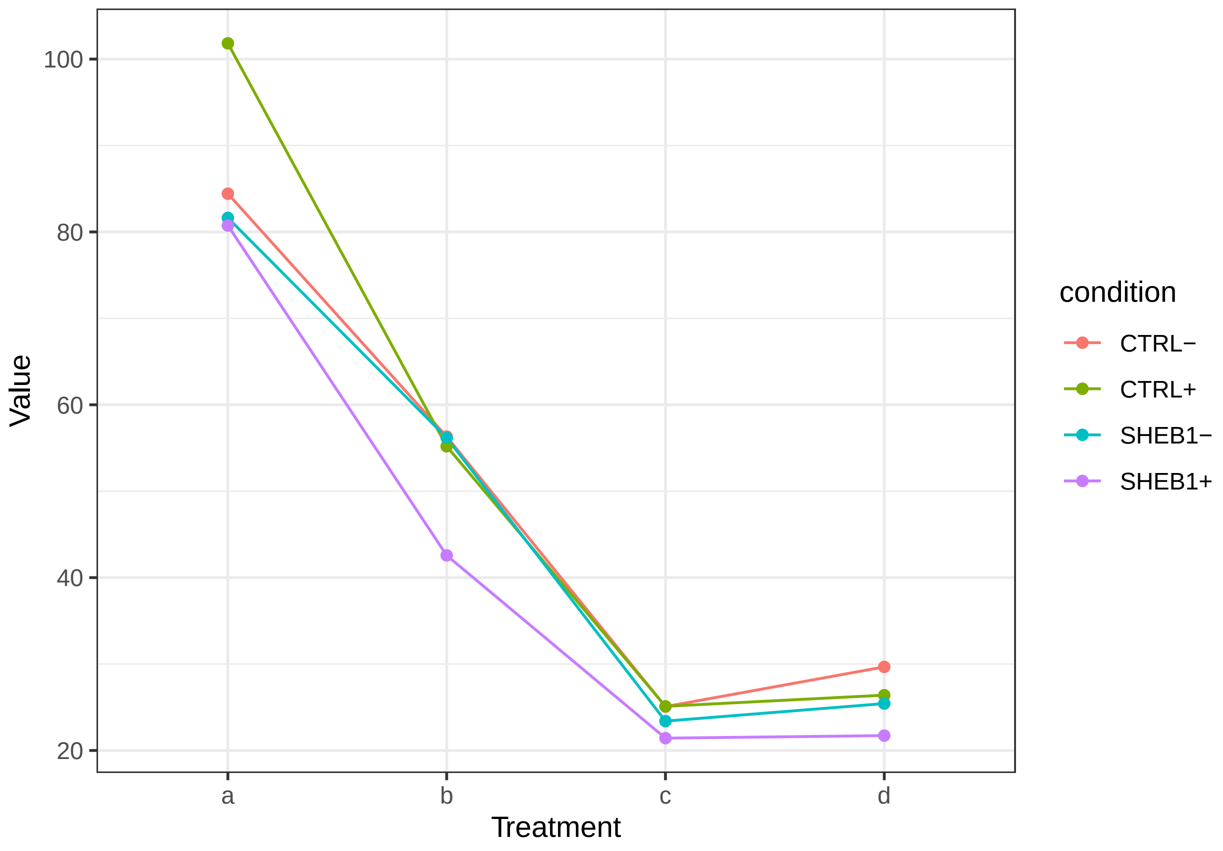


```
summary(pairs(emmeans(m5, "condition",by = "treatment",level = 0.95, infer = TRUE)))
```

```
## Cannot use mode = "kenward-roger" because *pbkrtest* package is not installed
```

```
## treatment = a:
##  contrast            estimate   SE  df t.ratio p.value
##  (CTRL-) - (CTRL+)   -17.3958 3.49 390  -4.988  <.0001
##  (CTRL-) - (SHEB1-)    2.8007 3.49 390   0.803  0.8530
##  (CTRL-) - (SHEB1+)    3.6805 3.59 390   1.026  0.7343
##  (CTRL+) - (SHEB1-)   20.1965 3.38 390   5.972  <.0001
##  (CTRL+) - (SHEB1+)   21.0762 3.49 390   6.043  <.0001
##  (SHEB1-) - (SHEB1+)   0.8798 3.49 390   0.252  0.9944
##
## treatment = b:
##  contrast            estimate   SE  df t.ratio p.value
##  (CTRL-) - (CTRL+)     1.1213 3.49 390   0.322  0.9885
##  (CTRL-) - (SHEB1-)    0.1398 3.49 390   0.040  1.0000
##  (CTRL-) - (SHEB1+)   13.7463 3.59 390   3.832  0.0008
##  (CTRL+) - (SHEB1-)   -0.9815 3.38 390  -0.290  0.9915
##  (CTRL+) - (SHEB1+)   12.6250 3.49 390   3.620  0.0019
##  (SHEB1-) - (SHEB1+)  13.6065 3.49 390   3.901  0.0006
##
## treatment = c:
##  contrast            estimate   SE  df t.ratio p.value
##  (CTRL-) - (CTRL+)    -0.0346 3.49 390  -0.010  1.0000
```

```
##  (CTRL-) - (SHEB1-)    1.6786 3.49 390   0.481  0.9632
##  (CTRL-) - (SHEB1+)    3.6500 3.59 390   1.018  0.7392
##  (CTRL+) - (SHEB1-)    1.7131 3.38 390   0.507  0.9575
##  (CTRL+) - (SHEB1+)    3.6846 3.49 390   1.056  0.7162
##  (SHEB1-) - (SHEB1+)   1.9714 3.49 390   0.565  0.9423
##
## treatment = d:
##  contrast            estimate   SE  df t.ratio p.value
##  (CTRL-) - (CTRL+)     3.2719 3.49 390   0.938  0.7843
##  (CTRL-) - (SHEB1-)    4.2418 3.49 390   1.216  0.6169
##  (CTRL-) - (SHEB1+)    7.9530 3.59 390   2.217  0.1204
##  (CTRL+) - (SHEB1-)    0.9699 3.38 390   0.287  0.9918
##  (CTRL+) - (SHEB1+)    4.6811 3.49 390   1.342  0.5366
##  (SHEB1-) - (SHEB1+)   3.7112 3.49 390   1.064  0.7116
##
## Degrees-of-freedom method: satterthwaite
## P value adjustment: tukey method for comparing a family of 4 estimates
```

Glycolysis Stress ECAR - Lymphoblasts

```
ggplot(seahorse6, aes(time, value, colour=condition))+
  geom_point()+
  theme_bw()+
  xlab("Time (min)") +
  ylab('Value')+
  facet_wrap(~ind)
```

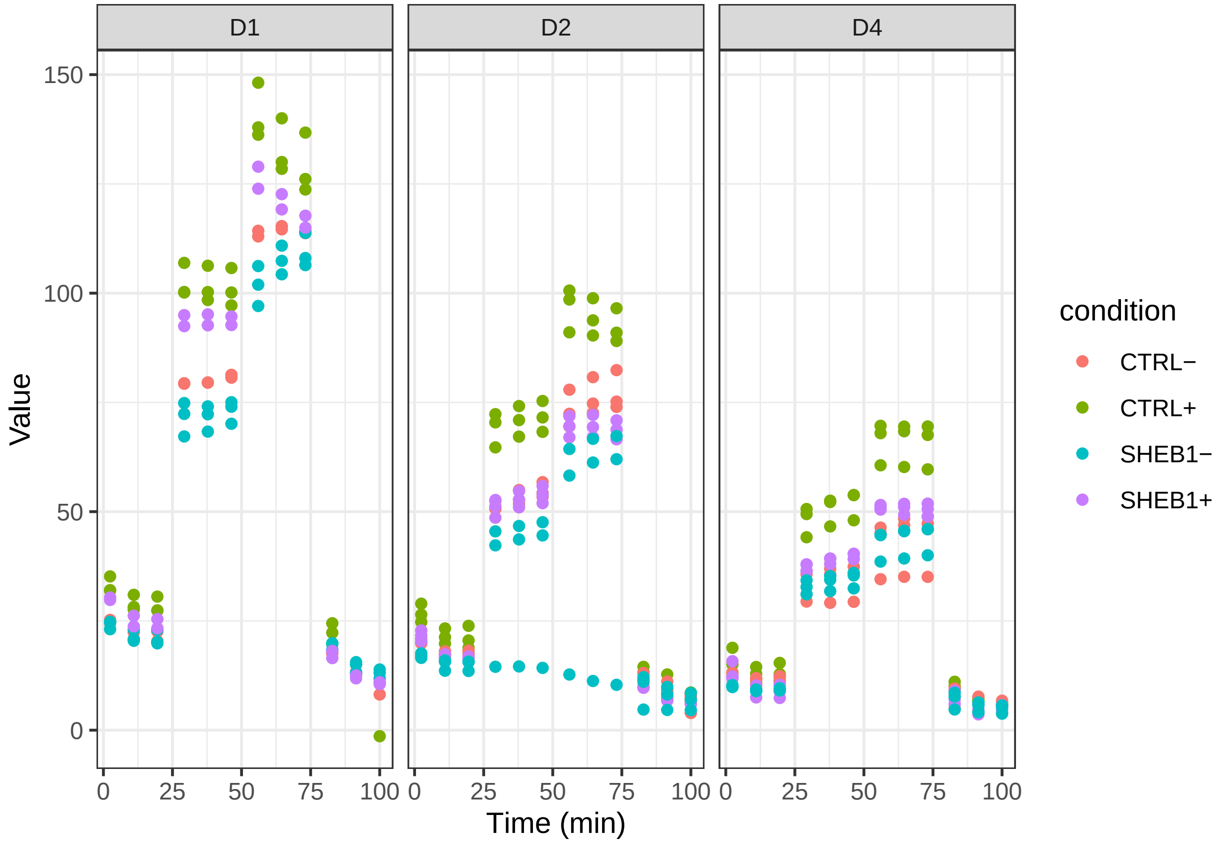


```
m6<-lmer(value ~ treatment*condition + (1|ind), data=seahorse6)
summary(m6)
```

```
## Linear mixed model fit by REML. t-tests use Satterthwaite's method [
## lmerModLmerTest]
## Formula: value ~ treatment * condition + (1 | ind)
##    Data: seahorse6
##
## REML criterion at convergence: 3127.5
##
## Scaled residuals:
##     Min      1Q  Median      3Q     Max
## -4.2059 -0.6507  0.1572  0.6069  2.6581
##
## Random effects:
##  Groups   Name        Variance Std.Dev.
##  ind      (Intercept) 307.6    17.54
##  Residual             145.4    12.06
## Number of obs: 408, groups:  ind, 3
##
## Fixed effects:
##                              Estimate Std. Error       df t value Pr(>|t|)
## (Intercept)                   19.3651    10.4207   2.2273   1.858 0.191121
## treatmentb                    35.6755     3.4810 389.9992  10.249  < 2e-16 ***
## treatmentc                    56.0404     3.4810 389.9992  16.099  < 2e-16 ***
```

```
## treatmentd                 -7.8007     3.4810 389.9992  -2.241 0.025593 *
## conditionCTRL+              2.9938     3.3847 390.0023   0.885 0.376966
## conditionSHEB1-            -3.5795     3.3847 390.0023  -1.058 0.290910
## conditionSHEB1+             0.4347     3.4810 389.9992   0.125 0.900695
## treatmentb:conditionCTRL+   16.0927     4.7842 389.9992   3.364 0.000845 ***
## treatmentc:conditionCTRL+   19.7494     4.7842 389.9992   4.128 4.48e-05 ***
## treatmentd:conditionCTRL+   -3.8167     4.7842 389.9992  -0.798 0.425486
## treatmentb:conditionSHEB1-  -4.5962     4.7842 389.9992  -0.961 0.337292
## treatmentc:conditionSHEB1-  -6.6210     4.7842 389.9992  -1.384 0.167171
## treatmentd:conditionSHEB1-   1.7648     4.7842 389.9992   0.369 0.712413
## treatmentb:conditionSHEB1+   4.5828     4.9229 389.9992   0.931 0.352473
## treatmentc:conditionSHEB1+   1.8496     4.9229 389.9992   0.376 0.707335
## treatmentd:conditionSHEB1+  -1.2337     4.9229 389.9992  -0.251 0.802253
## ---
## Signif. codes:  0 '***' 0.001 '**' 0.01 '*' 0.05 '.' 0.1 ' ' 1
```

```
##
## Correlation matrix not shown by default, as p = 16 > 12.
## Use print(x, correlation=TRUE)  or
##     vcov(x)        if you need it
```

```
summary(emmeans(m6, pairwise ~ condition|treatment, infer=T))
```

```
## Cannot use mode = "kenward-roger" because *pbkrtest* package is not installed
```

```
## $emmeans
## treatment = a:
##  condition emmean   SE   df lower.CL upper.CL t.ratio p.value
##  CTRL-      19.37 10.4 2.23   -21.36     60.1   1.858  0.1911
##  CTRL+      22.36 10.4 2.20   -18.67     63.4   2.152  0.1526
##  SHEB1-     15.79 10.4 2.20   -25.24     56.8   1.520  0.2570
##  SHEB1+     19.80 10.4 2.23   -20.93     60.5   1.900  0.1847
##
## treatment = b:
##  condition emmean   SE   df lower.CL upper.CL t.ratio p.value
##  CTRL-      55.04 10.4 2.23    14.32     95.8   5.282  0.0269
##  CTRL+      74.13 10.4 2.20    33.10    115.2   7.136  0.0147
##  SHEB1-     46.86 10.4 2.20     5.84     87.9   4.512  0.0383
##  SHEB1+     60.06 10.4 2.23    19.33    100.8   5.763  0.0224
##
## treatment = c:
##  condition emmean   SE   df lower.CL upper.CL t.ratio p.value
##  CTRL-      75.41 10.4 2.23    34.68    116.1   7.236  0.0137
##  CTRL+      98.15 10.4 2.20    57.12    139.2   9.448  0.0080
##  SHEB1-     65.21 10.4 2.20    24.18    106.2   6.277  0.0193
##  SHEB1+     77.69 10.4 2.23    36.96    118.4   7.455  0.0129
##
## treatment = d:
##  condition emmean   SE   df lower.CL upper.CL t.ratio p.value
##  CTRL-      11.56 10.4 2.23   -29.16     52.3   1.110  0.3725
##  CTRL+      10.74 10.4 2.20   -30.29     51.8   1.034  0.4012
##  SHEB1-      9.75 10.4 2.20   -31.28     50.8   0.939  0.4392
##  SHEB1+     10.77 10.4 2.23   -29.96     51.5   1.033  0.4005
```

```
##
## Degrees-of-freedom method: satterthwaite
## Confidence level used: 0.95
##
## $contrasts
## treatment = a:
##  contrast            estimate   SE  df lower.CL upper.CL t.ratio p.value
##  (CTRL-) - (CTRL+)    -2.9938 3.38 390  -11.727     5.74  -0.885  0.8129
##  (CTRL-) - (SHEB1-)    3.5795 3.38 390   -5.153    12.31   1.058  0.7155
##  (CTRL-) - (SHEB1+)   -0.4346 3.48 390   -9.416     8.55  -0.125  0.9993
##  (CTRL+) - (SHEB1-)    6.5733 3.28 390   -1.894    15.04   2.003  0.1886
##  (CTRL+) - (SHEB1+)    2.5591 3.38 390   -6.174    11.29   0.756  0.8740
##  (SHEB1-) - (SHEB1+)  -4.0142 3.38 390  -12.747     4.72  -1.186  0.6361
##
## treatment = b:
##  contrast            estimate   SE  df lower.CL upper.CL t.ratio p.value
##  (CTRL-) - (CTRL+)   -19.0865 3.38 390  -27.820   -10.35  -5.639  <.0001
##  (CTRL-) - (SHEB1-)    8.1757 3.38 390   -0.557    16.91   2.416  0.0758
##  (CTRL-) - (SHEB1+)   -5.0174 3.48 390  -13.999     3.96  -1.441  0.4743
##  (CTRL+) - (SHEB1-)   27.2623 3.28 390   18.794    35.73   8.307  <.0001
##  (CTRL+) - (SHEB1+)   14.0691 3.38 390    5.336    22.80   4.157  0.0002
##  (SHEB1-) - (SHEB1+) -13.1932 3.38 390  -21.926    -4.46  -3.898  0.0007
##
## treatment = c:
##  contrast            estimate   SE  df lower.CL upper.CL t.ratio p.value
##  (CTRL-) - (CTRL+)   -22.7432 3.38 390  -31.476   -14.01  -6.719  <.0001
##  (CTRL-) - (SHEB1-)   10.2005 3.38 390    1.467    18.93   3.014  0.0146
##  (CTRL-) - (SHEB1+)   -2.2842 3.48 390  -11.266     6.70  -0.656  0.9133
##  (CTRL+) - (SHEB1-)   32.9437 3.28 390   24.476    41.41  10.038  <.0001
##  (CTRL+) - (SHEB1+)   20.4590 3.38 390   11.726    29.19   6.045  <.0001
##  (SHEB1-) - (SHEB1+) -12.4847 3.38 390  -21.218    -3.75  -3.689  0.0015
##
## treatment = d:
##  contrast            estimate   SE  df lower.CL upper.CL t.ratio p.value
##  (CTRL-) - (CTRL+)     0.8229 3.38 390   -7.910     9.56   0.243  0.9949
##  (CTRL-) - (SHEB1-)    1.8147 3.38 390   -6.918    10.55   0.536  0.9502
##  (CTRL-) - (SHEB1+)    0.7990 3.48 390   -8.182     9.78   0.230  0.9957
##  (CTRL+) - (SHEB1-)    0.9918 3.28 390   -7.476     9.46   0.302  0.9904
##  (CTRL+) - (SHEB1+)   -0.0239 3.38 390   -8.757     8.71  -0.007  1.0000
##  (SHEB1-) - (SHEB1+)  -1.0157 3.38 390   -9.749     7.72  -0.300  0.9906
##
## Degrees-of-freedom method: satterthwaite
## Confidence level used: 0.95
## Conf-level adjustment: tukey method for comparing a family of 4 estimates
## P value adjustment: tukey method for comparing a family of 4 estimates
```

```r
emmip(m6, condition ~ treatment) +
  theme_bw()+
  xlab('Treatment') +
  ylab('Value')
```

```
## Cannot use mode = "kenward-roger" because *pbkrtest* package is not installed
```

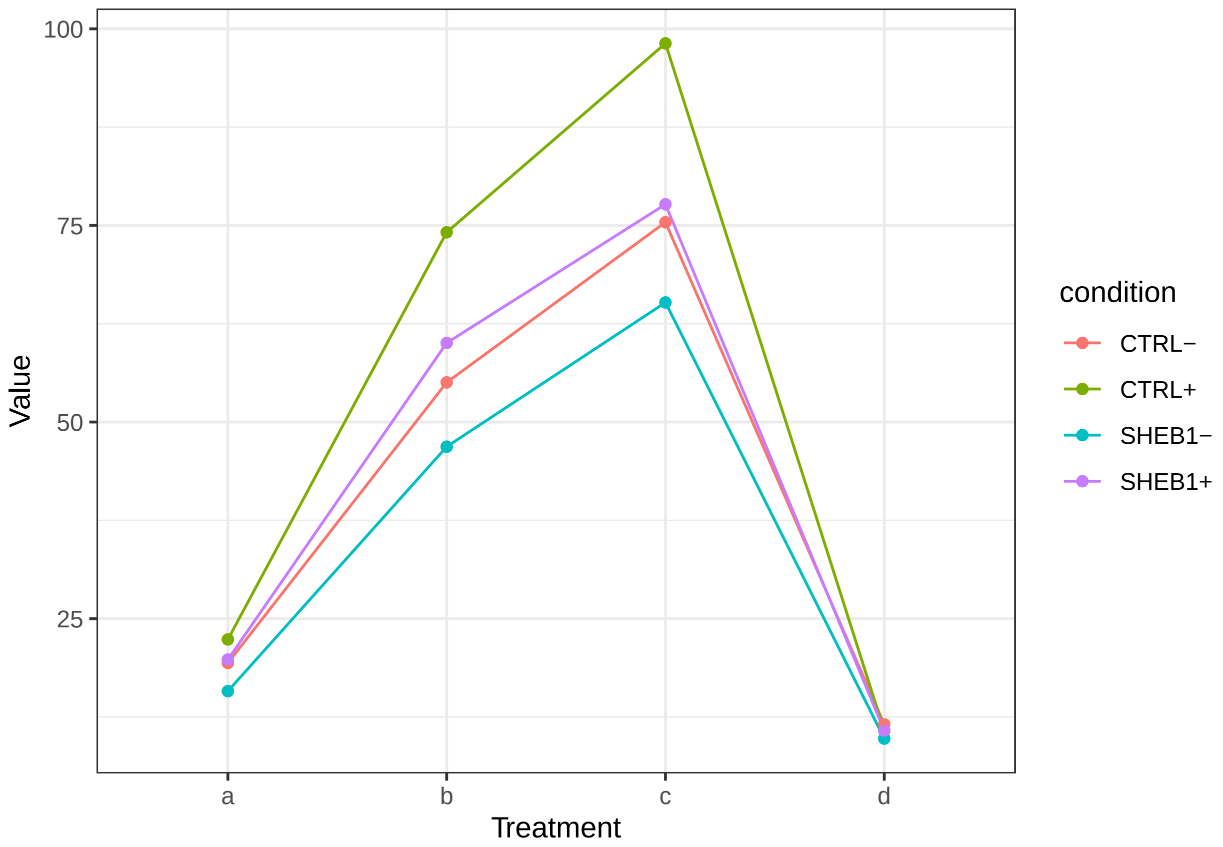


```
summary(pairs(emmeans(m6, "condition",by = "treatment",level = 0.95, infer = TRUE)))
```

```
## Cannot use mode = "kenward-roger" because *pbkrtest* package is not installed
```

```
## treatment = a:
##  contrast            estimate   SE  df t.ratio p.value
##  (CTRL-) - (CTRL+)    -2.9938 3.38 390  -0.885  0.8129
##  (CTRL-) - (SHEB1-)    3.5795 3.38 390   1.058  0.7155
##  (CTRL-) - (SHEB1+)   -0.4346 3.48 390  -0.125  0.9993
##  (CTRL+) - (SHEB1-)    6.5733 3.28 390   2.003  0.1886
##  (CTRL+) - (SHEB1+)    2.5591 3.38 390   0.756  0.8740
##  (SHEB1-) - (SHEB1+)  -4.0142 3.38 390  -1.186  0.6361
##
## treatment = b:
##  contrast            estimate   SE  df t.ratio p.value
##  (CTRL-) - (CTRL+)   -19.0865 3.38 390  -5.639  <.0001
##  (CTRL-) - (SHEB1-)    8.1757 3.38 390   2.416  0.0758
##  (CTRL-) - (SHEB1+)   -5.0174 3.48 390  -1.441  0.4743
##  (CTRL+) - (SHEB1-)   27.2623 3.28 390   8.307  <.0001
##  (CTRL+) - (SHEB1+)   14.0691 3.38 390   4.157  0.0002
##  (SHEB1-) - (SHEB1+) -13.1932 3.38 390  -3.898  0.0007
##
## treatment = c:
##  contrast            estimate   SE  df t.ratio p.value
##  (CTRL-) - (CTRL+)   -22.7432 3.38 390  -6.719  <.0001
```

```
##  (CTRL-) - (SHEB1-)   10.2005 3.38 390   3.014  0.0146
##  (CTRL-) - (SHEB1+)   -2.2842 3.48 390  -0.656  0.9133
##  (CTRL+) - (SHEB1-)   32.9437 3.28 390  10.038  <.0001
##  (CTRL+) - (SHEB1+)   20.4590 3.38 390   6.045  <.0001
##  (SHEB1-) - (SHEB1+) -12.4847 3.38 390  -3.689  0.0015
##
## treatment = d:
##  contrast            estimate   SE  df t.ratio p.value
##  (CTRL-) - (CTRL+)     0.8229 3.38 390   0.243  0.9949
##  (CTRL-) - (SHEB1-)    1.8147 3.38 390   0.536  0.9502
##  (CTRL-) - (SHEB1+)    0.7990 3.48 390   0.230  0.9957
##  (CTRL+) - (SHEB1-)    0.9918 3.28 390   0.302  0.9904
##  (CTRL+) - (SHEB1+)   -0.0239 3.38 390  -0.007  1.0000
##  (SHEB1-) - (SHEB1+)  -1.0157 3.38 390  -0.300  0.9906
##
## Degrees-of-freedom method: satterthwaite
## P value adjustment: tukey method for comparing a family of 4 estimates
```

MitoStress OCR - Lymphoblasts

```
ggplot(seahorse7, aes(time, value, colour=condition))+
  geom_point()+
  theme_bw()+
  xlab("Time (min)") +
  ylab('Value')+
  facet_wrap(~ind)
```

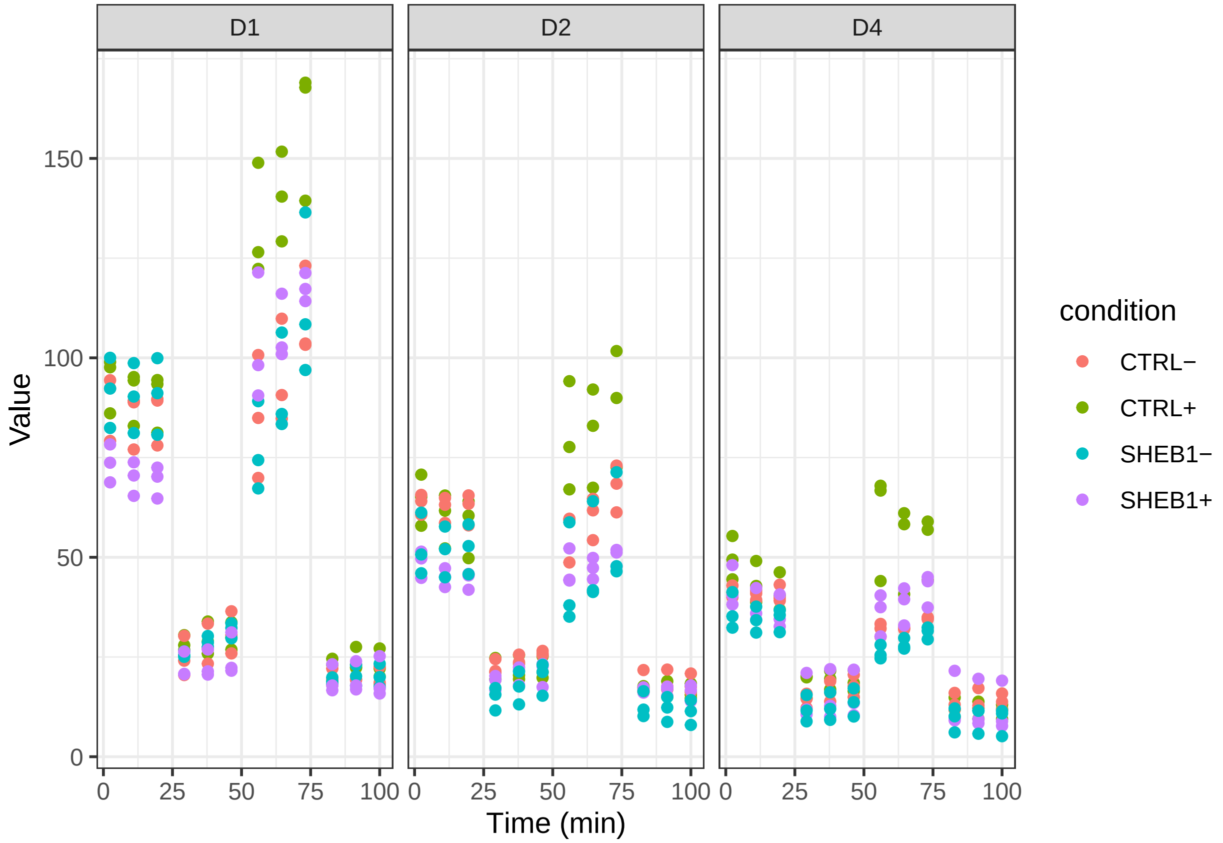


```
m7<-lmer(value ~ treatment*condition + (1|ind), data=seahorse7)
summary(m7)
```

```
## Linear mixed model fit by REML. t-tests use Satterthwaite's method [
## lmerModLmerTest]
## Formula: value ~ treatment * condition + (1 | ind)
##    Data: seahorse7
##
## REML criterion at convergence: 3405.2
##
## Scaled residuals:
##     Min      1Q  Median      3Q     Max
## -2.8339 -0.7952  0.1120  0.5584  4.4758
##
## Random effects:
##  Groups   Name        Variance Std.Dev.
##  ind      (Intercept) 305.6    17.48
##  Residual             180.3    13.43
## Number of obs: 432, groups:  ind, 3
##
## Fixed effects:
##                              Estimate Std. Error       df t value Pr(>|t|)
## (Intercept)                   63.3326    10.4182   2.2523   6.079  0.01942 *
## treatmentb                   -40.6075     3.6543 414.0000 -11.112  < 2e-16 ***
## treatmentc                     0.3034     3.6543 414.0000   0.083  0.93386
```

```
## treatmentd                  -45.8667     3.6543 414.0000 -12.551  < 2e-16 ***
## conditionCTRL+                2.4137     3.6543 414.0000   0.661  0.50930
## conditionSHEB1-              -4.0229     3.6543 414.0000  -1.101  0.27160
## conditionSHEB1+             -11.5162     3.6543 414.0000  -3.151  0.00174 **
## treatmentb:conditionCTRL+   -1.8503     5.1680 414.0000  -0.358  0.72051
## treatmentc:conditionCTRL+   28.0084     5.1680 414.0000   5.420 1.02e-07 ***
## treatmentd:conditionCTRL+   -2.6378     5.1680 414.0000  -0.510  0.61003
## treatmentb:conditionSHEB1-   0.8923     5.1680 414.0000   0.173  0.86301
## treatmentc:conditionSHEB1-  -2.2532     5.1680 414.0000  -0.436  0.66308
## treatmentd:conditionSHEB1-   0.3109     5.1680 414.0000   0.060  0.95205
## treatmentb:conditionSHEB1+   8.3169     5.1680 414.0000   1.609  0.10831
## treatmentc:conditionSHEB1+  13.2487     5.1680 414.0000   2.564  0.01071 *
## treatmentd:conditionSHEB1+  10.2159     5.1680 414.0000   1.977  0.04873 *
## ---
## Signif. codes:  0 '***' 0.001 '**' 0.01 '*' 0.05 '.' 0.1 ' ' 1

##
## Correlation matrix not shown by default, as p = 16 > 12.
## Use print(x, correlation=TRUE)  or
##     vcov(x)        if you need it
```

```
summary(emmeans(m7, pairwise ~ condition|treatment, infer=T))
```

```
## Cannot use mode = "kenward-roger" because *pbkrtest* package is not installed

## $emmeans
## treatment = a:
##  condition emmean   SE   df lower.CL upper.CL t.ratio p.value
##  CTRL-       63.3 10.4 2.25     23.0    103.7   6.079  0.0194
##  CTRL+       65.7 10.4 2.25     25.4    106.1   6.311  0.0179
##  SHEB1-      59.3 10.4 2.25     19.0     99.7   5.693  0.0224
##  SHEB1+      51.8 10.4 2.25     11.5     92.2   4.974  0.0298
##
## treatment = b:
##  condition emmean   SE   df lower.CL upper.CL t.ratio p.value
##  CTRL-       22.7 10.4 2.25    -17.6     63.1   2.181  0.1465
##  CTRL+       23.3 10.4 2.25    -17.1     63.6   2.235  0.1406
##  SHEB1-      19.6 10.4 2.25    -20.7     59.9   1.881  0.1863
##  SHEB1+      19.5 10.4 2.25    -20.8     59.9   1.874  0.1873
##
## treatment = c:
##  condition emmean   SE   df lower.CL upper.CL t.ratio p.value
##  CTRL-       63.6 10.4 2.25     23.3    104.0   6.108  0.0192
##  CTRL+       94.1 10.4 2.25     53.7    134.4   9.028  0.0082
##  SHEB1-      57.4 10.4 2.25     17.0     97.7   5.506  0.0240
##  SHEB1+      65.4 10.4 2.25     25.0    105.7   6.274  0.0181
##
## treatment = d:
##  condition emmean   SE   df lower.CL upper.CL t.ratio p.value
##  CTRL-       17.5 10.4 2.25    -22.9     57.8   1.676  0.2215
##  CTRL+       17.2 10.4 2.25    -23.1     57.6   1.655  0.2257
##  SHEB1-      13.8 10.4 2.25    -26.6     54.1   1.320  0.3049
##  SHEB1+      16.2 10.4 2.25    -24.2     56.5   1.552  0.2472
```

```
##
## Degrees-of-freedom method: satterthwaite
## Confidence level used: 0.95
##
## $contrasts
## treatment = a:
##  contrast            estimate   SE  df lower.CL upper.CL t.ratio p.value
##  (CTRL-) - (CTRL+)    -2.4137 3.65 414   -11.84     7.01  -0.661  0.9118
##  (CTRL-) - (SHEB1-)    4.0229 3.65 414    -5.40    13.45   1.101  0.6892
##  (CTRL-) - (SHEB1+)   11.5162 3.65 414     2.09    20.94   3.151  0.0094
##  (CTRL+) - (SHEB1-)    6.4366 3.65 414    -2.99    15.86   1.761  0.2936
##  (CTRL+) - (SHEB1+)   13.9299 3.65 414     4.50    23.36   3.812  0.0009
##  (SHEB1-) - (SHEB1+)   7.4932 3.65 414    -1.93    16.92   2.051  0.1714
##
## treatment = b:
##  contrast            estimate   SE  df lower.CL upper.CL t.ratio p.value
##  (CTRL-) - (CTRL+)    -0.5635 3.65 414    -9.99     8.86  -0.154  0.9987
##  (CTRL-) - (SHEB1-)    3.1307 3.65 414    -6.30    12.56   0.857  0.8271
##  (CTRL-) - (SHEB1+)    3.1992 3.65 414    -6.23    12.63   0.875  0.8176
##  (CTRL+) - (SHEB1-)    3.6941 3.65 414    -5.73    13.12   1.011  0.7432
##  (CTRL+) - (SHEB1+)    3.7627 3.65 414    -5.66    13.19   1.030  0.7321
##  (SHEB1-) - (SHEB1+)   0.0686 3.65 414    -9.36     9.49   0.019  1.0000
##
## treatment = c:
##  contrast            estimate   SE  df lower.CL upper.CL t.ratio p.value
##  (CTRL-) - (CTRL+)   -30.4221 3.65 414   -39.85   -21.00  -8.325  <.0001
##  (CTRL-) - (SHEB1-)    6.2761 3.65 414    -3.15    15.70   1.717  0.3159
##  (CTRL-) - (SHEB1+)   -1.7325 3.65 414   -11.16     7.69  -0.474  0.9648
##  (CTRL+) - (SHEB1-)   36.6981 3.65 414    27.27    46.12  10.042  <.0001
##  (CTRL+) - (SHEB1+)   28.6896 3.65 414    19.26    38.12   7.851  <.0001
##  (SHEB1-) - (SHEB1+)  -8.0086 3.65 414   -17.43     1.42  -2.192  0.1273
##
## treatment = d:
##  contrast            estimate   SE  df lower.CL upper.CL t.ratio p.value
##  (CTRL-) - (CTRL+)     0.2241 3.65 414    -9.20     9.65   0.061  0.9999
##  (CTRL-) - (SHEB1-)    3.7120 3.65 414    -5.71    13.14   1.016  0.7403
##  (CTRL-) - (SHEB1+)    1.3002 3.65 414    -8.13    10.73   0.356  0.9845
##  (CTRL+) - (SHEB1-)    3.4878 3.65 414    -5.94    12.91   0.954  0.7753
##  (CTRL+) - (SHEB1+)    1.0761 3.65 414    -8.35    10.50   0.294  0.9911
##  (SHEB1-) - (SHEB1+)  -2.4117 3.65 414   -11.84     7.01  -0.660  0.9120
##
## Degrees-of-freedom method: satterthwaite
## Confidence level used: 0.95
## Conf-level adjustment: tukey method for comparing a family of 4 estimates
## P value adjustment: tukey method for comparing a family of 4 estimates
```

```r
emmip(m7, condition ~ treatment) +
  theme_bw()+
  xlab('Treatment') +
  ylab('Value')
```

```
## Cannot use mode = "kenward-roger" because *pbkrtest* package is not installed
```

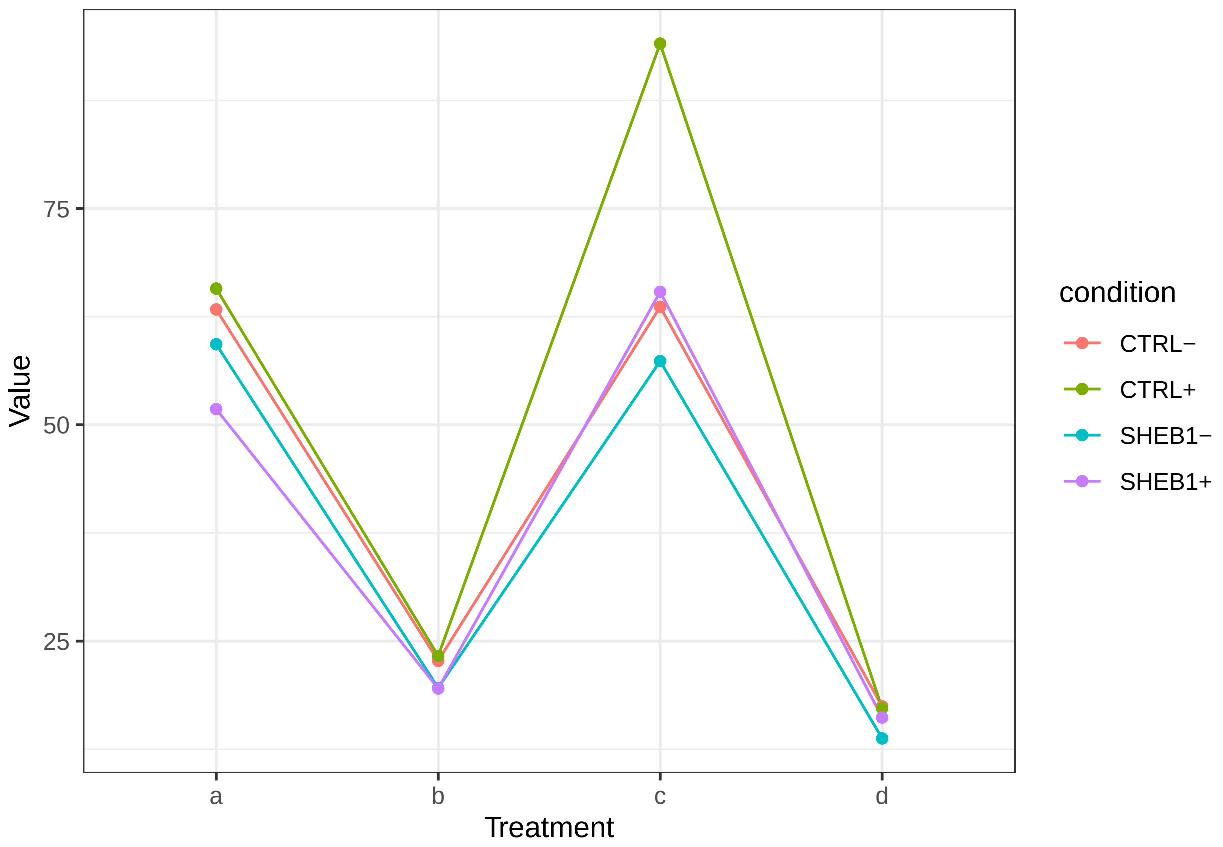


```
summary(pairs(emmeans(m7, "condition",by = "treatment",level = 0.95, infer = TRUE)))
```

```
## Cannot use mode = "kenward-roger" because *pbkrtest* package is not installed
```

```
## treatment = a:
##  contrast            estimate   SE  df t.ratio p.value
##  (CTRL-) - (CTRL+)    -2.4137 3.65 414  -0.661  0.9118
##  (CTRL-) - (SHEB1-)    4.0229 3.65 414   1.101  0.6892
##  (CTRL-) - (SHEB1+)   11.5162 3.65 414   3.151  0.0094
##  (CTRL+) - (SHEB1-)    6.4366 3.65 414   1.761  0.2936
##  (CTRL+) - (SHEB1+)   13.9299 3.65 414   3.812  0.0009
##  (SHEB1-) - (SHEB1+)   7.4932 3.65 414   2.051  0.1714
##
## treatment = b:
##  contrast            estimate   SE  df t.ratio p.value
##  (CTRL-) - (CTRL+)    -0.5635 3.65 414  -0.154  0.9987
##  (CTRL-) - (SHEB1-)    3.1307 3.65 414   0.857  0.8271
##  (CTRL-) - (SHEB1+)    3.1992 3.65 414   0.875  0.8176
##  (CTRL+) - (SHEB1-)    3.6941 3.65 414   1.011  0.7432
##  (CTRL+) - (SHEB1+)    3.7627 3.65 414   1.030  0.7321
##  (SHEB1-) - (SHEB1+)   0.0686 3.65 414   0.019  1.0000
##
## treatment = c:
##  contrast            estimate   SE  df t.ratio p.value
##  (CTRL-) - (CTRL+)   -30.4221 3.65 414  -8.325  <.0001
```

```
##  (CTRL-) - (SHEB1-)    6.2761 3.65 414   1.717  0.3159
##  (CTRL-) - (SHEB1+)   -1.7325 3.65 414  -0.474  0.9648
##  (CTRL+) - (SHEB1-)   36.6981 3.65 414  10.042  <.0001
##  (CTRL+) - (SHEB1+)   28.6896 3.65 414   7.851  <.0001
##  (SHEB1-) - (SHEB1+)  -8.0086 3.65 414  -2.192  0.1273
##
## treatment = d:
##  contrast            estimate   SE  df t.ratio p.value
##  (CTRL-) - (CTRL+)     0.2241 3.65 414   0.061  0.9999
##  (CTRL-) - (SHEB1-)    3.7120 3.65 414   1.016  0.7403
##  (CTRL-) - (SHEB1+)    1.3002 3.65 414   0.356  0.9845
##  (CTRL+) - (SHEB1-)    3.4878 3.65 414   0.954  0.7753
##  (CTRL+) - (SHEB1+)    1.0761 3.65 414   0.294  0.9911
##  (SHEB1-) - (SHEB1+)  -2.4117 3.65 414  -0.660  0.9120
##
## Degrees-of-freedom method: satterthwaite
## P value adjustment: tukey method for comparing a family of 4 estimates
```

MitoStress ECAR - Lymphoblasts

```
ggplot(seahorse8, aes(time, value, colour=condition))+
  geom_point()+
  theme_bw()+
  xlab("Time (min)") +
  ylab('Value')+
  facet_wrap(~ind)
```

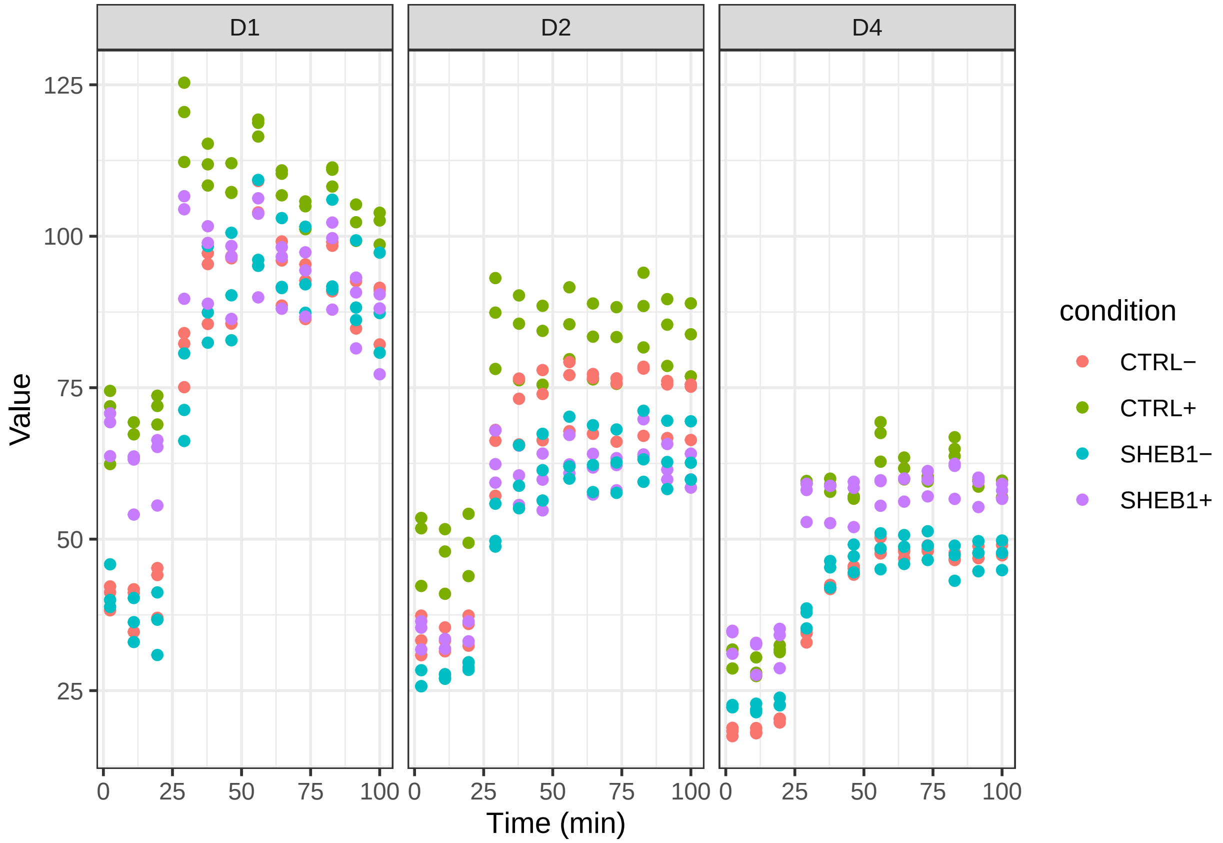


```
m8<-lmer(value ~ treatment*condition + (1|ind), data=seahorse8)
summary(m8)
```

```
## Linear mixed model fit by REML. t-tests use Satterthwaite's method [
## lmerModLmerTest]
## Formula: value ~ treatment * condition + (1 | ind)
##    Data: seahorse8
##
## REML criterion at convergence: 2892.2
##
## Scaled residuals:
##      Min       1Q   Median       3Q      Max
## -2.75001 -0.75368 -0.04085  0.76579  2.58247
##
## Random effects:
##  Groups   Name        Variance Std.Dev.
##  ind      (Intercept) 401.94   20.048
##  Residual              52.15    7.221
## Number of obs: 432, groups:  ind, 3
##
## Fixed effects:
##                             Estimate Std. Error      df t value Pr(>|t|)
## (Intercept)                   31.226     11.658   2.054   2.678   0.1125
## treatmentb                    34.931      1.965 414.000  17.773  < 2e-16 ***
## treatmentc                    41.544      1.965 414.000  21.138  < 2e-16 ***
```

```
## treatmentd                    39.551      1.965 414.000  20.124  < 2e-16 ***
## conditionCTRL+                18.134      1.965 414.000   9.227  < 2e-16 ***
## conditionSHEB1-               -1.823      1.965 414.000  -0.928   0.3541
## conditionSHEB1+               11.716      1.965 414.000   5.961 5.37e-09 ***
## treatmentb:conditionCTRL+      1.068      2.779 414.000   0.384   0.7010
## treatmentc:conditionCTRL+     -5.290      2.779 414.000  -1.903   0.0577 .
## treatmentd:conditionCTRL+     -5.330      2.779 414.000  -1.918   0.0558 .
## treatmentb:conditionSHEB1-    -2.659      2.779 414.000  -0.957   0.3393
## treatmentc:conditionSHEB1-    -1.555      2.779 414.000  -0.560   0.5761
## treatmentd:conditionSHEB1-    -1.240      2.779 414.000  -0.446   0.6557
## treatmentb:conditionSHEB1+    -6.328      2.779 414.000  -2.277   0.0233 *
## treatmentc:conditionSHEB1+   -12.352      2.779 414.000  -4.444 1.14e-05 ***
## treatmentd:conditionSHEB1+   -11.836      2.779 414.000  -4.258 2.55e-05 ***
## ---
## Signif. codes:  0 '***' 0.001 '**' 0.01 '*' 0.05 '.' 0.1 ' ' 1
```

```
##
## Correlation matrix not shown by default, as p = 16 > 12.
## Use print(x, correlation=TRUE)  or
##     vcov(x)        if you need it
```

```
summary(emmeans(m8, pairwise ~ condition|treatment, infer=T))
```

```
## Cannot use mode = "kenward-roger" because *pbkrtest* package is not installed
```

```
## $emmeans
## treatment = a:
##  condition emmean   SE   df lower.CL upper.CL t.ratio p.value
##  CTRL-       31.2 11.7 2.05   -17.68     80.1   2.678  0.1125
##  CTRL+       49.4 11.7 2.05     0.45     98.3   4.234  0.0491
##  SHEB1-      29.4 11.7 2.05   -19.51     78.3   2.522  0.1245
##  SHEB1+      42.9 11.7 2.05    -5.97     91.9   3.683  0.0638
##
## treatment = b:
##  condition emmean   SE   df lower.CL upper.CL t.ratio p.value
##  CTRL-       66.2 11.7 2.05    17.25    115.1   5.675  0.0279
##  CTRL+       85.4 11.7 2.05    36.45    134.3   7.322  0.0169
##  SHEB1-      61.7 11.7 2.05    12.76    110.6   5.290  0.0320
##  SHEB1+      71.5 11.7 2.05    22.63    120.5   6.137  0.0239
##
## treatment = c:
##  condition emmean   SE   df lower.CL upper.CL t.ratio p.value
##  CTRL-       72.8 11.7 2.05    23.86    121.7   6.242  0.0231
##  CTRL+       85.6 11.7 2.05    36.70    134.5   7.344  0.0168
##  SHEB1-      69.4 11.7 2.05    20.48    118.3   5.952  0.0254
##  SHEB1+      72.1 11.7 2.05    23.22    121.0   6.187  0.0235
##
## treatment = d:
##  condition emmean   SE   df lower.CL upper.CL t.ratio p.value
##  CTRL-       70.8 11.7 2.05    21.87    119.7   6.071  0.0244
##  CTRL+       83.6 11.7 2.05    34.67    132.5   7.169  0.0176
##  SHEB1-      67.7 11.7 2.05    18.80    116.6   5.808  0.0267
##  SHEB1+      70.7 11.7 2.05    21.75    119.6   6.061  0.0245
```

```
##
## Degrees-of-freedom method: satterthwaite
## Confidence level used: 0.95
##
## $contrasts
## treatment = a:
##  contrast            estimate   SE  df lower.CL upper.CL t.ratio p.value
##  (CTRL-) - (CTRL+)    -18.134 1.97 414  -23.204  -13.064  -9.227  <.0001
##  (CTRL-) - (SHEB1-)     1.823 1.97 414   -3.246    6.893   0.928  0.7900
##  (CTRL-) - (SHEB1+)   -11.716 1.97 414  -16.786   -6.646  -5.961  <.0001
##  (CTRL+) - (SHEB1-)    19.957 1.97 414   14.888   25.027  10.154  <.0001
##  (CTRL+) - (SHEB1+)     6.418 1.97 414    1.348   11.488   3.266  0.0065
##  (SHEB1-) - (SHEB1+)  -13.539 1.97 414  -18.609   -8.470  -6.889  <.0001
##
## treatment = b:
##  contrast            estimate   SE  df lower.CL upper.CL t.ratio p.value
##  (CTRL-) - (CTRL+)    -19.202 1.97 414  -24.272  -14.132  -9.770  <.0001
##  (CTRL-) - (SHEB1-)     4.482 1.97 414   -0.587    9.552   2.281  0.1042
##  (CTRL-) - (SHEB1+)    -5.388 1.97 414  -10.458   -0.318  -2.741  0.0323
##  (CTRL+) - (SHEB1-)    23.684 1.97 414   18.615   28.754  12.051  <.0001
##  (CTRL+) - (SHEB1+)    13.814 1.97 414    8.744   18.884   7.029  <.0001
##  (SHEB1-) - (SHEB1+)   -9.870 1.97 414  -14.940   -4.801  -5.022  <.0001
##
## treatment = c:
##  contrast            estimate   SE  df lower.CL upper.CL t.ratio p.value
##  (CTRL-) - (CTRL+)    -12.844 1.97 414  -17.914   -7.775  -6.535  <.0001
##  (CTRL-) - (SHEB1-)     3.378 1.97 414   -1.691    8.448   1.719  0.3151
##  (CTRL-) - (SHEB1+)     0.636 1.97 414   -4.434    5.705   0.323  0.9883
##  (CTRL+) - (SHEB1-)    16.223 1.97 414   11.153   21.292   8.254  <.0001
##  (CTRL+) - (SHEB1+)    13.480 1.97 414    8.410   18.550   6.859  <.0001
##  (SHEB1-) - (SHEB1+)   -2.743 1.97 414   -7.812    2.327  -1.396  0.5029
##
## treatment = d:
##  contrast            estimate   SE  df lower.CL upper.CL t.ratio p.value
##  (CTRL-) - (CTRL+)    -12.804 1.97 414  -17.873   -7.734  -6.515  <.0001
##  (CTRL-) - (SHEB1-)     3.063 1.97 414   -2.006    8.133   1.559  0.4036
##  (CTRL-) - (SHEB1+)     0.120 1.97 414   -4.950    5.189   0.061  0.9999
##  (CTRL+) - (SHEB1-)    15.867 1.97 414   10.797   20.937   8.073  <.0001
##  (CTRL+) - (SHEB1+)    12.923 1.97 414    7.854   17.993   6.576  <.0001
##  (SHEB1-) - (SHEB1+)   -2.944 1.97 414   -8.013    2.126  -1.498  0.4398
##
## Degrees-of-freedom method: satterthwaite
## Confidence level used: 0.95
## Conf-level adjustment: tukey method for comparing a family of 4 estimates
## P value adjustment: tukey method for comparing a family of 4 estimates
```

```r
emmip(m8, condition ~ treatment) +
  theme_bw()+
  xlab('Treatment') +
  ylab('Value')
```

```
## Cannot use mode = "kenward-roger" because *pbkrtest* package is not installed
```

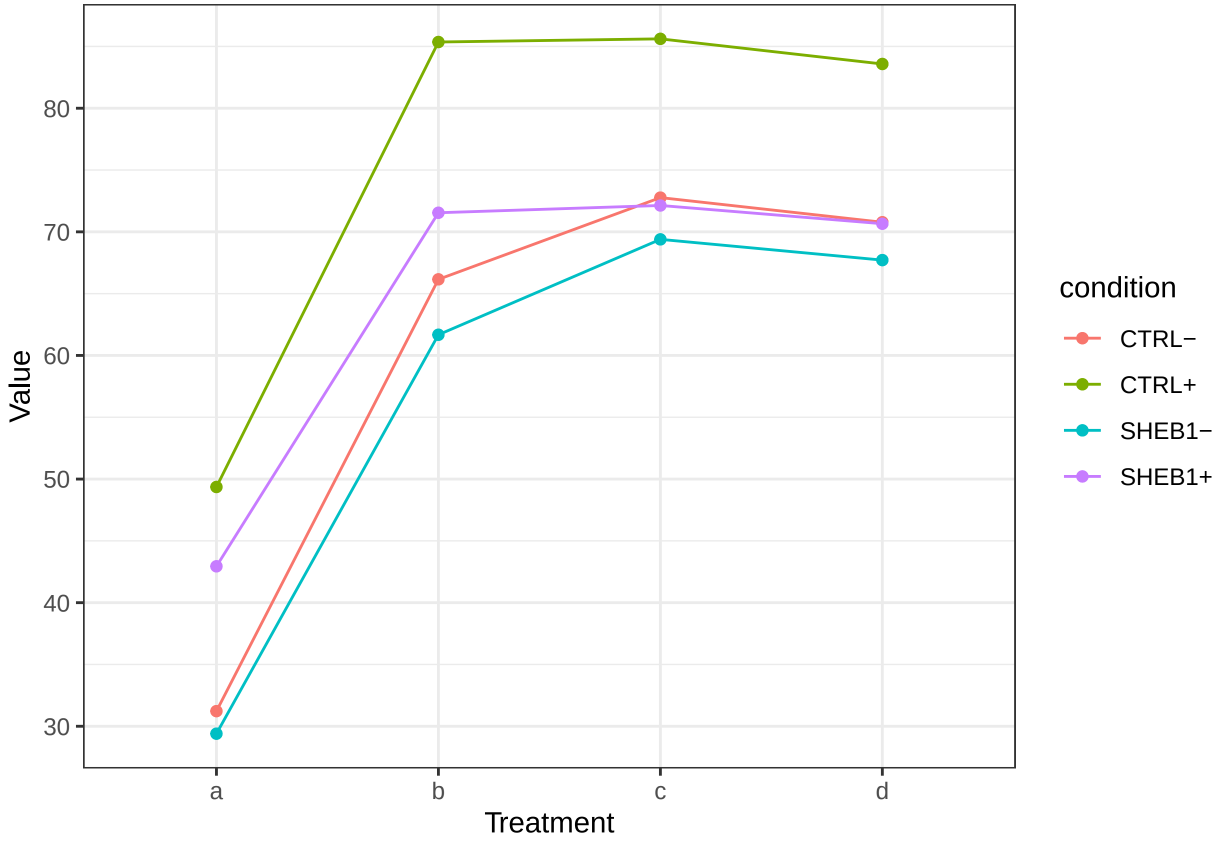


```
summary(pairs(emmeans(m8, "condition",by = "treatment",level = 0.95, infer = TRUE)))
```

```
## Cannot use mode = "kenward-roger" because *pbkrtest* package is not installed
```

```
## treatment = a:
##  contrast            estimate   SE  df t.ratio p.value
##  (CTRL-) - (CTRL+)    -18.134 1.97 414  -9.227  <.0001
##  (CTRL-) - (SHEB1-)     1.823 1.97 414   0.928  0.7900
##  (CTRL-) - (SHEB1+)   -11.716 1.97 414  -5.961  <.0001
##  (CTRL+) - (SHEB1-)    19.957 1.97 414  10.154  <.0001
##  (CTRL+) - (SHEB1+)     6.418 1.97 414   3.266  0.0065
##  (SHEB1-) - (SHEB1+)  -13.539 1.97 414  -6.889  <.0001
##
## treatment = b:
##  contrast            estimate   SE  df t.ratio p.value
##  (CTRL-) - (CTRL+)    -19.202 1.97 414  -9.770  <.0001
##  (CTRL-) - (SHEB1-)     4.482 1.97 414   2.281  0.1042
##  (CTRL-) - (SHEB1+)    -5.388 1.97 414  -2.741  0.0323
##  (CTRL+) - (SHEB1-)    23.684 1.97 414  12.051  <.0001
##  (CTRL+) - (SHEB1+)    13.814 1.97 414   7.029  <.0001
##  (SHEB1-) - (SHEB1+)   -9.870 1.97 414  -5.022  <.0001
##
## treatment = c:
##  contrast            estimate   SE  df t.ratio p.value
##  (CTRL-) - (CTRL+)    -12.844 1.97 414  -6.535  <.0001
```

```
##  (CTRL-) - (SHEB1-)     3.378 1.97 414   1.719  0.3151
##  (CTRL-) - (SHEB1+)     0.636 1.97 414   0.323  0.9883
##  (CTRL+) - (SHEB1-)    16.223 1.97 414   8.254  <.0001
##  (CTRL+) - (SHEB1+)    13.480 1.97 414   6.859  <.0001
##  (SHEB1-) - (SHEB1+)   -2.743 1.97 414  -1.396  0.5029
##
## treatment = d:
##  contrast            estimate   SE  df t.ratio p.value
##  (CTRL-) - (CTRL+)    -12.804 1.97 414  -6.515  <.0001
##  (CTRL-) - (SHEB1-)     3.063 1.97 414   1.559  0.4036
##  (CTRL-) - (SHEB1+)     0.120 1.97 414   0.061  0.9999
##  (CTRL+) - (SHEB1-)    15.867 1.97 414   8.073  <.0001
##  (CTRL+) - (SHEB1+)    12.923 1.97 414   6.576  <.0001
##  (SHEB1-) - (SHEB1+)   -2.944 1.97 414  -1.498  0.4398
##
## Degrees-of-freedom method: satterthwaite
## P value adjustment: tukey method for comparing a family of 4 estimates
```
