# Supplementary figures and images for: End-binding protein 1 regulates the metabolic fate of CD4+ T lymphoblasts and Jurkat T cells and the organization of the mitochondrial network
Source: Front Immunol. 2023 Jul 13;14:1197289. doi: 10.3389/fimmu.2023.1197289 (PMC10374013; doi:10.3389/fimmu.2023.1197289)

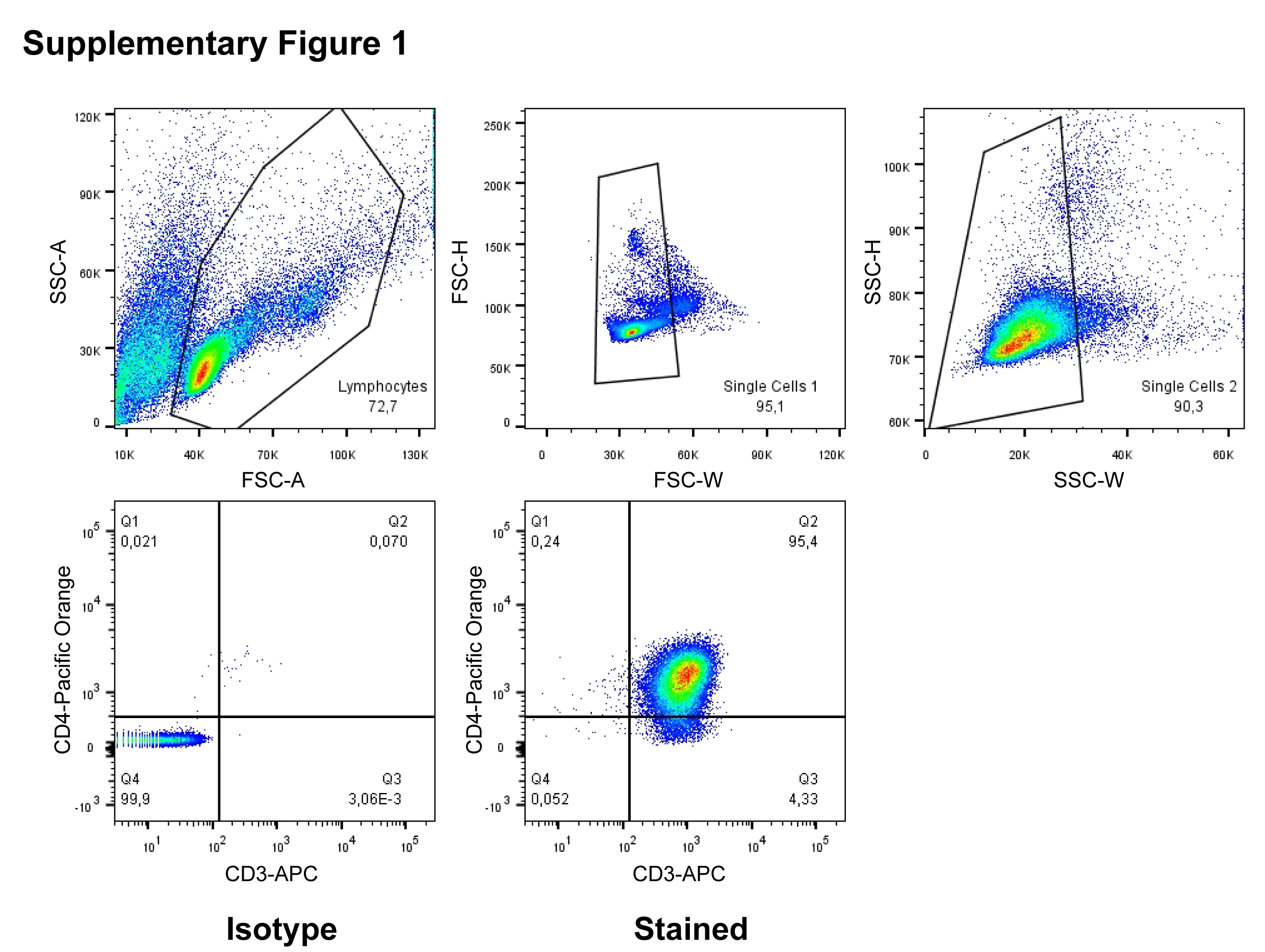

Supplement: Supplementary Figure 1 — Gating strategy used to assess purity of CD4+ T lymphoblasts in terms of CD3 and CD4 expression. Corresponds to Figures 1 – 7 . [file Image_1.tif]

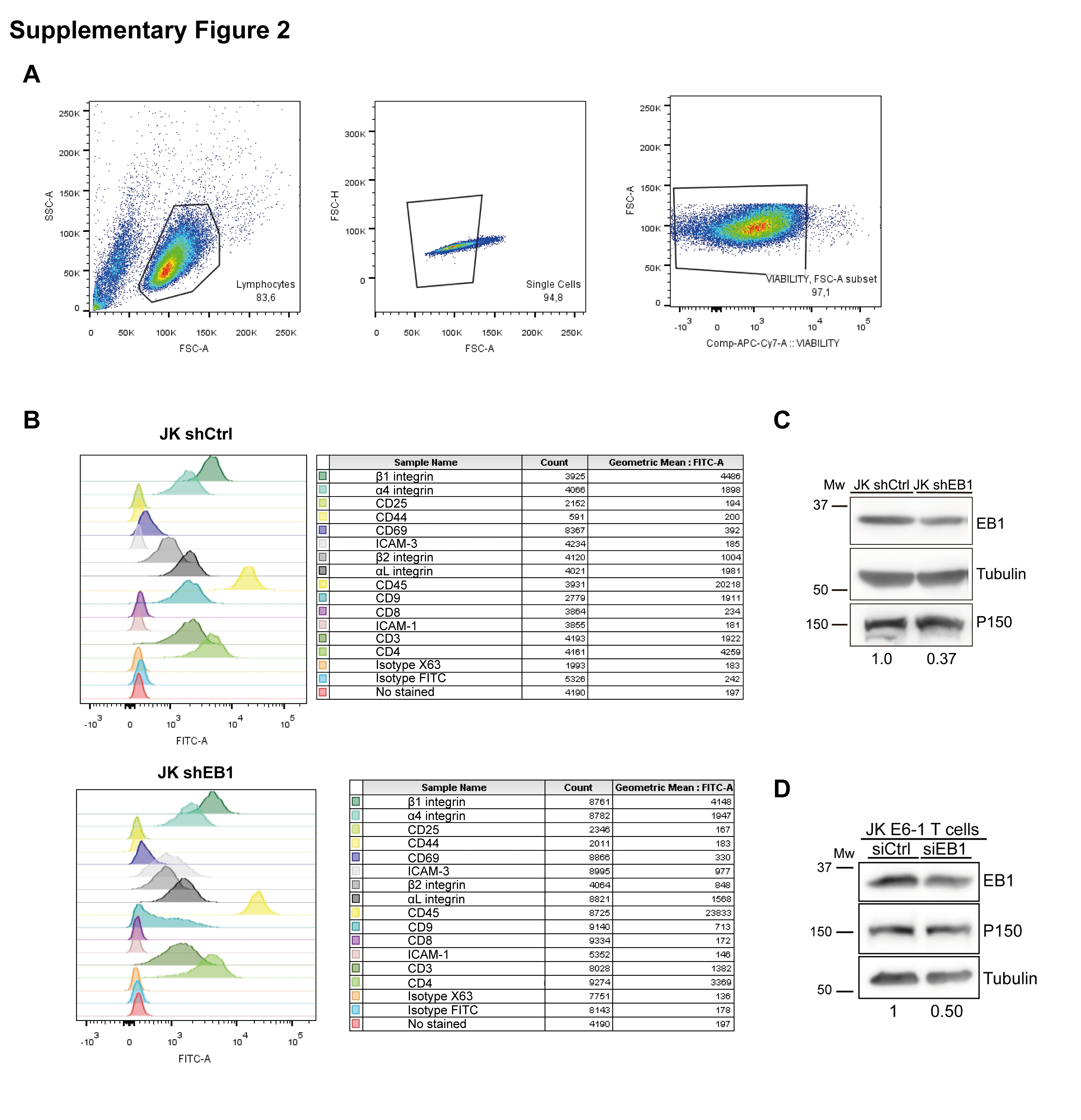

Supplement: Supplementary Figure 2 — Expression of receptors in shCtrl and shEB1 Jurkat E6-1 T cell clones. (A) Gating strategy. (B) Graph, geometric mean of the fluorescence intensity (GeoMFI) of indicated surface markers of shCtrl and shEB1 Jurkat T cells. (C) Western blot showing EB1 silencing in shEB1 Jurkat E6-1 T cells. α-tubulin and p150Glued were used as loading controls. (D) Western blot showing EB1 silencing in siEB1 Jurkat E6-1 T cells. α-tubulin and p150Glued were used as loading controls. Corresponds to Figure 3. [file Image_2.tif]

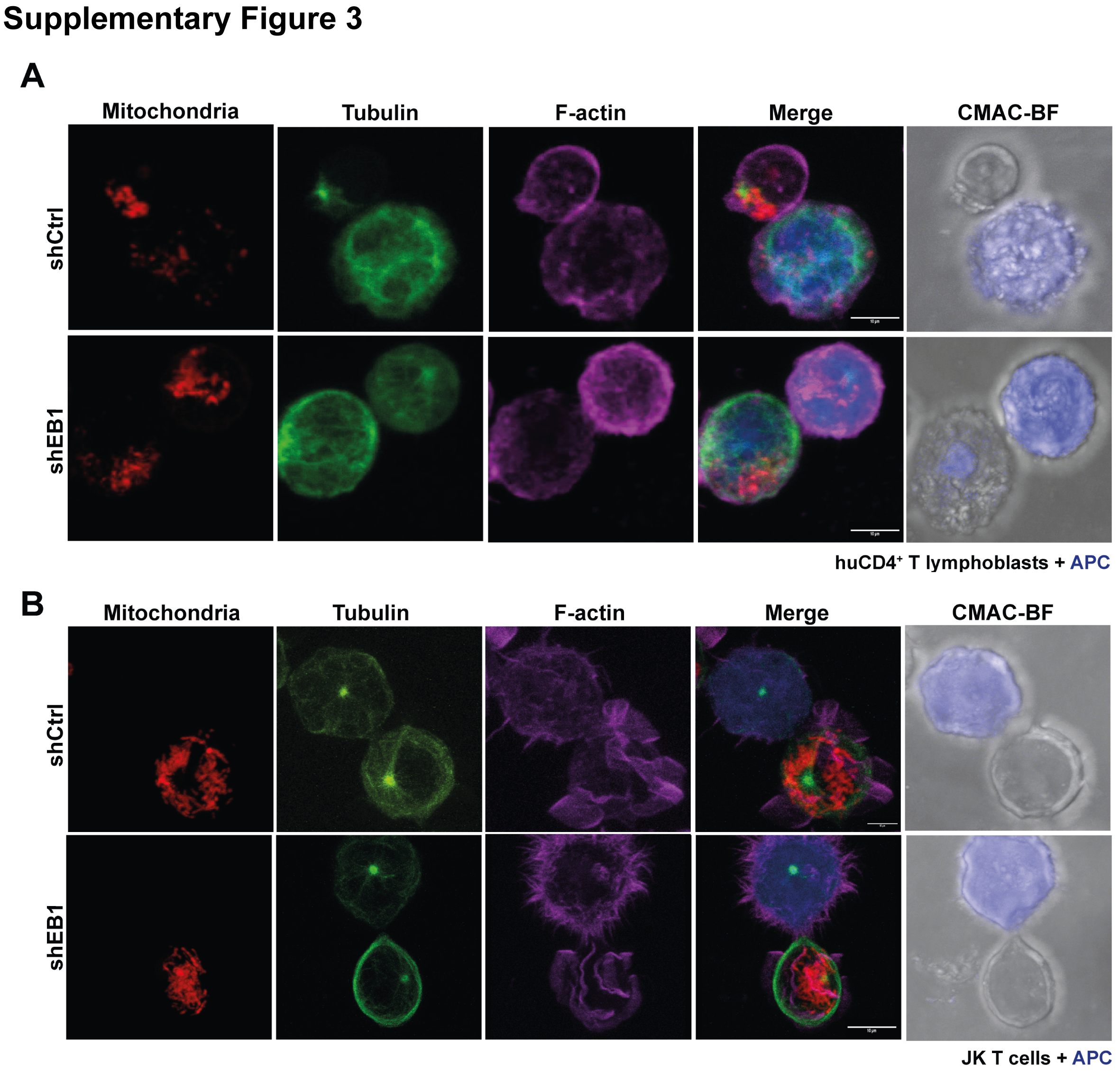

Supplement: Supplementary Figure 3 — Confocal images of cell conjugates formed between shCtrl and shEB1 (A) huCD4+ T lymphoblasts and (B) Jurkat E6-1 T cells with unloaded Raji cells (APCs). Red, mitochondria; green, α-tubulin; magenta, F-actin; blue, CMAC (Raji cell). Maximal projections are shown. Bar, 10 μm. Corresponds to Figure 3. [file Image_3.tif]

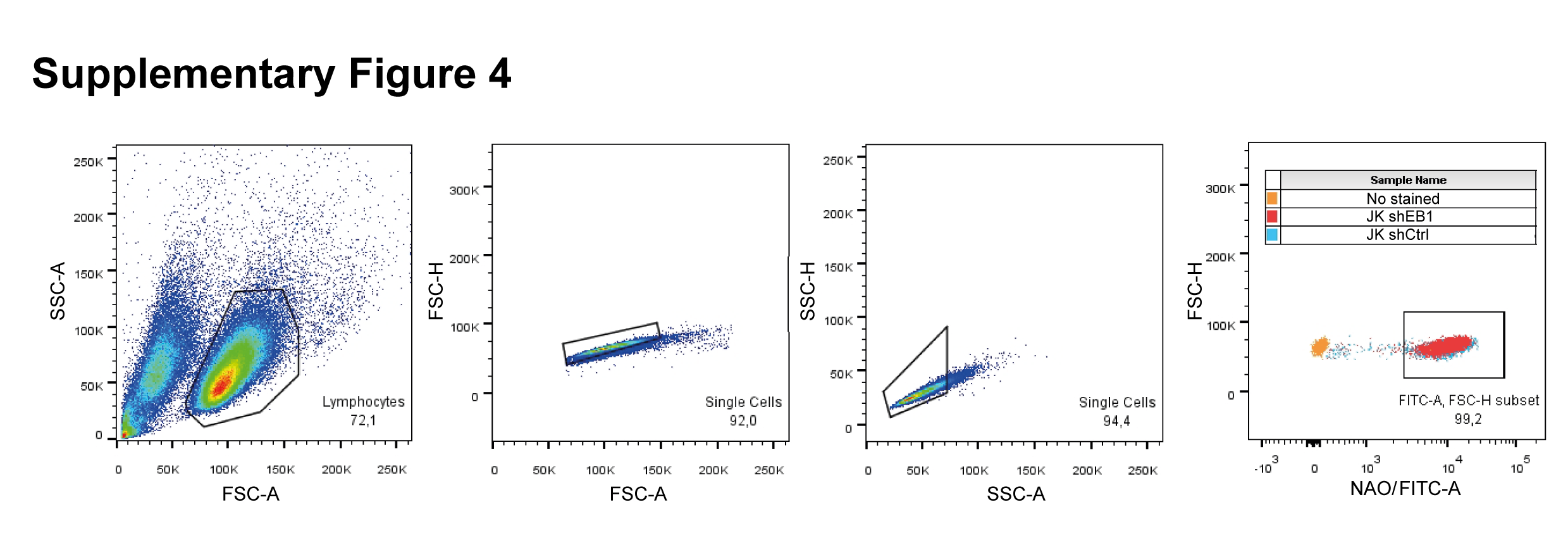

Supplement: Supplementary Figure 4 — Gating strategy used to measure mitochondrial mass with Nonyl acridine orange (NAO). Corresponds to Figure 6. [file Image_4.tif]

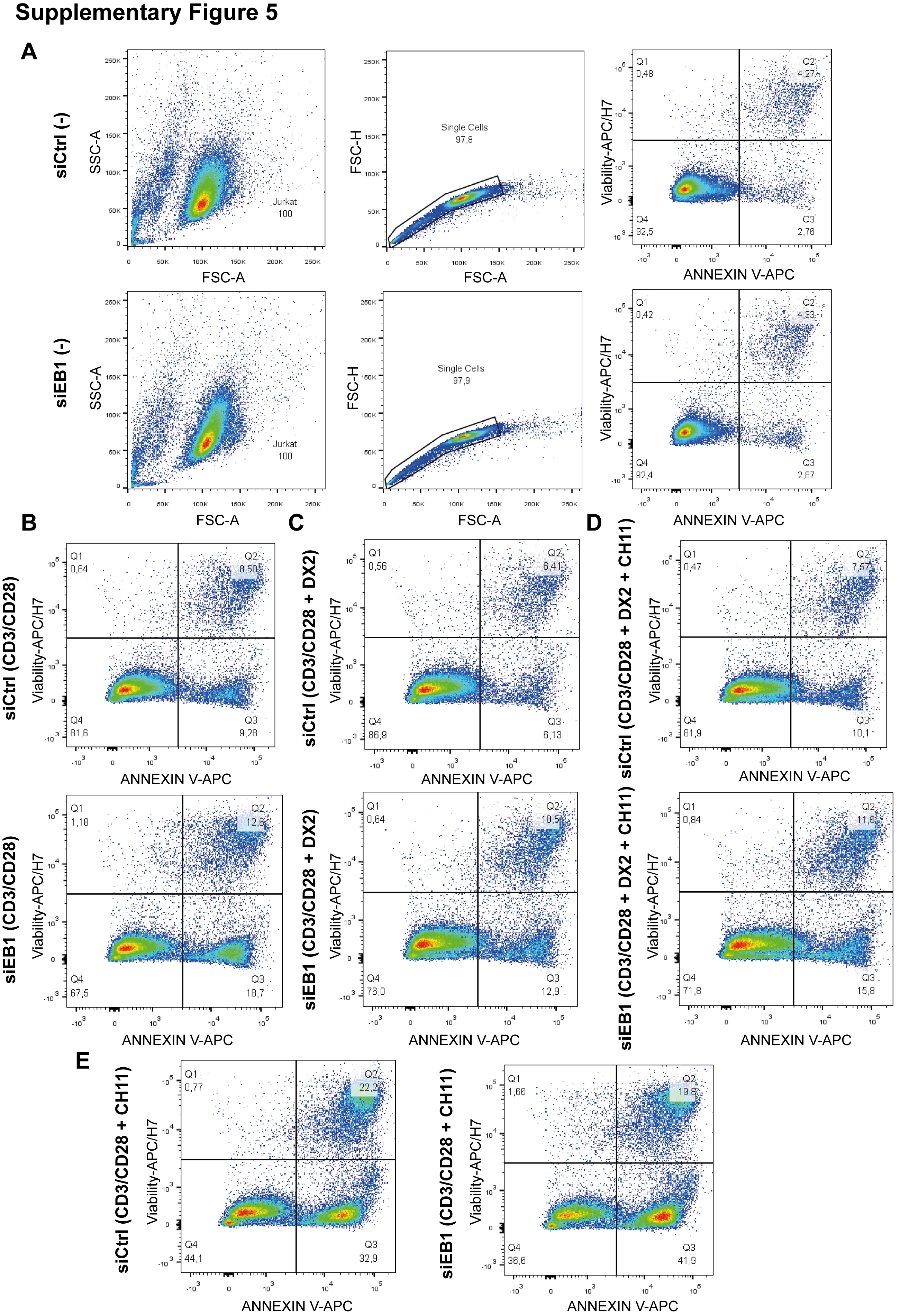

Supplement: Supplementary Figure 5 — Gating strategy used for cell viability analysis. (A) Example of the gating strategy for non-activated control (siCtrl; upper panel) and EB1-silenced cells (siEB1; bottom panel) by siRNAs. The left panels show the whole population selected for the cell viability analysis, which included dead and live cells. In the central panel doublets were excluded. In the right panels, the dot plots show the staining of APC-labelled Annexin V and Ghost dye red 780. Annexin- Ghost dye+ (Q1) represent necrotic cells, Annexin+ Ghost dye+ (Q2) are late apoptotic cells, Annexin+ Ghost dye- (Q3) indicates early apoptosis and Annexin- Ghost dye- (Q1) are live cells. (B) Dot plots of Annexin and Ghost Dye red 780 in siCtrl and siEB1 cells after overnight incubation with αCD3 and αCD28. (C) Dot plots of Annexin and Ghost Dye red 780 in siCtrl and siEB1 cells after overnight incubation with anti-Fas antagonistic antibody DX2 and activation with αCD3 and αCD28. (D) Dot plots of Annexin and Ghost Dye red 780 in siCtrl and siEB1 cells after overnight incubation with DX2, activation with αCD3 and αCD28 and the apoptotic inducer CH-11. (E) Dot plots of Annexin and Ghost Dye red 780 in siCtrl and siEB1 cells after overnight incubation with αCD3, αCD28 and the apoptotic inducer CH-11. Corresponds to Figure 7. [file Image_5.tif]

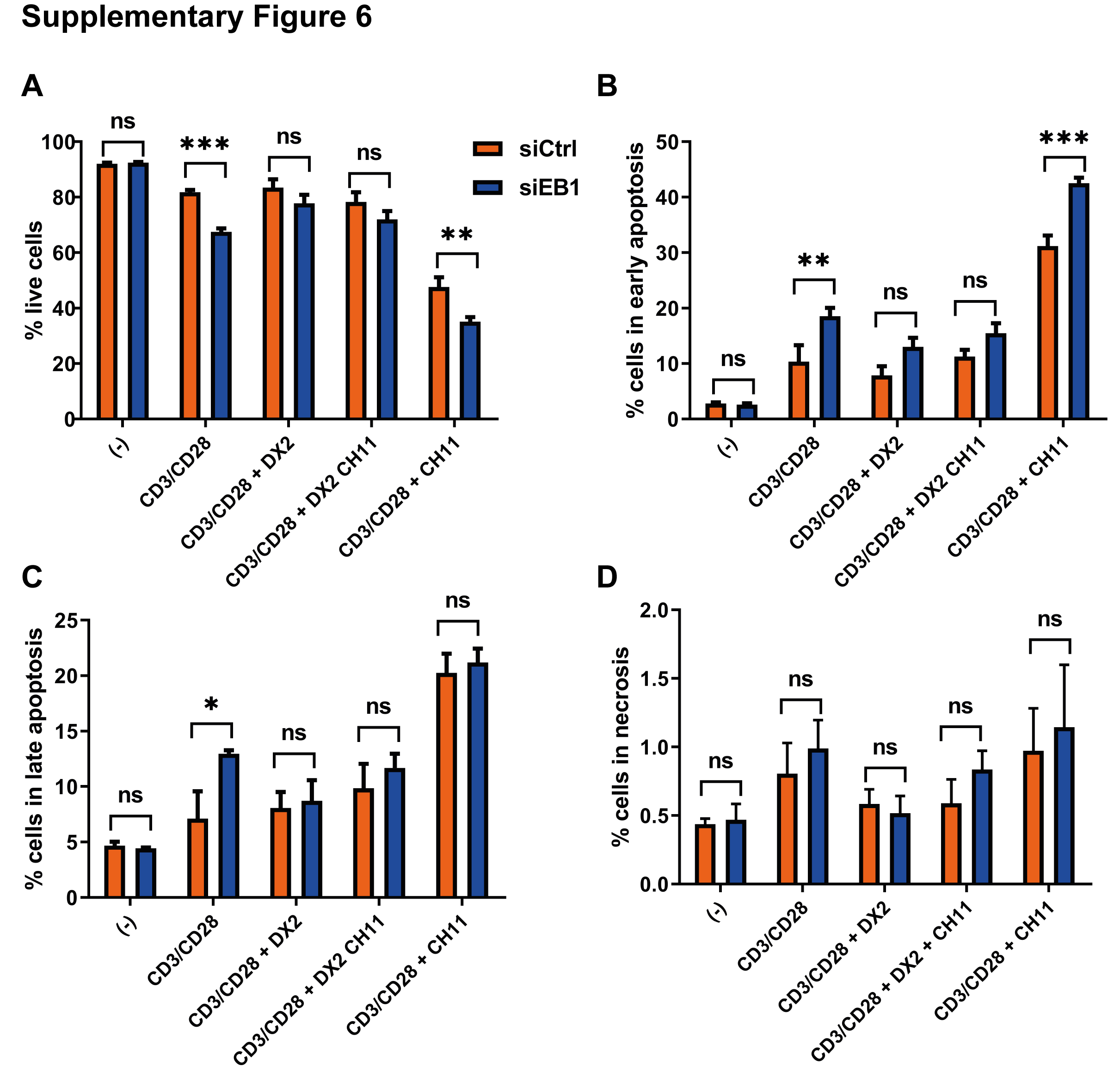

Supplement: Supplementary Figure 6 — Cell viability statistical analysis in JK E6-1 T cells. siCtrl; is shown in orange and EB-1 silenced (siEB1) is shown in blue. Non-stimulated cells (-); TCR activation with αCD3 and co-stimulation with αCD28 (CD3/CD28); CD3/CD28 activation plus anti-Fas antagonistic antibody, DX2 (CD3/CD28+DX2); CD3/CD28 activation plus DX2 and apoptotic inducer, CH11 (CD3/CD28+DX2+CH11), or CD3/CD28 activation plus CH-11 (CD3/CD28+CH-11). Graph shows (A) Percentage of live cells. (B) Percentage of cells in early apoptosis. (C) Percentage of cells in late apoptosis. (D) Percentage of necrotic cells. Data are mean ± SD, n=3. two-way ANOVA test. *, p<0.05; **, p<0.01; *** p<0.001; **** p<0.0001; ns, non-significant. Corresponds to Figure 7. [file Image_6.tif]
